# The Retail Food Sector and Indigenous Peoples in High-Income Countries: A Systematic Scoping Review

**DOI:** 10.3390/ijerph17238818

**Published:** 2020-11-27

**Authors:** Tiff-Annie Kenny, Matthew Little, Tad Lemieux, P. Joshua Griffin, Sonia D. Wesche, Yoshitaka Ota, Malek Batal, Hing Man Chan, Melanie Lemire

**Affiliations:** 1 Département de médecine sociale et préventive, Faculté de médecine, Université Laval, Quebec, QC G1V 0A6, Canada; Melanie.Lemire@CRCHUdeQuebec.uLaval.ca; 2 Centre de recherche du CHU de Québec, Université Laval, Axe santé des populations et pratiques optimales en santé, Quebec, QC G1E 6W2, Canada; 3 School of Public Health and Social Policy, University of Victoria, Victoria, BC V8P 5C2, Canada; MatthewLittle@uVic.ca; 4 Department of English Language and Literature, Carleton University, Ottawa, ON K1S 5B6, Canada; Tad.Lemieux@Carleton.ca; 5 School of Marine and Environmental Affairs, University of Washington, Seattle, WA 98105, USA; PJGriff@uW.edu (P.J.G.); Yota1@uW.edu (Y.O.); 6 Department of American Indian Studies, University of Washington, Seattle, WA 98195, USA; 7 Department of Geography, Environment and Geomatics, Faculty of Arts, University of Ottawa, Ottawa, ON K1N 6N5, Canada; SWesche@uOttawa.ca; 8 Nippon Foundation Ocean Nexus Center, EarthLab, University of Washington; Seattle, WA 98195, USA; 9 Département de nutrition, Faculté de médecine, Université de Montréal, Montreal, QC H3T 1J4, Canada; Malek.Batal@uMontreal.ca; 10 Centre de recherche en santé publique (CReSP), Montreal, Quebec, QC H3N 1X9, Canada; 11 Department of Biology, University of Ottawa, Ottawa, ON K1N 9A7, Canada; Laurie.Chan@uOttawa.ca; 12 Institut de biologie intégrative et des systèmes (IBIS), Université Laval, Quebec, QC G1V 0A6, Canada

**Keywords:** indigenous peoples, food environment, food price, food supply, food and nutrition, consumer, affordability, food security, obesity, health equity

## Abstract

Indigenous Peoples in high-income countries experience higher burdens of food insecurity, obesity, and diet-related health conditions compared to national averages. The objective of this systematic scoping review is to synthesize information from the published literature on the methods/approaches, findings, and scope for research and interventions on the retail food sector servicing Indigenous Peoples in high-income countries. A structured literature search in two major international databases yielded 139 relevant peer-reviewed articles from nine countries. Most research was conducted in Oceania and North America, and in rural and remote regions. Several convergent issues were identified across global regions including limited grocery store availability/access, heightened exposure to unhealthy food environments, inadequate market food supplies (i.e., high prices, limited availability, and poor quality), and common underlying structural factors including socio-economic inequality and colonialism. A list of actions that can modify the nature and structure of retailing systems to enhance the availability, accessibility, and quality of healthful foods is identified. While continuing to (re)align research with community priorities, international collaboration may foster enhanced opportunities to strengthen the evidence base for policy and practice and contribute to the amelioration of diet quality and health at the population level.

## 1. Introduction

Indigenous Peoples represent approximately 6% (476 million) of the world’s total population [1]. They reside in over 70 countries where diverse ecosystems have traditionally provided the foundation for diet, cultural identity, and social cohesion [2], and where they retain distinct social, cultural, economic, and political characteristics [3].

Despite the rich diversity of identities, histories, socio-economic, and environmental realities, Indigenous Peoples account for up to one-third of the world’s extremely poor, and some of the world’s most disadvantaged and marginalized peoples [4]. While international commitments to global health, such as the sustainable development goals (SDGs), were largely conceived around issues in low- and middle-income countries (LMICs) [5], pronounced health disparities among Indigenous Peoples persist in high-income countries (HICs).

In Canada [6] and Australia [7] for instance, Indigenous Peoples experience lower life expectancy, higher infant mortality rates, and a greater burden of chronic diseases such as obesity, Type 2 diabetes, and cardiovascular disease—conditions for which diet and nutrition are key determinants [8]. Marked disparities in food security also exist [9,10]. Health disparities in these contexts derive from socioeconomic inequality and systemic political disempowerment related to enduring legacies of colonization and contemporary neo-colonial influence [11]. Collectively, colonization, globalization, and development have resulted in challenges to land-based ways of life, and the increased adoption of a “Western” diet (i.e., high in saturated fats, sugar, and processed foods) [12,13,14,15]. While traditional foods remain strongly culturally preferred, market foods (in particular those of limited nutritional quality) represent a major fraction of contemporary diets [16,17,18,19].

Health disparities are particularly serious among Indigenous Peoples living in remote regions where the high price, low quality, and limited availability of nutritious perishable food is compounded by socioeconomic disadvantage to severely constrain food access/security [20,21,22]. The population-level diet of Indigenous people in remote regions is characteristically low in consumption of nutritious perishable market foods, such as fruits and vegetables [16,23]. Pervasive inadequacies and insufficiencies of dietary fiber, fatty acids, and several micronutrients are documented among Indigenous populations in remote areas [23,24]. Meanwhile, refined nonnutrient dense foods and beverages (hereafter beverages are captured with the term “food”) represent a significant fraction of the total diet, and of monetary expenditure on market food [19,25,26]. These issues are also significant health challenges for Indigenous Peoples in nonremote areas, such as in urban centers [10].

In most HICs, Indigenous Peoples are considered minority populations.. For example, in Canada and Australia, Indigenous Peoples comprise less than 5% of the total population [27]. Notable exceptions include Greenland and some Oceanic countries, where Inuit (90% of the population) and Pacific Islanders (Polynesians, Melanesians, Micronesians), respectively, form majority populations [27]. Many Indigenous communities, particularly those in rural/remote areas, have only one or two local stores (including small general/department stores, and nontraditional food retailers such as gas stations, convenience stores, and trading posts), while some have none. These stores are often small, service a small population base, and experience high operating costs and complex logistics. Stores are operated with various degrees of community governance and may have direct or indirect lineages to colonial enterprises—the Northwest Company, for example, “traces its roots back to 1668 with many... store locations in Northern Canada and Alaska having been in operation for over 200 years” [28]. In addition to food from the national and global agri-food sector, such stores may sell locally sourced/harvested food, as well as equipment and supplies for harvest and other goods (e.g., clothing). While individuals can place food orders through online retailers or travel (sometimes significant distances) to larger population centers with greater food diversity and lower prices, limited resources (e.g., access to a vehicle, credit card, and adequate food storage space) and capacity (e.g., time, internet access/literacy) may preclude or limit the use of nonlocal retailers, particularly for the most socioeconomically disadvantaged and/or marginalized.

The local retail food environment plays a key role in shaping food access and choice [29]. The food environment is the interface between food systems and diet. Inadequate food environments, such as grocery store inaccessibility, have been associated with poorer diet quality and obesity [30,31] and are believed to exert a particularly important influence in contexts where people experience food insecurity [32]. Food environments that provide adequate and/or excessive access to dietary energy, but lack essential micronutrients, represent a distinct concern for population health—including risks of both under-nutrition (i.e., dietary deficiencies related to inadequate intake of healthful foods) and over-nutrition (e.g., excessive caloric and sodium intake) [33,34,35]. To date, however, food environment research has predominantly been conducted in larger population centers of HICs [36,37], with a burgeoning literature in LMICs [38,39]. Research from the latter has shown that food environment and diet dynamics differ *between* countries due to variation in economic factors [39]. Such dynamics, therefore, also likely differ *within* countries where economic, geographic, and cultural contexts diverge significantly from national averages. Thus, literature published for national and general populations in HICs may not apply to Indigenous populations. Yet, retail food environments in these settings are a priority area for research, intervention, and policy as they may foster and exacerbate diet-related health inequities, food insecurity, and poverty [8,40,41,42]. Indeed, the High-Level Panel of Experts (HLPE) on food security and nutrition has recommended the need to promote nutrition-focused, policy-relevant research on food systems and take specific measures, to ensure that marginalized groups, including Indigenous Peoples, are able to access or achieve a sufficient, diverse, nutritious diet that is culturally appropriate [43].

The present article systematically synthesizes literature pertaining to the retail food sector and Indigenous Peoples in HICs. At the time of defining this review, establishing the search protocol, and undertaking the screening/data charting, there were no published syntheses of retail food environments relevant to Indigenous populations at an international scale. Two recent reviews [44,45] have addressed retail food environments as they pertain to Indigenous populations, globally, focusing on: the contribution of retail food environments to diets and nutrition-related health outcomes; the effectiveness of food and nutrition policies; and the incorporation of Indigenous methods and participation in such research. This review complements, and furthers, these important contributions by responding to the calls of HLPE, to understand the drivers and determinants of food environments using an interdisciplinary systems approach, and by drawing on the knowledge, experience, and insights of individuals such as community leaders [43]. In accordance, the “retail food environment” is here conceptualized as the combined **community food environment** (type, location, and accessibility of retail food outlets), the **consumer food environment** (what consumers encounter within and around retail food outlets, including relevant characteristics of nutritional qualities, nutritional information, affordability, promotions, placement, range of choices, and freshness), and **consumer characteristics** (relative convenience and desirability of food products, taking into consideration personal and cultural factors that influence an individuals’ actions within their environment). Meanwhile, *retail food sector* is here understood to include the retail food environment and the food supply chain.

This synthesis focuses, therefore, on mapping key concepts derived through diverse forms of evidence, and identifying common/divergent issues, best practices, and points of intervention and policy to improve retail food environments across global regions and contexts. We hypothesize that these contexts share commonalities in several structural factors that uniquely shape the food environment. Ultimately, the goal is to support communities, researchers, and policymakers in moving towards a more equity-oriented research and policy agenda for improving the retail food environments of Indigenous Peoples in HICs.

## 2. Methods

### 2.1. Context: Indigenous Peoples in HICs

**Figure 1:**
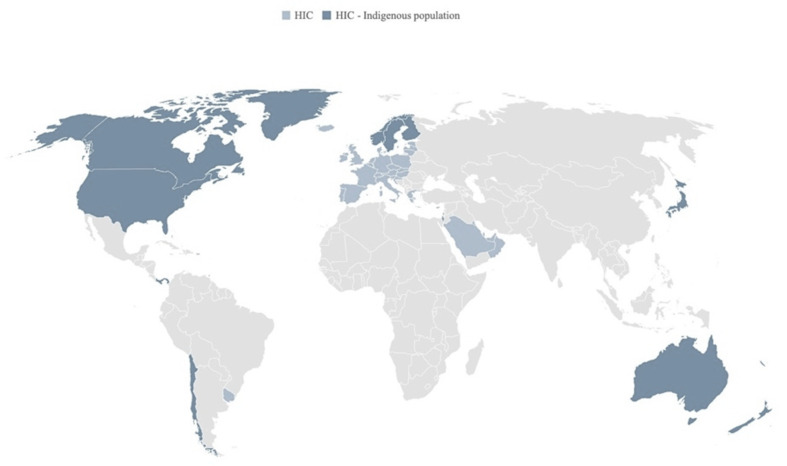
Map of countries with high income economies and Indigenous populations, as identified through the methods of this study. High-income countries are defined by the World Bank Atlas method (Gross national income (GNI) per capita of USD 12,376) for 2020. As of 2020, there are 80 HICs (including unincorporated/overseas territories) across six global regions.

This review draws on the breadth of published scholarly knowledge that exists on the retail food sector as it relates to Indigenous Peoples in HICs (Figure 1). A universal definition of “Indigenous” people has not been adopted by any UN-system body. Instead, the system has developed a contemporary understanding of this term based on several factors such as self-identification, historical continuity with pre-colonial and/or pre-settler societies, distinct social, economic or political systems, languages, cultures and beliefs [3]. In some countries, there may be preference for other terms including tribes, first peoples/nations, etc. Consistent with the United Nations recommendation of *identifying*, rather than *defining*, Indigenous Peoples [3], we systematically searched each HIC to identify Indigenous Peoples, following the approach described by Cisneros Montemayor et al. 2016 [46] (see Supplementary Material Figure S1). The objective was to define the scope of the review and to identify population-specific terms for the literature search. First, we conducted systematic searches by country in 2 major international databases: The World Directory of Minorities and Indigenous Peoples [47] and the eHRAF World Cultures database [48]. Populations of interest included both state-recognized and unrecognized ethnic/cultural groups that are the original or earliest known inhabitants of an area, and/or populations that maintain historical continuity with precolonial and/or pre-settler societies [3]. Database searches were complemented by referring to other key documents on Indigenous Peoples’ health—notably, the Lancet–Lowitja Institute Global Collaboration on Indigenous and tribal peoples’ health [49]. Ultimately 19 HICs with Indigenous populations across 5 global regions, were retained for this review (see Supplementary Material Table S1).

### 2.2. Literature Review

#### 2.2.1. Systematic Scoping Review

A systematic scoping review deemed appropriate for synthesizing a body of previously unreviewed literature [50] was undertaken following established protocols [51], and abiding by the Preferred Reporting Items for Systematic Reviews and Meta-Analyses-Extension for Scoping Reviews (PRISMA-ScR) guidelines [52]. Detailed methodology for the review is detailed in the Supplementary Material (Tables S2–S6).

#### 2.2.2. Search Strategy

The search strategy aimed to identify peer-reviewed publications involving the retail food sector and Indigenous Peoples in HICs. Two major online databases (Ovid MEDLINE and Scopus) were systematically searched using a combination of keywords pertaining to: i. Country; ii. Indigenous Peoples; and iii. the Food retail sector (see Supplementary Material Tables S3 and S4). Search terms were developed based on the results of Section 2.1 and a priori knowledge of the field and were refined through an iterative process. The search was conducted in August 2019 and updated in April 2020.

#### 2.2.3. Eligibility Criteria

The search was restricted to English language journal articles published over the last thirty years (1990–2020, inclusively)—a timeframe determined through an initial search with no date restrictions. To be eligible for inclusion, original peer-reviewed articles must have satisfied the geographic (HIC), populational (Indigenous population), and thematic focus (retail sector), as defined by the inclusion criteria (see Supplementary Material Table S5). Reviews, commentaries, study protocols, and grey literature were excluded. Articles focusing on other community/neighborhood food environment settings (e.g., schools) were excluded. Likewise, studies focusing exclusively on traditional foods and harvesting were excluded. Assessments of diet, food security, health, and psychosocial (e.g., knowledge and attitudes) factors, as well as community social, economic, and cultural conditions were excluded unless they incorporated a direct link to the retail food sector within study results.

#### 2.2.4. Screening

**Figure 2:**
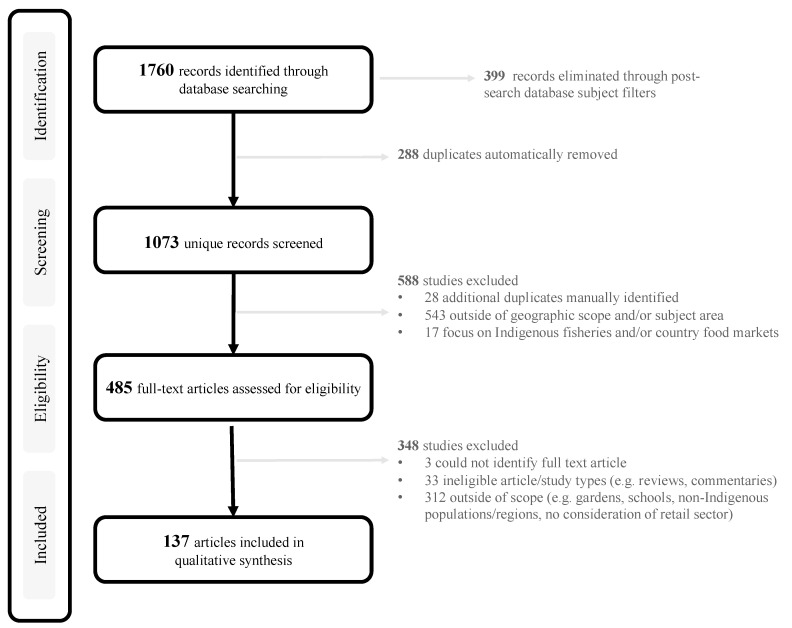
Preferred Reporting Items for Systematic Reviews and Meta-Analyses (PRISMA) flow diagram depicting the four-stage article process used identify, include and exclude (including and the reasons for exclusions) articles on the retail food sector and Indigenous Peoples in high income countries (HICs).

In total, 1073 records were identified from the search process following deduplication (n = 288) and database filters (n = 399) (Figure 2). In the first round of determination, 2 authors (TK and TL) independently screened the titles and abstracts of all records based on the eligibility criteria. The screening protocol and eligibility criteria were piloted on the first 50 articles and refined to ensure consistency. In the second round of determination, both authors independently scanned the full manuscript texts of the 485 retained articles to ascertain relevance, leading to the exclusion of an additional 348 articles. Ultimately 137 articles were included in the review.

#### 2.2.5. Data Charting

For each retained article, key geographic, populational, methodological, and topical characteristics were recorded by one author into an electronic spreadsheet (see Supplementary Material Table S6). Study attributes were tabulated by frequency, and figures were generated to summarize trends in the literature. The data abstraction scheme, including the identification/classification of major themes used to structure the review results, was developed by the first author through an iterative process that involved inductive and deductive reasoning and drew on existing frameworks of consumer food environments and food access [29,43,53,54]. Notably this is included in the conceptual framework of food systems for diets and nutrition presented by the HLPE [43]. Ultimately, retained articles were classified broadly, based on level of the retail food sector:

- Retail food supply, including supply chain (i.e., food processing, distribution, transport, warehousing); stores (i.e., characteristics of local food stores, including the geographic density/distribution, and vendor characteristics); food supply (i.e., items available in the store, including their availability (i.e., the presence and diversity of food items in the stores surveyed), affordability (function of food prices, income, and perceptions of value), quality/acceptability (i.e., structured assessments of product properties and perceptions about the appeal, value and convenience of the food supply) and in-store placement/promotion (e.g., shelf space allocation, labels and posters, announcements, etc.));
- Consumers (e.g., store sale records, shopping behavior)
- Interventions and initiatives (e.g., store-based and multisectoral interventions, food pricing policies and subsidies).

All data were charted by 1 author (TK), and 20% of entries were double entered by a second author (TL) to ensure validity.

## 3. Results

### 3.1. Overview of Included Studies

**Figure 3:**
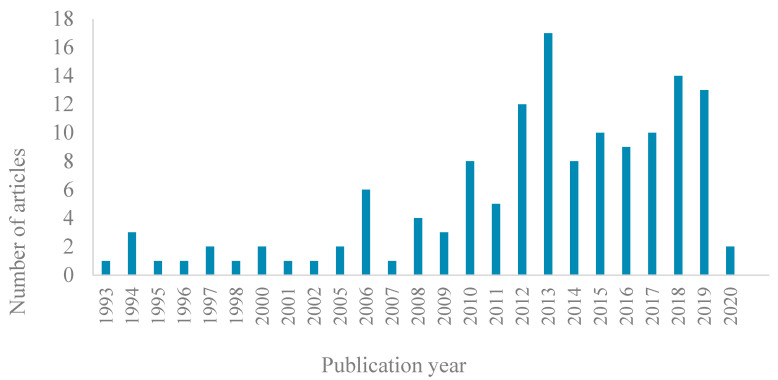
Overview of the literature by year of publication.

Key characteristics of the 137 articles included in the review are summarized in the Supplementary Material (see Supplementary Material Table S7). The number of relevant articles has increased over the last 30 years, with approximately half of all studies published in the last 5 years (2014–2019) (Figure 3).

#### 3.1.1. Where has the research been conducted, and which populations have been involved?

**Figure 4:**
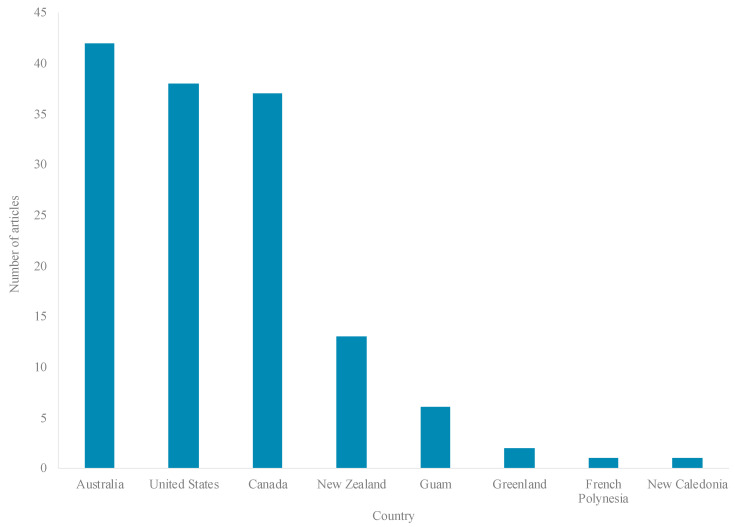
Overview of the literature by country.

**Figure 5:**
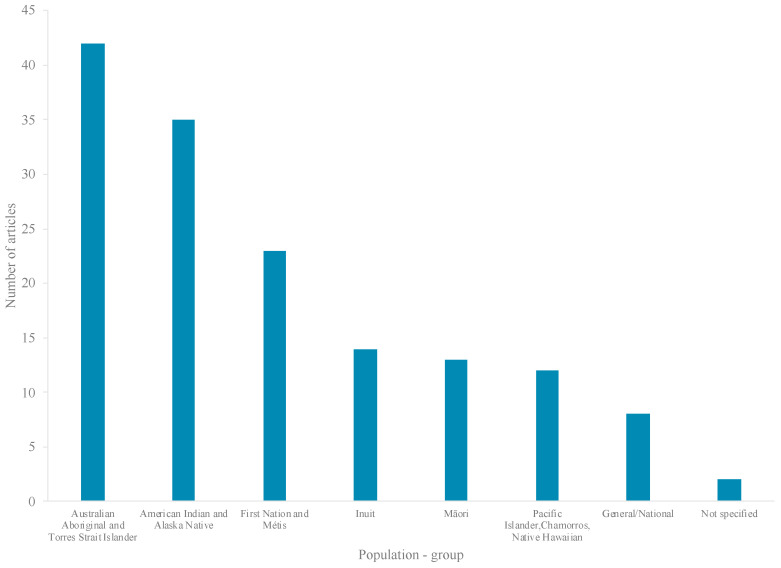
Overview of the literature by populational focus—Indigenous Peoples concerned.

**Figure 6:**
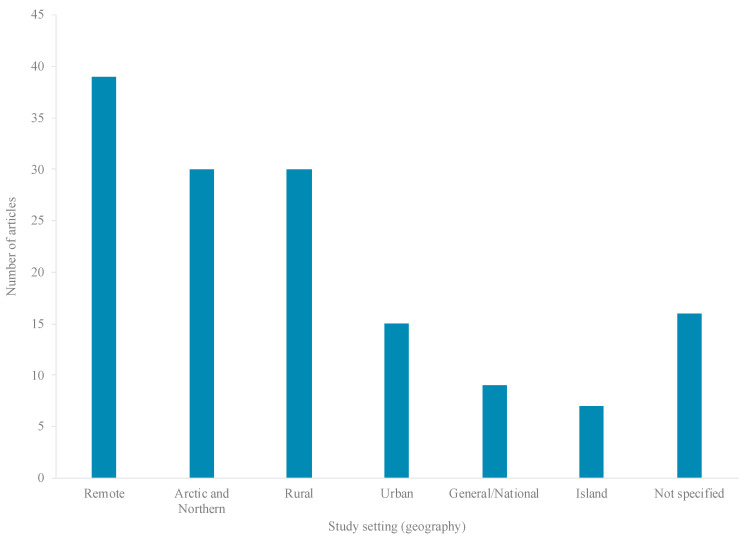
Overview of the literature by geography (as defined in the article).

Published literature was available from less than half (8 countries) of the 19 HICs eligible for inclusion in the review (Figure 4), 95% of studies conducted in four countries: Australia (31%; 42 articles), the United States (US) (28%; 38 articles), Canada (27%; 37 articles), and Aotearoa/New Zealand (NZ) (9%; 13 articles) (Figure 4). Literature pertained to five major Indigenous groups, including: Aboriginal and Torres Strait Islanders (42 articles), American Indians (AIs) (35 articles), First Nations (FNs) (20 articles), and Inuit (14 articles) (Figure 5). Most research (99 articles) was set in rural, remote, and/or northern/Arctic regions and involved distinct Indigenous communities or populations (Figure 4 and Figure 6). Few studies were conducted in urban areas (15 articles) and/or involved multiethnic populations (Figure 4 and Figure 6). Importantly, the definitions and use of the term’s “*rurality*”, “*remoteness*”, and “*northern*”, can vary considerably across studies, countries and contexts, based on technical and social factors. Nevertheless, remoteness is generally understood in terms of geography and access to health, education, energy supply, and other public and private services, each of which are often most highly concentrated in major contemporary population centers.

#### 3.1.2. How Has the Research Been Undertaken (Indigenous Participation and Study Design)?

Most research has been undertaken through collaborative and participatory processes involving Indigenous communities and organizations. A smaller number of studies either do not specify Indigenous participation and/or involve desktop research (e.g., secondary data analyses). Most studies involved quantitative study designs (55%; 76 articles) with fewer qualitative (30%; 41 articles) and mixed/multi-method approaches (15%; 20 articles) (Table S7).

#### 3.1.3. What Dimensions and Domains of the Retail Food Sector Have Been Examined?

Most studies (60 articles) focused on initiatives (policies, programs, and community planning/prioritization) and interventions to improve community health and/or food systems involving the retail sector. Many studies also focused on consumer perceptions and behavior (45 articles) related to the market food supply, store access, and food purchasing. A smaller body of literature focused on assessments of the food supply (33 articles), store-level factors, and the retail workforce (18 articles) (Table S7).

### 3.2. Retail Food Sector—Food Supply Chains and Food Imports

The retail food supply chain has largely been included as a descriptive or contextual factor in the literature with limited research explicitly focused on this domain [55]. Qualitative studies documenting perspectives from the local retail workforce (store owners/managers and distributors) and community members highlight several common issues affecting food supplies across remote Indigenous communities in Australia, Canada, Greenland, and the US [22,56,57,58,59,60]. These include transportation logistics and costs, inadequate local infrastructure, and operating challenges.

Despite community/store policies, and interest on behalf of both community members and store managers to procure food from local and Indigenous producers, in practice several constraints (e.g., decision-making authority, supply chain logistics) can restrict this possibility [58,61,62]. Meanwhile, local producers can experience challenges in selling to small local stores compared to central distributors (e.g., higher costs, lower turnover), and/or may be unable to supply requisite volumes [61,63]. Furthermore, the provenance of store foods may be driven by broader factors such as globalization and economic/political relationships. In Guam, for example, available (processed) food has derived from an increasing number of countries over time; however, most products originate from the US, which of which Guam is an “unincorporated territory” [64].

### 3.3. Retail Food Sector—Food Stores

A total of nine articles mapped and/or inventoried the number/density, type, and/or location of food stores in (or in proximity to) Indigenous communities [65,66,67,68,69,70] (Table 1). These were based on both empirical (e.g., existing data sources, on-site observations) and respondent-based methods involving community knowledge/perceptions (e.g., asset mapping). A single study used both empirical and respondent-based methods with convergent results between approaches [65]. These approaches are complementary to studies which document consumer experiences and perceptions of store access and shopping behaviors (see Section 3.5).

#### 3.3.1. Store Availability and Geographic Accessibility (Type, Number, and/or Location of Stores)

Several studies conducted in North America highlight issues of store availability in Indigenous communities—notably, the absence or limited availability of supermarkets and grocery stores, and, in several cases, the presence of nontraditional food retailers such as gas stations and convenience stores, particularly in AI and FN reservations (see for example [65,67,68,71,73]). In one study, tribal areas in the US had significantly lower densities of healthy food outlets compared to nontribal areas even after controlling for socio-economic and demographic variables [65]. Meanwhile, in a multi-ethnic urban setting (Edmonton, Alberta), supermarket density did not differ in neighborhoods with a greater percentage of Indigenous residents [69]. Although mapping community location and the distribution/density of stores provides insights into potential geographic barriers to store access, consumer surveys (e.g., shopping behavior, transportation access) are requisite to appraising store accessibility—including heterogeneous (and socially-patterned) experiences of store access across populations. Issues of store availability in northern Canada were often described in terms of limited retail competition (monopolies or oligopolies) [62,66,74]. One study that directly examined this issue (i.e., presence of a second retailer by community) in the provincial north of Canada found that over 90% of remote FN communities surveyed are serviced by a single corporate food retailer [66]. This can be problematic, as communities with a single food retailer exhibit higher food costs, and though the presence of a second retailer may still not render food prices affordable, increased competition of grocery stores has been associated with better food pricing and quality [74,75].

#### 3.3.2. Vendor Characteristics (Store Operation and Management)

An additional attribute of local stores which may impact local food supplies involves store operating practices/philosophies, community governance, and involvement of the health sector in defining retail practices and policies—all of which vary markedly across stores and regions. The need to include Indigenous priorities in store management and operations is highlighted in several studies—including the need for ongoing communication between stores and communities, community co-operatives, and Indigenous-owned businesses [56,63,70]. Many stores in Indigenous communities, however, are associated with corporate chains and/or are managed by non-Indigenous people [71,76]. While store managers may acknowledge their role in the local food supply, and recognize the financial constraints of community members, several factors such as manager ideologies, supply chain challenges, and constrained managerial authority/choices may influence their stocking practices [22,56,57,58,77]. Furthermore, despite the potential benefits of establishing collaboration and building capacity between the retail and health sectors [78], as evidenced in a number of policies and intervention studies (e.g., appointment by the Looma Community Council (remote Australian Aboriginal community) of a store manager with a mandate to improve the food supply [79]), the role of the public health sector and local nutritionists may not be comprehensively apprehended or appreciated by the retail sector [22,56]. Moreover, although store owners and managers have shown willingness to participate in health-related interventions, they may do so provided it does not consume store resources and employee time [80]. The financial effects of health-related policies on retail performance remains poorly understood [81].

#### 3.3.3. Relating Store-Level Factors to Food Supply and Health

Store level factors (including store type/size and management/operating practices) have been related to both the food supply and consumer health among Indigenous Peoples in HICs. Small stores and nontraditional food retailers tend have higher prices and carry fewer healthful items such as fresh produce and lower-sodium products than do larger stores and supermarkets, which tend to be less present in these settings [56,67,71,73,77,82,83]. For example, in a study conducted on an AI reservation, convenience stores carried approximately one-fifth of the items in a standard checklist (compared to 86% of items in supermarkets) [73]. Even still, across all store types, on-reservation stores had, on average, roughly half of the items on the checklist, with significant disparities in fresh produce availability [73]. Relatedly, proximity to convenience stores and high reliance on nontraditional food retailers has been associated with weight-related variables and diabetes [72,84], although consumption of food from nontraditional retailers has been inconsistently associated with food security status [85,86].

### 3.4. Retail Food Sector—Food Supply

Food supply assessments have included both empirical store audits/checklists (15 articles), as well as respondent-based assessments of the perceived food supply (>20 articles) (Table 2 and Table 3). The former has mostly involved cross-sectional study designs based on standardized tools, such as the Nutrition Environment Measures Survey in stores (NEMS-S) scoring system and other predefined healthful market baskets, variously adapted to local contexts [87]. Among empirical food environment assessments, most examined food availability (10 articles) and cost (12 articles), (Table 2). Meanwhile, in qualitative respondent-based studies, various dimensions of the food supply are discussed collectively (i.e., that healthful food is expensive, of poor quality, and limitedly available), and include consideration of both healthful and discretionary foods, as well as impacts these factors have on community diet and health [59,62,88].

#### 3.4.1. Food Availability

In several studies, community members perceive deficits in the availability and selection of healthful (notably, nutritious perishable, bulk, and special dietary) foods, contrasted by the overabundance of nutrient-poor discretionary food items [57,63,88]. This deficit is perceived as a barrier to health [89], and the “right to food” for people with pre-existing health conditions, such as diabetes [62]. There is also concern regarding the availability of foods for specialized needs and diets, such as high-iron infant foods [90]. While qualitative, respondent-based studies highlight the ubiquity of non-nutrient dense foods in local stores [67], few empirical studies have examined the availability of such items in these contexts.

Store food availability audits conducted in Australia [91], Canada [92], Greenland [56], Guam [82], and the US [71,73,83,93,94] also capture these issues, with dramatic disparities in northern and remote stores. In Greenland, for example, some remote stores do not carry any fresh items (e.g., produce, dairy, and/or meat) [56] and variety can be extremely limited among those that do. Results are similar in Alaska [93], where less than half of fruits (20–40%) and vegetables (20–30%) in a standard checklist are available, and in Guam [82], where less than half (47%) of stores surveyed sold more than two varieties of fresh fruit.

#### 3.4.2. Food Affordability

In respondent-based studies, community members across several global regions describe food, particularly fresh healthful items, as being overly-priced and prohibitively expensive [57,62,63,89,95,96,97]—especially when compared to less healthful options like processed/convenience foods. Participants also perceive variations in prices between local and out-of-town food stores [59], including higher prices on-reserve vs. off-reserve [98]. Participants have reported that the unaffordability of healthful food is a barrier to improving their diets [96] and, indeed, higher rates of obesity have been documented among participants who report that the price of fruits and vegetables is cost-prohibitive [84]. Similarly, high food costs have been associated with greater likelihoods of adult and child food insecurity among American Indian adults [85]. While issues of food affordability are discussed by study respondents in terms of their implications for socio-economically vulnerable community members, including those on income support [57,99], empirical studies have typically been restricted to assessments of food costs with limited consideration of income and basic living expenses (e.g., housing) [100].

The relative price between more/less healthful food (e.g., nutrient/energy density, reduced-sodium items [77]) is observed in empirical pricing studies [26,101], with a few exceptions [82]. The higher relative cost of healthful vs. less healthful foods—such as the price of water compared to sugar-sweetened beverages—has been associated with health outcomes like obesity among Māori [87]. Empirical pricing surveys conducted across global regions also consistently document significantly higher food prices in remote Indigenous communities compared to referent locations (e.g., capital cities, national averages). Food prices in Indigenous communities in remote Australia [102] and the Canadian Arctic [26] were over 60% higher than referent locations. Price disparities have been associated with community and geographic factors such as road access [91,103]. Remoteness category, for example, explained over half (58%) of the total variance in food basket price in Queensland (Australia), but was less marked for produce than for other food groups such as dairy and meat [91]. It is unclear how these dynamics manifest in rural settings. In a food pricing study conducted in a large rural AI community, the cost of purchasing a market basket ranged between -3% +24% relative to the national average between stores [83]. Other contextual factors such as season and store nutrition policies are less examined in the literature but are believed to play a role in food costs [91,103]. For example, changes in food prices in northern First Nation communities (Canada) were two times higher between fall and winter than in the provincial capital [103]. In Australia, relative improvements in food prices have been seen over time in very remote stores, hypothesized to be related to factors such as the implementation of store nutrition policies and quality retailing practices [91].

Food prices collected from local retail outlets have, in combination with purchase and eating patterns described in population-based surveys, also been used to estimate actual and theoretical diet costs, and demand elasticities using diet optimization and econometric models [19,25,104,105,106,107,108,109,110]. In the Canadian Arctic, remote Australian Aboriginal communities, and among Māori and Pacific households, non-nutrient-dense foods account for one-to-two-thirds of estimated diet costs [25,105,106]. Due to high consumption of discretionary items, the cost of theoretical diets modelled to meet nutritional requirements for Aboriginal and Torres Strait Islander and Māori and Pacific households in NZ is often similar and, in some cases, less expensive than current, less-healthful, diets [104,105,106]. Importantly, current and theoretical diets may remain unaffordable for some households, notably those on income support [104].

#### 3.4.3. Food Quality

Issues of food quality have largely been ascertained by surveying community perspectives of the food supply; few store audits involving food quality indicators appear in the literature. Community members in several global regions express concerns related to the quality and freshness of available foods, including the presence of past-date and expired items [57,62,66,84], although the ubiquity of such items has been limitedly examined in empirical store-based assessments. These perceptions may affect purchasing behavior (see Section 3.5) as some consumers may avoid purchasing fresh items for their short shelf-life and for the risk that they may be moldy—particularly in light of less expensive foods with longer shelf lives [57]. In store food-quality assessments conducted in Australia, approximately one-third (30%) of very remote stores did not meet quality criteria for fresh produce [111]. A notable exception was for oranges, believed to be due to local production. Similarly, in Guam, produce was rated ‘‘unacceptable’’ for 25–50% of fruits and up to two-thirds of vegetables [93].

#### 3.4.4. Point of Purchase Promotion and Information

While community members have expressed concerns regarding what is sold and how it is promoted [63], and point-of-purchase media constitutes a main dimension of store-based public health interventions in Indigenous communities in both the US and Canada (see Section 3.6), few observational studies have examined these factors in the literature, and most have emphasized nutritional labels—notably the importance of culturally/ethnically appropriate information in Indigenous languages [56,89,112]. Among available studies, store signage in Guam more commonly promoted less-healthy eating than healthy eating [82].

**Table 2:** Summary of literature involving empirical food supply assessments

Reference	Setting ^1^		Store(s) Surveyed		Methods		Food Supply ^2^				Connection to Diet and Health
	Country	Geography	Number of Stores/Communities	Store Type	Timepoints	Survey Tools ^3^ (Number and/or Type of Items)	Availability	Affordability (Cost)	Quality	Point of Purchase Promotion	
[94]	US	Rural	18/2	Convenience store	Single	NEMS-TCS (ready to eat foods)	✓	✓	✓	✓	
[83]	US	Rural	27/1	Several store types	Single	NEMS-S (68 items)	✓	✓			
[87]	NZ	Rural; Urban	392/98	Supermarket	Single	NEMS-S (5 items—regular vs. healthier choice)	✓	✓ Relative price			✓ BMI
[82]	GU	Not specified	114/	Large and small stores	Single	NEMS-S (Healthful and less healthful)	✓	✓ Relative price		✓	
[26]	CA	Arctic and northern	/6	Community stores	Seasonal	RNFB (+items based on local diets)		✓ Nutrition economics	✓ Not reported		
[93]	US	Arctic and northern	/13	Community stores	Single	NEMS-S (Fresh produce only) Alaska Food Cost Survey	✓	✓	✓		BMI and diet reported but not related to food supply
[73]	US	Not specified. Comparison on vs. off reservation	50/22	Several store types	Single	TFP market basket (68 items)	✓	✓			
[91]	AU	Several categories	92/	Not specified (stores previously surveyed)	Single (compared to 1998)	HFAB	✓	✓			
[111]	AU	Several categories	144/	Grocery stores and community stores	Single	HFAB (430 items costed;13 items for quality)		✓	✓ Based on industry standards		
[71]	US	Rural; Remote	72/	Several store types	Single	NEMS-S (Healthful and less healthful)	✓	✓ Relative price		✓	
[64]	GU, NC	Capital city	Country-level	Large stores (or chains)	Single	Protocol based on collaboration on nutrients in processed foods (3438 items)	✓ Country of origin			✓ Food labels (nutrient data, promotional claims)	
[77]	GU	Not specified	100/	Large and small stores	Single	In style of NEMS-S (9 items)			✓ Sodium content		
[102]	AU	Remote-compared to capital cities	20/	Community stores	Single	453 items (63% of food expenditure)		✓			
[103]	CA	Remote-compared to capital city	/3	Community stores	Fall and winter	TNFB + additional foods (22 items)		✓			
[56]	GL	Arctic and northern	5/5	Community stores	Single	NEMS-S Freedman Grocery Store Survey	✓				

^1^ Countries: AU = Australia; CA = Canada; GL = Greenland; GU = Guam; NC = New Caledonia; NZ = New Zealand; US = United States; Geographic setting as self-defined in the article. ‘Arctic and northern’ includes the provincial North of Canada; ^2^ Checkmark indicates studies which assessed the dimension of the food supply named; ^3^ HFAB = Healthy Food Access Basket; NEMS-TCS= Nutrition Environment Measures Survey for Tribal Convenience; NEMS-S=Nutrition Environment Measures Survey for Stores; RNFB= Revised Northern Food Basket; TFP = Thrifty food plan; TNFB= Thrifty Nutritious Food Basket (Agriculture Canada).

**Table 3:** Summary of respondent-based studies that highlight issues of food supply and consumer experiences.

Reference	Setting ^1^		Participants	Food Supply ^2^				Consumer and Shopping-Related Issues Discussed by Respondents
	Country	Geography		Availability	Affordability	Quality	Point of Purchase Promotion	
[113]	CA	Arctic and northern	Dene/Métis adults	✓	✓ Expensive	✓ Lack of freshness		
[59]	US	Rural	- Primary household shoppers (American Indian) - Other stakeholders	✓	✓ Higher cost of healthy food	✓ Quality of meat		- Shopping location - Care access - Government assistance programs (on the type and timing of foods purchase)
[96]	CA	Urban	Caregivers of Métis and off-reserve First Nations children		✓ Unaffordability of both healthy and unhealthy			- Reliance on energy-dense, nutrient-poor foods, as these tended to be more affordable and lasted longer than more nutritious, fresh food options - Transportation-related issues
[98]	CA	Six Nations of the Grand River	Adults (from Six Nations Reserve)	✓	✓ CAD 151/week to feed household			- Shopping location and frequency
[89]	NZ	Auckland and Wellington	Māori and Pacific shoppers	✓	✓ Higher cost of healthy food		✓	- Difficulty changing shopping behavior / habit - Cost as a major barrier
[84]	US	Rural	American Indian adults	✓	✓	✓		- Frequency and location of shopping
[114]	US	Urban; Rural	Tribal leaders (American Indian)					- Shopping location
[115]	AU	Remote	Adults (Aboriginal)		✓ High cost of food and competing demands for money			- Long-shelf-life food - Pay cycles - Available funds purchase less more expensive healthful foods
[60]	CA	Arctic and northern	- Inuit adults - Other stakeholders	✓	✓	✓		- High price making it challenging to obtain food of sufficient quality
[116]	CA	Arctic and northern	- Inuit women - Other stakeholders	✓	✓	✓		
[117]	US	Urban	American Indian women					- Environmental constructs related to food purchasing, behaviour and body mass index
[57]	CA	Arctic and northern	- Inuit adults - Other stakeholders		✓	✓		- Cost and quality main barriers to purchasing
[88]	US	Navajo Nation	- Parents - Other stakeholders	✓ Predominant foods available are convenient and unhealthy	✓			- Shopping when monthly support checks are distributed
[62]	CA	Arctic and northern	Community members	✓	✓	✓		- Location of food purchase
[118]	CA	Arctic and northern	- Inuit women - Other stakeholders		✓			
[95]	CA	Arctic and northern	Indigenous women (First Nation, Dene/Métis, Inuit)		✓			
[119]	CA	Arctic and northern (Rural)	Adults (First Nations)					- Location of food purchase

^1^ Countries: AU = Australia; CA = Canada; GL = Greenland; GU = Guam; NC = New Caledonia; NZ = New Zealand; US = United States; Geographic setting as self-defined in the article. ‘Arctic and northern’ includes the provincial North of Canada. If geographic setting was not stated in the study, the location of the intervention is included. ^2^ Checkmark indicates studies which respondents discussed issues related to the dimension of the food supply named.

### 3.5. Consumers

Several articles examined consumer-related factors, including shopping behavior (e.g., shopping frequency, location, and cycles), factors influencing purchase (e.g., psychosocial factors), and the use of store-sales records (e.g., as proxies for community diet and in intervention studies). Studies on consumer-related factors were available from North America and Australia, exclusively.

#### 3.5.1. Shopping Location (in/out of Community and by Store Type)

In several studies, a high percentage of community members report shopping outside the reserve/community, sometimes travelling considerable distances (an hour or more one way) to access supermarkets/grocery stores [59,98,120,121]. Still, challenges related to food store access, and a high reliance on foods purchased locally is reported in several studies [63,97,98,122]. For example, most (58%) Havasupai adults (and in particular, older adults) report consuming only food purchased on the reservation [122]. Transportation-related barriers (e.g., lack of vehicle access and/or time or money for fuel), often related to socio-economic circumstance, are cited by community members in several studies as barriers to shopping and purchasing healthful foods at locations beyond the community that might have a wider selection of foods at lower prices [59,62,63,96,121,123]. Other factors influencing “out-shopping” include geography (e.g., the more remote a community, the fewer residents report consuming purchasing food outside the community [124]) and seasonality (e.g., availability and conditions of roads [103], including seasonal roads like ice roads. Though store access challenges are pronounced for people in remote areas, these issues are also noted in studies involving Indigenous Peoples in nonremote areas—notably, for individuals who rely on public transportation [96], as well as individuals who live further away from town centers or food retailers [59], including those who reside in social housing [62]. As noted previously, given the relationship between store type and food supply, understanding consumer use of different store types is relevant to public health, particularly since convenience stores are a primary source of food for some community members [119]. Consumers shopping in grocery stores and trading posts on Navajo Nation land, for example, were significantly more likely (520% and 120% higher odds, respectively) to purchase produce than customers shopping in convenience stores [125]. Nevertheless, some consumers may favor shopping in stores where credit is available (e.g., trading posts), even though food may be more expensive [120]. Meanwhile, AI children from food-insecure households were more likely to eat some less healthful types of foods, including items purchased at convenience stores [86]. As most studies have relied on observational designs, they cannot distinguish the direction of association.

#### 3.5.2. Shopping Frequency and Cycles

Five studies highlight the importance of describing shopping frequency and cycles—including their drivers, such as cycles of income (e.g., arrival of social support payments [59,126,127], wildlife harvesting [128], food shipments [129], and store access—as these may influence patterns of food purchase, particularly for healthful perishable items (e.g., individuals who shop infrequently may favor purchasing items with longer shelf-lives [96]). Shopping patterns also relate to food security status [59]. For example, community members in Australia and the USA reported purchasing fresh fruits and vegetables almost exclusively in the week(s) when income support checks are distributed [59,115]. As families run out of funds during off-pay weeks, they report relying on foods with long shelf lives, and foods that are inexpensive and often less healthful [59,115]. By contrast, a study examining patterns of food purchasing in a remote Australian Aboriginal community found no association between consumer economic constraints and purchasing but did find a significant association between time since the last shop delivery (defined as food scarcity) and money spent in the shop [129].

#### 3.5.3. Consumer Decision-Making

Diverse factors influence the type of foods that individuals/families purchase and consume, including personal (e.g., taste/food preference, familiarity and purchasing habits, ease/convenience, intentions, intolerance to certain items, specialized diets), household (e.g., the presence of children), and food supply (e.g., food cost, availability, and quality, as well as marketing and promotions such as coupons) factors (see for example [57,59,89,113,116,119,130]. In particular, several studies emphasize consumer decision-making in relation to food supply constraints (e.g., high cost of food) and financial strain (e.g., low income, competing demands for money like high electricity bills) [100,115,131]. Many participants reported that high price was a barrier to buying healthful food [89] and that, contrary to their preferences, they purchased cheaper processed and/or low-cost brands, as these were perceived to be more affordable and/or longer-lasting than fresher, more nutritious options [57,89,115,131] Furthermore, one study reported that such lower-cost options ensured that children had something to eat at each meal when “money was tight” [100]. Some participants indicated that they would only buy healthier food if the benefit was two-fold (i.e., the price cheaper, and the food healthier) [89].

#### 3.5.4. Store Sales Records

Store sales records have been used for several decades to assess the nutrient quality of local food supplies and as a proxy for consumption (i.e., ‘apparent diet’ through the ‘store turnover method’) among Indigenous People in remote communities in Australia (10 articles) [19,23,79,101,124,132,133,134,135,136]. This method has been shown to yield high congruence with classic dietary assessments and has been validated against nutritional biochemical indicators [133,137]. When compared with other dietary intake methods, store sales records are reported to hold several advantages, including greater acceptability among community members, reduced potential for bias, relative objectivity, being minimally invasive, speed, ease of data collection, and low cost [133,137]. While the structure of the local food supplies is changing in remote Indigenous communities [138], proxy dietary estimates derived from stores closely align with those obtained from a complete set of community food providers [135]. This approach has shown to be a viable way to measure the effects of interventions on food purchases in supermarkets [136] and to assess changes in consumption and food preparation methods over time [79].

### 3.6. Improving the Retail Sector

#### 3.6.1. Food System/Security/Sovereignty Priority Setting and Planning

Several studies (12 articles) document community and multi-stakeholder priorities identified through participatory public health and food system/security/sovereignty planning and priority-setting activities [139,140,141,142,143,144,145,146,147,148,149,150]. These processes highlight several common desired improvements in retail food sectors across several global regions—including the need for improvements in the location of supermarkets, mobile venders, food transportation/delivery, infrastructure, retail competition, store management practices and policies, and subsidies to reduce cost and improve availability, among others addressing equity. Providing access to healthful food, at prices comparable to elsewhere in the country, was also identified as a policy priority [151].

#### 3.6.2. Multi-Sector/Strategies Involving the Retail Sector

Several multistrategy/sector public health interventions (e.g., involving food stores, schools, healthcare providers)—such as Apache Healthy Stores [152,153], Healthy Foods North [154,155,156], Navajo Healthy Stores [157], the Healthy Navajo Stores Initiative [125], the Tribal Health and Resilience in Vulnerable Environments (THRIVE) [61,94], Zhiwaapenewin Akino!Maagewin [158,159], OPREVENT [160]—have been implemented in Indigenous communities, notably in rural and remote areas across North America (Table 4). A number of interventions, including Healthy Foods Hawaii [161] and the Child Health Initiative for Lifelong Eating and Exercise (CHILE) [80,162] have also been conducted in ethnically diverse populations with a preponderance of minority and low-income consumers [136,163,164]. In several cases, these initiatives were developed through collaboration and multisectoral partnerships, and by integrating the results of formative research [59,90,153,154,162].

These interventions worked with local stores to improve the food-purchasing environment—namely, by enhancing the availability of healthful foods (including specialty or priority items such as healthful ready-made meals and snacks), and/or their placement (e.g., placing fruit at the checkout) and point-of-purchase promotions (e.g., placing stickers on shelves at eye level, organizing tasting demonstrations, providing recipe cards, and undertaking cooking demonstrations) [80,90,152,154,155,161,165,166,167] (Table 4). Few interventions focused directly on reducing food cost (see Section 3.6.3); nevertheless, food affordability may have been targeted indirectly by enhancing the selection and promotion of affordable healthful items [152]. One intervention (Healthy Navajo Stores Initiative) sought to enhance food quality by offering staff training on produce handling to maintain freshness and promote longevity [125], while another (Healthy Foods Hawaii) involved local producers/distributors to enhance the availability of local food [161]. While most interventions focused on promoting select/priority healthful foods, others targeted the demotion of discretionary items. The Healthy Communities Project, which successfully decreased the sales volume of sugar-sweetened beverages in an Aboriginal and Torres Strait Islander population, is one example [168]. Process evaluations suggest such interventions are implemented with different levels of dose, reach, and fidelity [80,152,158] while outcome evaluations have demonstrated positive changes in several individual outcomes, including psychosocial (e.g., increased food literacy, perception that healthful foods are convenient, food-related self-efficacy, and intentions) and behavioral measures (e.g., healthful food acquisition and purchase of healthful items like fruits and vegetables), as well as indicators of diet quality and health (e.g., nutritional biomarkers, reduction of adult body mass index (BMI)) [79,125,157,159,161,166]. Nevertheless, several interventions did not yield significant results on body weight and energy, sugar, or fat consumption. Moreover, most interventions were conducted as pilot/temporary projects and/or assessed outcomes at a single time point. However, one intervention to provide healthier choices for children demonstrated an improvement in the quality of foods purchased by children at the time of a second survey, two years after the initial intervention [165].

#### 3.6.3. Food Pricing Policies (Discounts/Subsidies, Taxes, and Vouchers)

Price discounts and subsidies provide economic incentives for consumers to purchase more healthful food and may have the potential to improve population-level diets [151,169]. These approaches have been viewed positively by community members in available studies [169,170]; however, there may be a number of challenges related to their implementation (e.g., limited local nutrition/store workforce capacity). Meanwhile, taxation (consumer and import taxes) on products such as sugar-sweetened beverages and confectionaries may be used to disincentivize purchase and/or to raise funds for health promotion, such as has been done in the South Pacific [171]. Studies regarding food pricing policies and strategies across global regions have involved different designs (e.g., government and store program/policy evaluations, feasibility studies, randomized control trials, epidemiological models) reflecting differences in the availability and delivery of such initiatives across global regions. In Canada, for example, where the Nutrition North Canada government subsidy is available to retailers operating in remote northern communities, evaluation research is limited, but underscores issues with the program’s accountability structure (e.g., gaps in food cost reporting), and absence of price caps and other means of ensuring food is affordable and equitably priced in communities [74]. In contrast, in settings where such policies/initiatives do not exist, and/or are not implemented comprehensively by communities, government, or retailers, intervention and modelling studies are reported in the literature. Notably, two randomized controlled trials (and related pilot studies)—Stores Healthy Options Program (SHOP) [112,136,164,172] and SHOP@RIC (Remote Indigenous Communities) [81] involving Māori and Pacific Islander populations (NZ) and Australian Aboriginal populations, respectively—assessed the effectiveness of price discounts on food purchasing, both when paired with nutrition education and without. These demonstrated an association between price discounts and healthy food purchasing; however, either no, or a small additive effect, was recorded when combined with nutrition education, and no effect was seen, with nutrition education alone [81,164]. The Healthy Choice Rewards program, a mixed methods study to investigate the feasibility of a monetary incentive (store vouchers) to promote fruit and vegetable purchasing in a remote Australian Aboriginal community did not yield significant increases in fruit or vegetable purchases [169]. Meanwhile, a weekly fruit and vegetable subsidy program, organized through an Australian Aboriginal medical service, was associated with improvements in some biomarkers of short-term health status among Aboriginal children [173,174]. Cost-effectiveness analyses have been conducted both by modelling theoretical discount/tax-induced changes in food purchases based on published price elasticity data and alongside intervention trials (e.g., SHOP@RIC) [175,176,177]. Modelled food pricing policies could improve diets and reduce mortality from diet-related diseases were estimated to be cost-effective, providing good ‘value-for-money’ [175,177]. By contrast, results from the intervention trial demonstrate that small and complex dietary changes leading to unintended health consequences (i.e., increases in sodium, energy and estimated change in BMI) can occur when adopting single focus price discount strategies and would not be deemed cost-effective [176].

#### 3.6.4. Other Initiatives

Other initiatives to improve the food purchasing environment include two studies involving the implementation of novel retail outlets—a mobile grocery to provide access to subsidized healthful foods [178], and a not-for-profit cooperative grocery store in a former food desert [179]. A significant proportion of shoppers at the latter were Indigenous people and used the store as their primary grocery store [179]. A small body of literature set in Australia and NZ (including intervention-trails [180,181] and epidemiological modelling studies [182,183], also examined environmental sodium reduction strategies (namely, through modifications in food processing) in both Indigenous and multiethnic community contexts. For example, an intervention trial in 26 remote Indigenous community stores in Australia demonstrated that a 25% salt reduction in a top-selling bread product did not affect sales; if implemented across all bread products, this could lead to significant health gains at the population-level [180]. In NZ, findings from the modelling (using a Markov macrosimulation model) of eight sodium reduction interventions using administrative cost data suggested that intervention strategies could achieve major health gains for Māori [182].

**Table 4:** Summary of intervention research ^1^ involving the retail sector ^2^ and Indigenous Peoples in HICs ^3^.

Intervention Name Goal/Type	Setting ^4^		Number of Intervention Stores/ Communities ^5^	Intervention ^6^				Process Evaluation	Evaluation and Impacts Examined ^6^			
	Country	Geography or Location		Availability	Affordability	Quality	Point of Purchase Promotion		Design	Psychosocial	Diet or Purchase	Health
**AHS: Apache Healthy Stores** [152,153] Food store-based obesity and chronic disease risk reduction program (dietary improvement)	US	White Mountain and San Carlos Reservation	11 (6)/2 reservations	✓			✓	Differed by level. At the store level: high level of dose and reach, and a moderate to high level of fidelity	Quasi-experimental design (pre-test/post-test longitudinal study)	✓	✓	
**CHILE: Child Health Initiative for Lifelong Eating and Exercise** [80,162,163,166] Multicomponent obesity prevention intervention for children in Head Start Centers	US	Rural	/6 American Indian sites	✓			✓	Participant engagement, recruitment and retention	Group randomized controlled trial			✓ BMI
**Healthy Communities Project** [168] Multicomponent pilot health promotion project (reduce sugary drink consumption and increase water consumption)	AU	Remote	/3	✓ Drink availability					Qualitative and quantitative evaluation	✓ - Community readiness - Awareness of social marketing messages	✓ Store drink sales (water vs. sugary drink)	
**HFH: Healthy Foods Hawaii** [161] Multicomponent obesity risk reduction and dietary improvement intervention for children (includes local producers/distributors)	US	Hawaii	5/2 (2)	✓			✓	High fidelity and moderate reach and dose reported	Pre/post-assessment of child-caregiver dyads in intervention and comparison communities	✓ Knowledge and the perception that healthy foods are convenient (caregiver)	✓ Healthy Eating Index (HEI) score (children)	
**HFN: Healthy Foods North** [154,155,156] Multicomponent chronic disease risk reduction and dietary improvement intervention (nutrition and physical activity)	CA	Arctic and northern	9 food stores + 3 convenience stores /4(2)	✓			✓		Pre/post-assessment in intervention and comparison communities	✓ Food related self-efficacy and intentions	✓ Unhealthy food acquisition frequency	✓ BMI
**Looma Healthy Lifestyle Program** [79] Community-directed healthy lifestyle program to reduce risk of chronic disease (reduce coronary heart disease through dietary modification)	AU	Remote	1/1	✓		✓ Appointment of community member as store manager to improve quality of the food supply			Trends in risk factors across the community after the start of the intervention were examined in 3 cross-sectional surveys		✓ Apparent diet (store turnover method)	✓ Plasma markers of coronary heart disease risk that are associated with diet
**NHSI: Healthy Navajo Stores Initiative** [125] Multifaceted intervention drawing from National “Healthy Corner Store” best practices (including a fruit and vegetable prescription/voucher program)	US	Navajo Nation (rural/remote)	Stores across Navajo Nation were invited to participate			✓ Staff training on produce handling to maintain freshness	✓	Food environment assessment (fruits and vegetable index score)	Multi-phase longitudinal study; Cross sectional survey of shoppers at participating compared to non-participating stores		✓ Fruit and vegetable purchasing	
**NHS: Navajo Healthy Stores** [157] Multicomponent food environment intervention to increase the availability of healthier foods and	US	Navajo Nation (rural/remote)	Total of 10 store regions across the Navajo Nation (5 immediate, 5 delayed)	✓			✓		Store-region randomized controlled intervention/ Pre-post differences by intervention group and by intervention exposure level	✓ Healthy food intentions	✓ Healthy food acquisition	✓ BMI
**OPREVENT: Obesity Prevention and Evaluation of InterVention Effectiveness in NaTive North Americans** [160] Multicomponent obesity (and related comorbidity) reduction initiative	US	Rural	25/total 5 (3 immediate, 2 delayed)	✓			✓	- Food store environmental checklist - Food store process form - Intervention exposure evaluation	Community randomized controlled trial	✓	✓ Dietary assessment	✓ Anthropometry
**SHOP: Supermarket Healthy Options** [112,136,164,172] Multicomponent (price discount and nutrition education) intervention (and pilot study) to promote healthier food purchasing	NZ	Urban	8/3 (pilot: 1/1)		✓ Price discounts (with and without nutrition education)				Factorial randomized controlled trial		✓ Individualized electronic shopping data (healthy food purchasing and percentage energy from saturated fat)	
**SHOP: Supermarket Healthy Options Project @RIC (Remote Indigenous Communities)** [81,176] Multicomponent (price discount and nutrition education) intervention to promote healthier food purchasing	NZ	Not specified	20/20		✓ Price discounts (with and without nutrition education)				A stepped wedge randomized controlled trial		✓ Weekly store sales data on all food and drinks sold	✓ Disability Adjusted Life Years (and cost effectiveness)
**THRIVE: Tribal Health and Resilience in Vulnerable Environments** [61,94] Initiative to improve the tribal food environment through interventions in tribally owned convenience stores	US	Rural	4 (4)/2 Nations	✓ Variety and convenience	✓ Reduced pricing		✓	. High fidelity across strategies in the intervention reported	Cluster-controlled trial design with treatment conditions at the store level; Mixed-effects linear regression pre- to postintervention changes	✓	✓ Purchasing andfruit and vegetable intake	
**ZATPD:Zhiiwapenewin Akino’maagewin: Teaching to Prevent Diabetes** [158,159] Multicomponent diabetes prevention (feasibility study) building on the Sandy Lake Health and Diabetes Program and others [184]	CA	Arctic and northern (remote and semi-remote)	/7 First Nations in 4 sites total (2 delayed intervention)	✓			✓	Moderate fidelity at the store-level	Quasi-experimental pretest/post-test evaluation between intervention and comparison communities	✓	✓ Healthy food acquisition	✓ Anthropometry

^1^ The table summarizes published studies related to the interventions, obtained and retained through this literature review. Other articles related to the initiative (e.g., trial registration, formative research) may exist in the literature. ^2^ The table reports on the store-based, and participant, component of multi-sector interventions (i.e., intervention aspects related to other community food environment settings) are not reported here. ^3^ Global economies are commonly categorized by country across four income groupings: low, lower-middle, upper-middle, and high [185]. A country with a high-income economy is defined by the World Bank as a country with a gross national income per capita of USD 12,535 or more in 2019. Income is measured using gross national income (GNI) per capita, in U.S. dollars, converted from local currency using the World Bank Atlas method. ^4^ Countries: AU = Australia; CA = Canada; NZ = New Zealand; US = United States; Geographic setting as self-defined in the article. If geographic setting was not stated in the study, the location of the intervention is included. ^5^ Number of comparison/control communities and/or stores is indicated in brackets. ^6^ Checkmark indicates inclusion of element in study intervention or evaluation. Intervention outcome results are not reported in the table.

## 4. Discussion

Indigenous Peoples consist globally of numerous and diverse communities with distinct and complex structures and histories of self-governance, dispossession, independence, and recognition vis a vis settler states. As ascertained through results of both empirical and respondent-based approaches, this review identifies several common retail food sector issues across Indigenous regions (and/or regions with a high prepotency of Indigenous Peoples) in HICs. Importantly, this review also identifies significant geographic, populational, methodological, conceptual, and temporal research gaps in knowledge (see Section 4.4). Thus, interpretation of this discussion must be situated within the constraints of these deficits in the state of available scholarly evidence, as well as the study limitations (Section 4.4).

### 4.1. Retail/Consumer Food Environment/Supply Issues

At the food supply level, several common challenges are noted across regions—notably, the high cost (in some contexts, several times higher than referent locations), poor quality (e.g., damaged and/or close to or exceeding their expiry date after arrival), and limited availability and choices of healthful foods, compounded by the ready accessibility of less healthful and processed options. Relatedly, at the store-level, retail food environments across these settings diverge in many respects, from general conditions prevalent in HICs [29,37,186,187] but share features that mirror aspects of both ‘‘food deserts’’ (e.g., absence of an accessible supermarket) and “food swamps” (e.g., increased exposure to convenience stores and stores replete with discretionary food items) [188]. Moreover, although this review did not focus on other community food environments, Indigenous Peoples may also experience heightened exposure to other “unhealthy” food outlets, such as fast-food restaurants [69]. Limited availability of supermarkets and grocery stores, compounded by transportation related barriers to their access, and high exposure and dependence on, small and nontraditional food retailers—marked issues in rural and remote areas, although they also present in urban centers [189]—is highlighted in several studies across global regions [190]. In this review, associations between store type and food supply, and/or community health are documented in several studies (Table 2). Less considered, however, is the impact of other vendor characteristics such as store governance, industry codes of practice and marketing, and private/public interests in shaping store operations, policies, and the resultant food supply [191,192]. Market forces (which drive factors such as store locations, supply–demand dynamics, buying power) and private sector structures may constrain the options available to local and Indigenous governments seeking to self-determine the quality and characteristics of their food environments. Although store managers can positively influence the nutritional quality of the food supply in remote communities [193], local store managers may not be able to set prices locally and may be beholden to corporate policies and retailing practices.

By severely constraining the available opportunities for healthful diets the retail food sector may play a strong role in shaping dietary patterns and health—particularly in rural and remote regions where individuals/households lack access to culturally valued traditional/subsistence foods. Constraints in local food supplies are perceived by community members to represent major barriers to food security, healthy food purchasing/eating, and health [57,89,116,123,145,194]. Although food deserts and food swamps have been associated with increased consumption of less healthful foods (e.g., snacks/desserts), and obesity [195,196] in the literature, most studies have been set in urban, low-income areas, and have involved non-Indigenous minority populations [197]. Studies relating the retail food environment/supply to consumer health and health-related behaviors and psychosocial factors, among Indigenous Peoples in HICs are few. Nevertheless, available evidence finds associations with obesity [84,87] and food security status [85]. Furthermore, constraints in the retail food sector are mirrored by the significant burdens of poor dietary quality, obesity and chronic disease, and food insecurity, documented in cross-sectional population health surveys—see for example [21,198,199].

### 4.2. Common Structural Issues Across Regions

There exists today a wide diversity in the degree to which Indigenous communities exercise self-governance over their land and territory, including the local food supply. Amid ongoing struggles for Indigenous self-determination, several common factors persist across regions to structure contemporary food environments. In particular, geography (rurality and remoteness [200]), supply-chain logistics, inequitable market exchange, and settler colonialism, may create particular challenges for contemporary retail food environments from a public health perspective. For example, studies examining food supply in relation to indices of geographic remoteness find that foods typically cost more, and the availability and quality of fresh produce is lower with increasing geographic remoteness, as well as in the absence of all-weather roads [92,111,201]. Food retail systems in several regions involve high dependence on distal food imports [64] and challenging supply chain logistics (e.g., transportation, infrastructure, cost) [56,57,58,60,111]. While these issues are recognized to affect retailing operations across remote Indigenous communities—from the Arctic to Australia, including the potential for similar dynamics in the Pacific Islands—their impact on food supply and community health remains limitedly examined in the literature. Despite successful examples in the literatures of alternative food supply streams (e.g., direct exchanges between northern fishers and southern farmers [148], bulk ordering for community [70,142], and sourcing food from local producers [161,202], there are considerable challenges to practical implementation. Furthermore, there may be community concern that such initiatives undermine more locally controlled food systems [148].

While the locality and cultural relevance of the food supply have seldom been assessed in quantitative and empirical research (see [119] for a notable exception), such topics feature in several qualitative studies involving community perspectives (Table 3). Indeed, qualitative studies underscore the need to interrogate and address historical underpinnings of contemporary food system issues. Settler colonialism, in particular, has been conceptualized as a form of domination specifically targeting Indigenous food sovereignty, by undermining the reciprocal relationships that uphold Indigenous self-determination and “collective continuance” [203]. For decades, Indigenous Peoples have actively resisted the adverse impacts of colonialism, while emphasizing the right to self-directed cultural change and economic development [204,205,206]. Complex socioeconomic dynamics characterize food systems in contemporary Indigenous communities, including the maintenance of Indigenous values, kinship ties/relationships, and cultural norms when integrating new inputs (e.g., market-based/cash economies) [207,208]. Although in many HICs, Indigenous Peoples exert varying forms of self-governance over defined regions, territories, or reservations, in many cases, Indigenous communities have neither consented to, nor benefited equitably from, developments on their ancestral territories that have served to enrich contemporary settler colonial nation-states, while compromising local food systems (e.g., contamination, wildlife decline) [209,210,211]. Furthermore, as stated previously, some stores in Indigenous regions retain direct and indirect lineages to colonial enterprises. There is limited examination in the public health literature of how such histories and their enduring legacies have shaped contemporary food supplies, shopping experiences, and consumer demand among Indigenous Peoples, although these themes feature strongly in the work of Indigenous scholars and in other humanities scholarship–see for example [203,212,213].

### 4.3. Towards Equitable Food Systems

A spectrum of environmental interventions, policies, and priorities involving the retail sector—including actively stocking healthful low-cost food items, point-of-purchase promotions (e.g., nutrition labelling), economic tools (e.g., subsidies and taxes), and supply chain interventions—have, in conjunction with approaches targeting behavioral and psychosocial dimensions (e.g., information campaigns, nutrition advice and counselling, education/skills development), been reported in the literature for these contexts. In the general literature, store-based nutrition environment interventions are identified as having less impact on point-of-purchase behavior than those set within other community food environments (e.g., worksites and schools) [214]. In this review, several multisector strategy/interventions involving store-based components have been associated with improvements in a range of psychosocial, behavioral, and nutrition-related health outcomes among Indigenous People (Table 4). This divergence from general patterns in the literature may underscore the pronounced influence of the retail food environment in shaping community diets in these contexts. Although a few intervention studies (set in Australia and NZ) suggest that food pricing policies can improve population health and reduce inequalities [81,170,177,215] the effectiveness of national subsidies such as the Nutrition North Canada program, remains largely unassessed [74,216]. More fundamentally, supporting healthful and sustainable food systems for Indigenous Peoples in HICs requires efforts predicated upon Indigenous (food and political) sovereignty and self-determination, human rights (e.g., the recognition of food as a human right, and stores in remote communities as essential services), and (health and social) equity.

For example, FN community members have suggested that improvements in the local food environment may lie outside the model of capitalist exchange, which is perceived as a significant barrier to affordable food [217,218]. With the understanding that the way remote community stores operate and the quality of food they provide plays a strong role in shaping the diet and health of Indigenous people [78], the provision of basic healthy food is considered to be a governmental responsibility in Greenland. Meanwhile, remote community stores in Australia are community-owned and operate as an “essential service”, rather than as privately held businesses [219,220]. These stores generally fund long-term policies, such as fruit and vegetables discounts, although more recently implemented policies have partly been funded by suppliers and manufacturers [151]. In Canada and the United States, a more complex pattern of subsidized market mechanisms and government food/social support exist [221]. In northern Canada and rural AI communities, where supermarkets are rare, locally owned and operated co-operatives and convenience stores are an important food source [222]. Community ownership of retail food stores provides an opportunity to uniquely influence the food supply and implement interventions/policies to support public health, in a manner that is predicated upon community values [223,224]. Yet, limited resources have been allocated to build capacity in a local community-based workforce dedicated to nutrition and food security in the retail and other food-related sectors in Indigenous community contexts.

Indigenous peoples globally are involved in efforts to decolonize diets [225,226] and affirm the importance of Indigenous food systems and food sovereignty for various facets of health and wellbeing [203,205,206,212,213,227,228,229]. Food sovereignty efforts recognize that food is not simply a market commodity, but also, a politically embedded process [228]. Accordingly, Indigenous movements for food sovereignty undergird struggles for broader political sovereignty, Indigenous resistance and resurgence [212], as well as reconciliation, [148,228]. Although traditional food systems remain fundamental to Indigenous food sovereignty, respondents in the few available studies to examine the issue, have expressed a preference for diets that involve both traditional and market foods [230,231,232]. Thus, improvements in the retail food sector may play a role in supporting enabling conditions for the sustained and improved health of Indigenous Peoples in HICs and initiatives to foster community food security and community health must consider overlapping social, economic, and environmental factors and the complex interplay between subsistence and market-based food systems. Thus, research and policy aimed at improving the retail food sector requires population-specific modes of reference and conceptualization as well as approaches and tools, distinct from Western approaches, and predicated upon Indigenous knowledge, priorities and worldviews, and respectful of Indigenous rights [43,44]. This includes, but is not limited to, integrating issues of accommodation (i.e., the capacity of the food sector to accommodate and adapt to local needs) within the retail food sector [53].

### 4.4. Research Gaps, Methodological Considerations and Future Research

The findings from this review illustrate temporal, geographic, and topical gaps and areas of concentration in the published literature to date (Table 5). Despite widespread concern about the lack of healthful food availability and high food prices in Indigenous communities across several HICs, research about the retail food sector remains limited. Furthermore, although rural and remote communities are an important priority for health equity, there are also important gaps in urban and semiurban settings. These results highlight the need to better understand the impact of the retail food sector/environment on dietary choice and health, as well as in driving dietary change among Indigenous Peoples [233]. They underscore, furthermore, the need to develop methods and tools to assess and better account for disparities in food environments within, and across, HICs. Building upon the knowledge gaps identified, we propose several key recommendations for future research, monitoring, and interventions, involving the retail food sector and Indigenous Peoples in HICs.

Ultimately, fostering learning across contexts and among different actors and stakeholders in the food sector is needed, and may benefit from a global community of practice driven by Indigenous-identified priorities, and centering Indigenous scholarship and scholars. Such an approach must be predicated upon “relationships, reciprocity and self-determination” and advocating for decolonizing research methodologies [44], sharing best practices, resources, and creating new knowledge to advance evidence-based policy and practices [78].

### 4.5. Limitations

Several limitations of this study should be acknowledged. First, only peer-reviewed academic literature was reviewed; gray literature was excluded. While reports from Indigenous organizations outside academia may have been captured with a search which included the latter (see for example, [235,236]), the goal of this review was first to synthesize studies and findings in the published academic literature. Relatedly, the literature search strategy was developed in English, and results were limited to English-language articles. Thus, literature that was not indexed in English was excluded. The exclusion of papers for linguistic reasons can yield different study conclusions [237]. Second, although the search terms were global in scope and were based on a systematic method to identify Indigenous Peoples, we neither defined, nor identified, any additional populations/groups. Thus, Indigenous Peoples included in the search strategy was based exclusively on the prior identification of such populations in the databases and documents searched. Although we used general and specific search terms for Indigenous population in the literature review, it is not feasible to comprehensively include terminology specific to all Indigenous groups. In the U.S., for example, there are over 500 federally recognized tribal entities. Furthermore, given the geographic scope, only two major academic databases were systematically searched. Thus, the search was not exhaustive and other relevant articles may not have been identified. Third, this review was restricted to Indigenous Peoples in HICs, recognizing that Indigenous Peoples in middle and upper-middle income countries (e.g., Mexico, Brazil, Russia) may experience similar retail food sector issues and challenges. Similarly, other minority groups in HICs may share in experiences of having distinct communities, languages, cultures, beliefs, social, economic, and political systems than the dominant society that could affect food security, health, and nutrition, through their experience and interactions with the retail food sector [3]. There is still much to be learned about how to improve the food environment for sociodemographic groups with above-average obesity risks [238]. Moreover, definitions of HIC are dynamic, as they may change with country economic status through time. Fourth, this review focused exclusively on the retail food sector (i.e., stores that sell food from the local and global agri-food sector), which does not explicitly include other types of markets, such as farmer’s markets. This definition also excluded formal or informal country food markets such as those in Greenland or on social media advertising/selling food within, or between, communities [239,240]. Results from this review underscore the need to consider interacting dynamics among agricultural and wildlife harvesting activities with local and regional markets, and the respective involvement and governance of Indigenous Peoples and public governments in relevant policies and activities. They underscore furthermore the need to consider other forms of food provision (e.g., food supplied through government aid) and sourcing (e.g., food ordered over the internet), which circumvent local retail food stores. Fifth, this review did not incorporate a gender-based lens/analysis. Gender dynamics are also important to understand, as Indigenous women may play a greater role than men in food purchasing [126]. Sixth, based on the objectives and scope of this review (i.e., to synthesize knowledge to inform future research, as opposed to answering a specific question), and as is customary with scoping review methodologies, articles were not assessed for methodological or data quality [51]. Nevertheless, not all studies involved statistical analyses, and intervention studies may not have adjusted results for relevant covariates, which can significantly impact study conclusions [197]. Although we categorized articles based on the involvement of Indigenous Peoples in the research, we did so broadly, and did not distinguish based on the level of engagement and participation. Indicators exist for systematically appraising and scaling levels of Indigenous community engagement in research [241]. Their incorporation in future knowledge syntheses and original research studies may support a foundation for more reflexive and responsible research practice with Indigenous communities. Finally, this review employed an inductive and deductive thematic classification scheme, and accordingly, the major themes presented may have been biased towards those reported in existing conceptual frameworks and literature derived principally from non-Indigenous contexts. There is a need for Indigenous-specific frameworks of local food systems (see for example [227,242,243]) to inform future research and knowledge syntheses on this topic.

## 5. Conclusions

Food environments that support equitable access to healthy diets (with varying contributions of market, and traditional foods—based on local preferences and other factors) are requisite to overcoming the systemic disparities in diet quality, food security, and diet-related health conditions experienced by Indigenous Peoples, especially in remote community contexts [8]. Efforts to support Indigenous population health in HICs must emphasize the urgent need for adequate food environments and recognize underlying structural challenges. Unless inadequacies and inequities in the market food sector are addressed, expensive poor quality food supplies, and food globalization, will continue to constrain access to healthy diets for Indigenous Peoples in HICs [244]. This review provides a baseline for future syntheses and may help movement towards more global collaborations on these matters. It emphasizes the need for a greater understanding of the key structural factors contributing to food supply issues (namely, interacting logistic, economic, governance, and policy factors) to inform the development of effective policies and interventions, as well as barriers to, and opportunities for, building more healthy, affordable, and culturally appropriate food systems. Results from the literature also confirm that food supply monitoring can be used strategically to advocate, lobby, and build local capacity towards health equity. Nevertheless, research on these issues must, be sensitive to enduring disparities in power and participation of Indigenous Peoples in matters that concern their local food systems (despite considerable advances in Indigenous self-governance in some regions). More work is also needed to better understand factors contributing to inadequacies in food supply and their multidimensional impacts on health and wellbeing in order to inform effective policy development at multiple scales.

Food stores in Indigenous communities, and indeed the retail food sector more generally, have the potential to play a more positive and supportive role in health promotion. There is a need to understand how profit- and health-driven models of food retailing can mutually satisfy health, socio-cultural, and economic objectives. There also remain important questions regarding the opportunities and constraints for communities to influence their own retail food system and thereby exercise food sovereignty in the retail sector. Local decision-making is a key dimension of enabling community members to shape the qualities and characteristics of their local food environment and critical for food system improvement. Improving the retail food sector necessitates horizontal and vertical collaborative actions involving coordination with traditional food systems, between Indigenous and other governments, the private sector, and other actors at various scales. Factors such as community ownership are central to culturally appropriate policy and program development and implementation and may also support Indigenous self-determination. This also will ultimately require efforts predicated upon Indigenous cultural, economic and political resurgence, dietary decolonization, and Indigenous food sovereignty. Of course, focusing exclusively on the retail sector could not be sufficient on its own to heal the continued disruptions to Indigenous ecologies and foodways, driven by the histories of colonial expansion, the persistence of neo-colonial architecture in the nation-state, and the persistent and accelerating economic inequalities produced and upheld by the capitalist world system and with its logics of exchange and accumulation. However, closing the gap on all forms of inequalities, income, structural, or otherwise, may yet contribute to overall improvements in population health.

## Figures and Tables

**Table 1:** Summary of studies examining food store availability and access.

Reference	Setting ^1^		Methods		Outcomes Examined	Conclusion	Relationship to Food Supply and/or Health
	Country	Geography	Empirical	Respondent-Based			
[66]	CA	Arctic and northern	Mapping	Survey of retail experience	- Retail competition (presence of a second retailer) - Retail and shopping experience	There is limited retail competition in most communities which lack year-round road access	Respondents expressed concerns regarding food supply (availability, cost, quality and freshness)
[65]	USA	Not specified	Mapping	Interviews (Tribal members)	- Distance to, and density of, retail outlets (healthy vs. unhealthy) in relation to tribal area - Perceptions and experiences of the food environment	Lower density of healthy food outlets in tribal areas compared to nontribal areas	Respondents perceived food environment negatively and ported barriers to the acquisition of healthy food
[67]	CA	Rural		Asset mapping (youth)	- Places where youth acquire food (how they are perceived, and how to improve them for healthy living)	Gas station and convenience store were the only place to purchase groceries in the community	Available food was perceived to be of poor quality and recommended that healthier food be sold
[68]	USA	Rural; Urban	Inventorying (secondary data; ground-truthing / site visitation)		- Enumerating food outlets and match rate for secondary data and on-site observations for different types of food outlets	Secondary data sources both over- and under- estimate the food environment especially for nontraditional retailers	
[71]	USA	Rural; Remote	Inventorying (secondary data; telephone survey)		- Vendor characteristics (type, ownership) - Food supply assessment (availability and price)	General characteristics of available stores (on Navajo Nation and Border Towns) are reported	Navajo convenience stores offered fewer healthier food options compared to Navajo supermarket
[72]	Guam	Island	On site observations		- Map of food stores (by type) in relation to participants - Association to food supply, diet, and health	The majority of stores within a mile from participants were small markets	Living near a small market was negatively correlated with body mass index (BMI); while living near a convenience store was positively correlated
[73]	USA	Not specified	Inventorying (secondary data; telephone survey; site visitation)		- Type number and location of food stores - Availability and cost of food	Half of stores identified on 22 American Indian reservations were convenience stores. A total of 17 reservations did not have a supermarket on their reservation, and the nearest off-reservation supermarket was 10 miles from the tribe’s headquarters	Across all stores, about 38% of checklist items were available, with foods from the dairy and sugars/sweets groups being the most available, while fresh fruits/vegetables being the least available. Cost of the most commonly available items was lowest in supermarkets
[69]	CA	Urban	Mapping (census and store location data)		- Supermarket within 800 meters by percentage of Indigenous residents (and other socio-economic/demographic info)	Supermarket exposure did not differ in neighborhoods with a higher percentage of Indigenous residents	
[70]	CA	General/National	Internet search		- Alternative food procurement locations (Indigenous food co-ops)	In total, 42 Indigenous food co-ops were identified (notably in northern Canada)	

^1^ Countries: CA = Canada; GU = Guam; USA = United States; Geography as self-defined in the study (except for Island states). ‘Arctic and northern’ includes the provincial North of Canada.

**Table 5:** Summary of research gaps and challenges and proposed research needs and/or future directions.

Dimension	Research Gaps and/or Challenge	Proposed Research Needs and/or Future Directions
Country	- Most research was set in Australia, the United States, Canada and New Zealand; few studies were available from all other HICs	- There is a need for studies examining the retail food sector among Indigenous Peoples in several HICs (e.g., French Polynesia, Panama, etc.) - Through international collaboration, there is potential for developing and implementing comprehensive, adapted, and standardized tools, metrics, and frameworks for assessing and monitoring the retail food sector (and the impact of interventions therein) at local, national, and international levels
Geographic and populational setting	- Most research and interventions focus on issues within a geographically defined community—mostly in rural, remote, and northern regions - Research involving Indigenous people in multi-ethnic and urban areas is limited and available research has highlighted the challenges of generating a valid evidence base by ethnic/social groups—notably challenges related to the recruitment and retention of under-represented populations	- Indigenous Peoples living outside of tribal/reservation lands, as well as landless tribes and Métis communities, comprise an important fraction of the Indigenous population in several HIC settings, and more research is needed to understand their experiences and interactions with the retail food sector
Temporal	- Most studies were observational, involving cross-sectional designs and/or were conducted during a single time point, making it impossible to ascertain differences across seasons and/or over time	- Seasonal and longitudinal frameworks for assessing and monitoring the retail food sector and interventions over time and across contexts are needed
Research approach and study design	- While most studies involved some level of community engagement and participation, we did not systematically appraise or characterize Indigenous involvement in research in this review - There are few mixed and multi-method studies that include both empirical observations of the retail sector and community knowledge and perceptions thereof - While community conceptualizations of food sector issues are often very broad (and include multiple overlapping dimensions and various scales), empirical assessments conducted in these settings are typically focused/ narrow (e.g., don’t take into consideration cultural context and preferences)	- Enhance reporting structures (as well as journal expectations for reporting structures) for Indigenous engagement in food-related research. Ideally, research should meaningfully support community capacity in research and the development of evidence-based policies that responds to local priorities - Use qualitative and mixed/multimethod study designs (with a focus on Indigenous and decolonizing methodologies) to better capture complex community food issues. These study designs can play a key role in bridging the gap between local priorities/needs, nutrition and health assessments, food environment measures, and policies/interventions
Research tools	- Most studies involved standardized or adapted tools (few studies developed research instruments expressly for the local context). Although there is a wide range of food environment assessment instruments reported in the general literature, conventional methodologies can be ill-suited to Indigenous community settings, and the degree to which these measures are appropriate and relevant to Indigenous community contexts remains under-assessed	- There is a need for research instruments that are culturally-relevant, as well as valid and reliable for these contexts—in particular for small and nontraditional retailers and rural and remote contexts where secondary data sources both over- and under-represent such community food environment
Domains of the food sector assessed	- Few studies have included multiple dimensions of the food supply, such as both food cost and quality, within their assessments and or included mixed-methods or multiple data sources (e.g., store audits, point-of-sale data, as well and dietary and health data)	- Comprehensive assessments of multiple dimensions of the food supply (e.g., cost, availability), including how these interact with consumer factors (e.g., affordability, preferences, purchase), are needed. For example, the affordability of a theoretical healthy diet compared to the current diet (particularly when combined with detailed income and expense information) can be used for social policy planning and to advocate for fiscal policies (e.g., subsidies) - Data derived from multiple sources (e.g., till receipts and store sales data and dietary information), particularly when representing different dimensions/levels of the food sector, can be highly complementary in developing food and nutrition policies and initiatives, and in monitoring changes in the food environment over time
Food items included	- Assessments of food environments often consider exclusively the presence of healthful items (based on checklists focusing on fruits, vegetables and other fresh and healthful items). Items included in food supply assessments are not necessarily representative of the complete store inventory, nor are they necessarily representative of community food purchasing patterns. Pricing surveys, for example, have often reported on food basket (or adapted food basket) costs, which include a limited subset of available foods, typically exclude discretionary items, and are not representative of food consumption or expenditure patterns. For example, although ultra-processed foods are prevalent in Indigenous Peoples diets [234], these have been limitedly examined in the retail environment literature	- Utilize a more holistic list of available items, including those highly consumed (e.g., discretionary energy-dense foods, prepared/convenience, processed foods), those which represent a good nutritional value for price, and those which are otherwise valued by community members (e.g., branded vs. generic products, foods that can be taken “on-the-land” during harvesting activities) - Other metrics of food availability, such as the amount of retailer shelf-space allocated to healthful vs. discretionary food items, should also be assessed
Contextual factors	- Few studies include contextual factors or drivers influencing the local food environment and supply (e.g., geography, logistics, store operation and management practices)	- Studies should include the impact of circumstantial constraints (e.g., seasonal flooding/freezing and other weather-related dynamics in food shipping), and other social and economic dynamics (e.g., income support cycles) on the food supply (both market and traditional foods and their interactions), purchasing (e.g., compensatory purchasing), food security, and diet quality - As the logistics of food retailing will be affected by climate change (e.g., shipping seasons) and related investments (e.g., new harbors in the Arctic), climate change should be included as an important determinant of local food supplies, and, ultimately, health, within research, policies and adaptation planning, involving the retail, and other, related sectors
Equity informed methods	- Few studies explicitly assess disparities in the food environment/supply (e.g., between Indigenous communities and other settings). There exist few (objective and subjective) measures that capture the opportunities and constraints to accessing food and health in these settings	- Respond to the call to apply a health equity lens to population-level food environment policies [41] and bring health and social equity issues to the forefront of retail food sector research. Such assessments may also be used to compare food environments across settings and over time, to evaluate policy (e.g., compliance with Indigenous and government food policies, guidelines, or voluntary codes of practice) and assess the impact of retail food environments on health outcomes

HICs = High income countries.

## Supplementary Materials

The following are available online at https://www.mdpi.com/1660-4601/17/23/8818/s1, Figure S1: Process of identifying Indigenous Peoples in high income countries, Table S1: Identification of Indigenous Peoples in high income countries, Table S2: PRISMA-ScR Checklist, Table S3: Literature search themes and combinations of search terms, Table S4: Literature search strategy and results, Table S5: Eligibility criteria for screening, Table S6: Data charting scheme, Table S7: Key characteristics of the articles included in the review.

## References

[1] World Bank Indigenous Peoples Overview. https://www.worldbank.org/en/topic/indigenouspeoples.

[2] Kuhnlein H.V., Erasmus B., Spigelski D. (2009). Indigenous Peoples’ Food Systems: The Many Dimensions of Culture, Diversity and Environment for Nutrition and Health.

[3] UNPFII Indigenous Peoples, Indigenous Voices Fact Sheet. Who are Indigenous Peoples?. https://www.un.org/esa/socdev/unpfii/documents/5session_factsheet1.pdf.

[4] APF, OHCHR (2013). The United Nations Declaration on the Rights of Indigenous Peoples: A Manual for National Human Rights Institutions.

[5] Osborn D., Cutter A., Ullah F. Universal Sustainable Development Goals: Understanding the Transformational Challenge for Developed Countries. https://sustainabledevelopment.un.org/content/documents/1684SF_-_SDG_Universality_Report_-_May_2015.pdf.

[6] Reading C.L., Wien F. (2009). Health Inequalities and the Social Determinants of Aboriginal Peoples’ Health.

[7] Vos T., Barker B., Begg S., Stanley L., Lopez A.D. (2009). Burden of disease and injury in Aboriginal and Torres Strait Islander Peoples: The Indigenous health gap. Int. J. Epidemiol..

[8] Browne J., Hayes R., Gleeson D. (2014). Aboriginal health policy: Is nutrition the ‘gap’ in ‘Closing the Gap’?. Aust. N. Zeal. J. Public Health.

[9] Council of Canadian Academies (2014). Aboriginal Food Security in Northern Canada: An Assessment of the State of Knowledge.

[10] Jernigan V.B.B., Huyser K.R., Valdes J., Simonds V.W. (2016). Food Insecurity Among American Indians and Alaska Natives: A National Profile Using the Current Population Survey–Food Security Supplement. J. Hunger. Environ. Nutr..

[11] Paradies Y. (2016). Colonisation, racism and indigenous health. J. Popul. Res..

[12] Albala C., Vio F., Kain J., Uauy R. (2002). Nutrition transition in Chile: Determinants and consequences. Public Health Nutr..

[13] Lourenço A.E.P., Santos R.V., Orellana J.D.Y., Coimbra C.E.A. (2008). Nutrition transition in Amazonia: Obesity and socioeconomic change in the Suruí Indians from Brazil. Am. J. Hum. Biol..

[14] Hughes R.G., Lawrence M. (2005). Globalisation, food and health in Pacific Island countries. Asia Pac. J. Clin. Nutr..

[15] Whiting S.J., MacKenzie M.L. (1998). Assessing the changing diet of indigenous peoples. Nutr. Rev..

[16] Kenny T.-A., Hu X.F., Kuhnlein H.V., Wesche S., Chan H.M. (2018). Dietary sources of energy and nutrients in the contemporary diet of Inuit adults: Results from the 2007–08 Inuit Health Survey. Public Health Nutr..

[17] Ferguson M., Brown C., Georga C., Miles E., Wilson A., Brimblecombe J. (2017). Traditional food availability and consumption in remote Aboriginal communities in the Northern Territory, Australia. Aust. N. Zeal. J. Public Health.

[18] Neitzel A.L., Smalls B.L., Walker R.J., Dawson A.Z., Campbell J.A., Egede L.E. (2019). Examination of dietary habits among the indigenous Kuna Indians of Panama. Nutr. J..

[19] Brimblecombe J., Ferguson M., Liberato S.C., O’Dea K., Riley M. (2013). Optimisation Modelling to Assess Cost of Dietary Improvement in Remote Aboriginal Australia. PLoS ONE.

[20] Rosol R., Huet C., Wood M., Lennie C., Osborne G., Egeland G.M. (2011). Prevalence of affirmative responses to questions of food insecurity: International Polar Year Inuit Health Survey, 2007–2008. Int. J. Circumpolar Health.

[21] Huet C., Rosol R., Egeland G.M. (2012). The Prevalence of Food Insecurity Is High and the Diet Quality Poor in Inuit Communities. J. Nutr..

[22] Pollard C.M., Nyaradi A., Lester M., Sauer K. (2014). Understanding food security issues in remote Western Australian Indigenous communities. Health Promot. J. Aust..

[23] Brimblecombe J., Ferguson M.M., Liberato S.C., O’Dea K. (2013). Characteristics of the community-level diet of Aboriginal people in remote northern Australia. Med. J. Aust..

[24] Kuhnlein H.V., Receveur O., Soueida R., Berti P.R. (2007). Unique patterns of dietary adequacy in three cultures of Canadian Arctic indigenous peoples. Public Health Nutr..

[25] Pakseresht M., Lang R., Rittmueller S., Roache C., Sheehy T., Batal M., Corriveau A., Sharma S. (2014). Food expenditure patterns in the Canadian Arctic show cause for concern for obesity and chronic disease. Int. J. Behav. Nutr. Phys. Act..

[26] Kenny T.-A., Fillion M., MacLean J., Wesche S.D., Chan H.M. (2018). Calories are cheap, nutrients are expensive—The challenge of healthy living in Arctic communities. Food Policy.

[27] CIA (2020). The World Factbook.

[28] The Northwest Company Our Community Promise. https://www.northwest.ca/about-us/company-profile.

[29] Caspi C.E., Sorensen G., Subramanian S., Kawachi I. (2012). The local food environment and diet: A systematic review. Health Place.

[30] Morland K.B., Evenson K.R. (2009). Obesity prevalence and the local food environment. Health Place.

[31] Caspi C.E., Lenk K., Pelletier J.E., Barnes T.L., Harnack L., Erickson D.J., Laska M.N. (2017). Association between store food environment and customer purchases in small grocery stores, gas-marts, pharmacies and dollar stores. Int. J. Behav. Nutr. Phys. Act..

[32] Freedman D.A. (2009). Local Food Environments: They’re All Stocked Differently. Am. J. Community Psychol..

[33] Drewnowski A. (2017). Nutrient density: Addressing the challenge of obesity. Br. J. Nutr..

[34] Drewnowski A., Darmon N. (2005). The economics of obesity: Dietary energy density and energy cost. Am. J. Clin. Nutr..

[35] Drewnowski A., Specter S.E. (2004). Poverty and obesity: The role of energy density and energy costs. Am. J. Clin. Nutr..

[36] Pulker C.E., Thornton L.E., Trapp G. (2018). What is known about consumer nutrition environments in Australia? A scoping review of the literature. Obes. Sci. Pr..

[37] Ni Mhurchu C., Vandevijvere S., Waterlander W., Thornton L.E., Kelly B., Cameron A.J., Snowdon W., Swinburn B., INFORMAS (2013). Monitoring the availability of healthy and unhealthy foods and non-alcoholic beverages in community and consumer retail food environments globally. Obes. Rev..

[38] Turner C., Kalamatianou S., Drewnowski A., Kulkarni B., Kinra S., Kadiyala S. (2019). Food Environment Research in Low- and Middle-Income Countries: A Systematic Scoping Review. Adv. Nutr..

[39] Turner C., Aggarwal A., Walls H., Herforth A., Drewnowski A., Coates J., Kalamatianou S., Kadiyala S. (2018). Concepts and critical perspectives for food environment research: A global framework with implications for action in low- and middle-income countries. Glob. Food Secur..

[40] Olstad D.L., Campbell N.R., Raine K.D. (2019). Diet quality in Canada: Policy solutions for equity. Can. Med. Assoc. J..

[41] Lana V., Guest E., Dana L.O., Vanderlee L., Olstad D.L. (2018). Commentary—Food environment and vulnerable populations: Challenges and opportunities for policy. Health Promot. Chronic Dis. Prev. Can..

[42] Pollard C.M., Begley A., Landrigan T.J. (2015). The Rise of Food Inequality in Australia. Food Poverty Insecurity Int. Food Inequal..

[43] HLPE (2017). Nutrition and Food Systems.

[44] Luongo G., Skinner K., Phillipps B., Yu Z., Martin D.H., Mah C.L. (2020). The Retail Food Environment, Store Foods, and Diet and Health among Indigenous Populations: A Scoping Review. Curr. Obes. Rep..

[45] Browne J., Lock M., Walker T., Egan M., Backholer K. (2020). Effects of food policy actions on Indigenous Peoples’ nutrition-related outcomes: A systematic review. BMJ Glob. Health.

[46] Cisneros-Montemayor A.M., Pauly D., Weatherdon L.V., Ota Y. (2016). A Global Estimate of Seafood Consumption by Coastal Indigenous Peoples. PLoS ONE.

[47] Litvinoff M.P.T., Anderson B. World Directory of Minorities and Indigenous Peoples. https://www.emerald.com/insight/content/doi/10.1108/RR-12-2015-0298/full/html.

[48] eHRAF World Cultures. https://ehrafworldcultures.yale.edu/ehrafe/.

[49] Anderson I., Robson B., Connolly M., Al-Yaman F., Bjertness E., King A., Tynan M., Madden R., Bang A., Coimbra C.E.A. (2016). Indigenous and tribal peoples’ health (The Lancet–Lowitja Institute Global Collaboration): A population study. Lancet.

[50] Grant M.J., Booth A. (2009). A typology of reviews: An analysis of 14 review types and associated methodologies. Health Inf. Libr. J..

[51] Peters M.D., Godfrey C.M., Khalil H., McInerney P., Parker D., Soares C.B. (2015). Guidance for conducting systematic scoping reviews. Int. J. Evid. Based Health.

[52] Shamseer L., Moher D., Clarke M., Ghersi D., Liberati A., Petticrew M., Shekelle P., Stewart L.A. (2015). Preferred reporting items for systematic review and meta-analysis protocols (PRISMA-P) 2015: Elaboration and explanation. BMJ.

[53] Penchansky R., Thomas J.W. (1981). The concept of access: Definition and relationship to consumer satisfaction. Med. Care.

[54] Glanz K., Sallis J.F., Saelens B.E., Frank L.D. (2005). Healthy Nutrition Environments: Concepts and Measures. Am. J. Health Promot..

[55] Goldhar C., Ford J.D., Berrang-Ford L. (2012). Prevalence of food insecurity in a Greenlandic community and the importance of social, economic and environmental stressors. Int. J. Circumpolar Health.

[56] Watson Z.A., Shanks C.B., Miles M.P., Rink E. (2018). The Grocery Store Food Environment in Northern Greenland and Its Implications for the Health of Reproductive Age Women. J. Community Health.

[57] Mead E., Gittelsohn J., Kratzmann M., Roache C., Sharma S. (2010). Impact of the changing food environment on dietary practices of an Inuit population in Arctic Canada. J. Hum. Nutr. Diet..

[58] Piltch E.M., Shin S.S., Houser R.F., Griffin T. (2020). The complexities of selling fruits and vegetables in remote Navajo Nation retail outlets: Perspectives from owners and managers of small stores. Public Health Nutr..

[59] Brown M.C., Shrestha U., Huber C., Best L.G., O’Leary M., Howard B., Beresford S., Fretts A.M. (2019). Characterizing the local food environment and grocery-store decision making among a large American Indian community in the north-central USA: Qualitative results from the Healthy Foods Healthy Families Feasibility Study. Public Health Nutr..

[60] Ford J.D., Beaumier M. (2011). Feeding the family during times of stress: Experience and determinants of food insecurity in an Inuit community. Geogr. J..

[61] Jernigan V.B.B., Salvatore A.L., Williams M., Wetherill M., Taniguchi T., Jacob T., Cannady T., Grammar M., Standridge J., Fox J. (2019). A Healthy Retail Intervention in Native American Convenience Stores: The THRIVE Community-Based Participatory Research Study. Am. J. Public Health.

[62] Parker B., Burnett K., Hay T., Skinner K. (2018). The Community Food Environment and Food Insecurity in Sioux Lookout, Ontario: Understanding the Relationships between Food, Health, and Place. J. Hunger. Environ. Nutr..

[63] Jernigan V.B.B., Salvatore A.L., Styne D.M., Winkleby M. (2012). Addressing food insecurity in a Native American reservation using community-based participatory research. Health Educ. Res..

[64] Snowdon W., Raj A., Reeve E., Guerrero R.L., Fesaitu J., Cateine K., Guignet C. (2013). Processed foods available in the Pacific Islands. Glob. Health.

[65] Chodur G.M., Shen Y., Kodish S., Oddo V.M., Antiporta D.A., Jock B., Jones-Smith J.C. (2016). Food Environments around American Indian Reservations: A Mixed Methods Study. PLoS ONE.

[66] Burnett K., Skinner K., Hay T., Leblanc J., Chambers L. (2017). Retail food environments, shopping experiences, First Nations and the provincial Norths. Health Promot. Chronic Dis. Prev. Can..

[67] DyckFehderau D., Holt N., Ball G.D., Willows N.D. (2013). Feasibility study of asset mapping with children: Identifying how the community environment shapes activity and food choices in Alexander First Nation. Rural. Remote. Health.

[68] Fleischhacker S.E., Rodriguez D.A., Evenson K.R., Henley A.C., Gizlice Z., Soto D., Ramachandran G. (2012). Evidence for validity of five secondary data sources for enumerating retail food outlets in seven American Indian Communities in North Carolina. Int. J. Behav. Nutr. Phys. Act..

[69] Smoyer-Tomic K.E., Spence J.C., Raine K.D., Amrhein C., Cameron N., Yasenovskiy V., Cutumisu N., Hemphill E., Healy J. (2008). The association between neighborhood socioeconomic status and exposure to supermarkets and fast food outlets. Health Place.

[70] Sumner J., Tarhan M.D., McMurtry J.J. (2019). Eating in Place: Mapping Alternative Food Procurement in Canadian Indigenous Communities. J. Agric. Food Syst. Community Dev..

[71] Kumar G., Jim-Martin S., Piltch E., Onufrak S.J., McNeil C., Adams L.N., Williams N.I., Blanck H.M., Curley L. (2016). Healthful Nutrition of Foods in Navajo Nation Stores. Am. J. Health Promot..

[72] Matanane L., Fialkowski M.K., Silva J., Li F., Nigg C., Guerrero R.T.L., Novotny R. (2017). Para I Famagu’on-Ta: Fruit and Vegetable Intake, Food Store Environment, and Childhood Overweight/Obesity in the Children’s Healthy Living Program on Guam. Hawai’i J. Med. Public Health J. Asia Pac. Med. Public Health.

[73] O’Connell M., Buchwald D.S., Duncan G.E. (2011). Food Access and Cost in American Indian Communities in Washington State. J. Am. Diet. Assoc..

[74] Galloway T. (2017). Canada’s northern food subsidy Nutrition North Canada: A comprehensive program evaluation. Int. J. Circumpolar Health.

[75] Thompson S., Kamal A.G., Alam M.A., Wiebe J. (2012). Community Development to Feed the Family in Northern Manitoba Communities: Evaluating Food Activities based on Their Food Sovereignty, Food Security, and Sustainable Livelihood Outcomes. Can. J. Nonprofit Soc. Econ. Res..

[76] Brimblecombe J., Bailie R., Boogaard C.V.D., Wood B., Liberato S., Ferguson M., Coveney J., Jaenke R., Ritchie J. (2017). Feasibility of a novel participatory multi-sector continuous improvement approach to enhance food security in remote Indigenous Australian communities. SSM—Popul. Health.

[77] Jackson S.L. (2016). Sodium in Store and Restaurant Food Environments—Guam, 2015. MMWR. Morb. Mortal. Wkly. Rep..

[78] Holden S., Ferguson M., Brimblecombe J., Palermo C. (2015). Can a community of practice equip public health nutritionists to work with remote retail to improve the food supply?. Rural. Remote Health.

[79] Rowley K.G., Su Q., Cincotta M., Skinner M., Skinner K., Pindan B., White G.A., O’Dea K. (2001). Improvements in circulating cholesterol, antioxidants, and homocysteine after dietary intervention in an Australian Aboriginal community. Am. J. Clin. Nutr..

[80] Davis S.M., Sanders S.G., Fitzgerald C.A., Keane P.C., Canaca G.F., Volker-Rector R. (2013). CHILE: An evidence-based preschool intervention for obesity prevention in Head Start. J. Sch. Health.

[81] Brimblecombe J., Ferguson M.M., Chatfield M.M.D., Liberato S.C., Gunther A., Ball K., Moodie D.P.M., Miles E., Magnus P.A., Ni Mhurchu C. (2017). Effect of a price discount and consumer education strategy on food and beverage purchases in remote Indigenous Australia: A stepped-wedge randomised controlled trial. Lancet Public Health.

[82] Lundeen E.A., VanFrank B.K., Jackson S.L., Harmon B., Uncangco A., Luces P., Dooyema C., Park S. (2017). Availability and Promotion of Healthful Foods in Stores and Restaurants—Guam, 2015. Prev. Chronic Dis..

[83] Fretts A.M., Huber C., Best L.G., O’Leary M., Lebeau L., Howard B.V., Siscovick D.S., Beresford S.A. (2018). Availability and Cost of Healthy Foods in a Large American Indian Community in the North-Central United States. Prev. Chronic Dis..

[84] Love C.V., Taniguchi T.E., Williams M.B., Noonan C.J., Wetherill M.S., Salvatore A.L., Jacob T., Cannady T.K., Standridge J., Spiegel J. (2019). Diabetes and Obesity Associated with Poor Food Environments in American Indian communities: The THRIVE study. Curr. Dev. Nutr..

[85] Mullany B., Neault N., Tsingine D., Powers J., Lovato V., Clitso L., Massey S., Talgo A., Speakman K., Barlow A. (2013). Food insecurity and household eating patterns among vulnerable American-Indian families: Associations with caregiver and food consumption characteristics. Public Health Nutr..

[86] Bauer K.W., Widome R., Himes J.H., Smyth M., Rock B.H., Hannan P.J., Story M. (2012). High Food Insecurity and Its Correlates Among Families Living on a Rural American Indian Reservation. Am. J. Public Health.

[87] Jani R., Rush E., Crook N., Simmons D. (2018). Availability and price of healthier food choices and association with obesity prevalence in New Zealand Māori. Asia Pac. J. Clin. Nutr..

[88] Cunningham-Sabo L., Bauer M., Pareo S., Phillips-Benally S., Roanhorse J., Garcia L. (2008). Qualitative Investigation of Factors Contributing to Effective Nutrition Education for Navajo Families. Matern. Child. Health J..

[89] Eyles H., Ni Mhurchu C., Wharemate L., Funaki-Tahifote M., Lanumata T., Rodgers A. (2009). Developing nutrition education resources for a multi-ethnic population in New Zealand. Health Educ. Res..

[90] Verrall T., Napash L., Leclerc L., Mercure S., Gray-Donald K. (2006). Community-based communication strategies to promote infant iron nutrition in northern Canada. Int. J. Circumpolar Health.

[91] Lee A., Darcy A.M., Leonard D., Groos A.D., Stubbs C.O., Lowson S.K., Dunn S.M., Coyne T., Riley M.D. (2002). Food availability, cost disparity and improvement in relation to accessibility and remoteness in Queensland. Aust. N. Zeal. J. Public Health.

[92] Wendimu M.A., Desmarais A.A., Martens T.R. (2018). Access and affordability of “healthy” foods in northern Manitoba? The need for Indigenous food sovereignty. Can. Food Stud./La Revue Can. des Études sur L’alimentation.

[93] Johnson J.S., Nobmann E.D., Asay E. (2012). Factors related to fruit, vegetable and traditional food consumption which may affect health among Alaska Native People in Western Alaska. Int. J. Circumpolar Health.

[94] Wetherill M.S., Williams M.B., Taniguchi T., Salvatore A.L., Jacob T., Cannady T., Grammar M., Standridge J., Fox J., Spiegel J. (2020). A Nutrition Environment Measure to Assess Tribal Convenience Stores: The THRIVE Study. Health Promot. Pr..

[95] Lambden J., Receveur O., Marshall J., Kuhnlein H.V. (2006). Traditional and market food access in Arctic Canada is affected by economic factors. Int. J. Circumpolar Health.

[96] Bhawra J., Cooke M.J., Hanning R.M., Wilk P., Gonneville S.L.H. (2015). Community perspectives on food insecurity and obesity: Focus groups with caregivers of Métis and Off-reserve First Nations children. Int. J. Equity Health.

[97] Jahns L., McDonald L., Wadsworth A., Morin C., Liu Y., Nicklas T. (2015). Barriers and facilitators to following the Dietary Guidelines for Americans reported by rural, Northern Plains American-Indian children. Public Health Nutr..

[98] Joseph P., Davis A.D., Miller R., Hill K., McCarthy H., Banerjee A., Chow C.K., Mente A., Anand S.S. (2012). Contextual determinants of health behaviours in an aboriginal community in Canada: Pilot project. BMC Public Health.

[99] Schiff R., Brunger F. (2013). Northern Food Networks: Building Collaborative Efforts for Food Security in Remote Canadian Aboriginal Communities. J. Agric. Food Syst. Community Dev..

[100] McCarthy L., Chang A.B., Brimblecombe J. (2018). Food Security Experiences of Aboriginal and Torres Strait Islander Families with Young Children in An Urban Setting: Influencing Factors and Coping Strategies. Int. J. Environ. Res. Public Health.

[101] Brimblecombe J.K., O’Dea K. (2009). The role of energy cost in food choices for an Aboriginal population in northern Australia. Med. J. Aust..

[102] Ferguson M., O’Dea K., Chatfield M., Moodie M., Altman J., Brimblecombe J. (2016). The comparative cost of food and beverages at remote Indigenous communities, Northern Territory, Australia. Aust. N. Zeal. J. Public Health.

[103] Campbell M.L., Diamant R.M.F., MacPherson B.D., Halladay J.L. (1997). The Contemporary Food Supply of Three Northern Manitoba Cree Communities. Can. J. Public Health.

[104] Mackay S., Buch T., Vandevijvere S., Goodwin R., Korohina E., Funaki-Tahifote M., Lee A., Swinburn B. (2018). Cost and Affordability of Diets Modelled on Current Eating Patterns and on Dietary Guidelines, for New Zealand Total Population, Māori and Pacific Households. Int. J. Environ. Res. Public Health.

[105] Vandevijvere S., Young N., Mackay S., Swinburn B., Gahegan M. (2018). Modelling the cost differential between healthy and current diets: The New Zealand case study. Int. J. Behav. Nutr. Phys. Act..

[106] Lee A., Lewis M. (2018). Testing the Price of Healthy and Current Diets in Remote Aboriginal Communities to Improve Food Security: Development of the Aboriginal and Torres Strait Islander Healthy Diets ASAP (Australian Standardised Affordability and Pricing) Methods. Int. J. Environ. Res. Public Health.

[107] Johnson-Down L., Willows N., Kenny T.-A., Ing A., Fediuk K., Sadik T., Chan H.M., Batal M. (2019). Optimisation modelling to improve the diets of First Nations individuals. J. Nutr. Sci..

[108] Ni Mhurchu C., Eyles H., Schilling C., Yang Q., Kaye-Blake W., Genç M., Blakely T. (2013). Food Prices and Consumer Demand: Differences across Income Levels and Ethnic Groups. PLoS ONE.

[109] Gawn G., Innes R., Rausser G.C., Zilberman D. (1993). Nutrient demand and the allocation of time: Evidence from Guam. Appl. Econ..

[110] Richards T.J., Patterson P.M. (2006). Native American Obesity: An Economic Model of the “Thrifty Gene” Theory. Am. J. Agric. Econ..

[111] Pollard C.M., Landrigan T.J., Ellies P.L., Kerr D.A., Lester M.L.U., Goodchild S.E. (2014). Geographic factors as determinants of food security: A Western Australian food pricing and quality study. Asia Pac. J. Clin. Nutr..

[112] Eyles H., Rodgers A., Ni Mhurchu C. (2010). Use of electronic sales data to tailor nutrition education resources for an ethnically diverse population. J. Hum. Nutr. Diet..

[113] Simoneau N., Receveur O. (2000). Attributes of Vitamin A- and Calcium-Rich Food Items Consumed in K’asho Got’ine, Northwest Territories, Canada. J. Nutr. Educ..

[114] Fleischhacker S.E., Vu M., Ries A., McPhail A. (2011). Engaging Tribal Leaders in an American Indian Healthy Eating Project Through Modified Talking Circles. Fam. Community Health.

[115] Brimblecombe J., Maypilama E., Colles S., Scarlett M., Dhurrkay J.G., Ritchie J., O’Dea K. (2014). Factors Influencing Food Choice in an Australian Aboriginal Community. Qual. Health Res..

[116] Beaumier M.C., Ford J.D. (2010). Food Insecurity among Inuit Women Exacerbated by Socio-economic Stresses and Climate Change. Can. J. Public Health.

[117] Dammann K.W., Smith C. (2011). Food-Related Environmental, Behavioral, and Personal Factors Associated with Body Mass Index among Urban, Low-Income African-American, American Indian, and Caucasian Women. Am. J. Health Promot..

[118] Beaumier M.C., Ford J.D., Tagalik S. (2015). The food security of Inuit women in Arviat, Nunavut: The role of socio-economic factors and climate change. Polar Rec..

[119] Stroink M.L. (2012). Understanding Local Food Behaviour and Food Security in Rural First Nation Communities: Implications for Food Policy. J. Rural Community Dev..

[120] Gittelsohn J., Toporoff E.G., Story M., Evans M., Anliker J., Davis S., Sharma A., White J. (2000). Food Perceptions and Dietary Behavior of American-Indian Children, Their Caregivers, and Educators: Formative Assessment Findings from Pathways. J. Nutr. Educ..

[121] Sowerwine J., Mucioki M., Sarna-Wojcicki D., Hillman L. (2019). Reframing food security by and for Native American communities: A case study among tribes in the Klamath River basin of Oregon and California. Food Secur..

[122] Vaughan L.A., Benyshek D.C., Martin J.F. (1997). Food Acquisition Habits, Nutrient Intakes, and Anthropometric Data of Havasupai Adults. J. Am. Diet. Assoc..

[123] Genuis S.K., Willows N., Jardine C., Nation A.F. (2015). Through the lens of our cameras: Children’s lived experience with food security in a Canadian Indigenous community. Child. Care Health Dev..

[124] Wycherley T.P., Van Der Pols J.C., Daniel M., Howard N.J., O’Dea K., Brimblecombe J. (2019). Associations between Community Environmental-Level Factors and Diet Quality in Geographically Isolated Australian Communities. Int. J. Environ. Res. Public Health.

[125] MacKenzie O.W., George C.V., Pérez-Escamilla R., Lasky-Fink J., Piltch E.M., Sandman S.M., Clark C., Avalos Q.J., Carroll D.S., Wilmot T.M. (2019). Healthy Stores Initiative Associated with Produce Purchasing on Navajo Nation. Curr. Dev. Nutr..

[126] Rowse T., Scrimgeour D., Knight S., Thomas D. (1994). Food-purchasing behaviour in an Aboriginal community. 1. Results of a survey. Aust. J. Public Health.

[127] Wycherley T.P., Pekarsky B.A., Ferguson M.M., O’Dea K., Brimblecombe J.K. (2017). Fluctuations in money availability within an income cycle impacts diet quality of remote Indigenous Australians. Public Health Nutr..

[128] Scelza B.A., Bird D.W., Bird R.B. (2014). Bush Tucker, Shop Tucker: Production, Consumption, and Diet at an Aboriginal Outstation. Ecol. Food Nutr..

[129] Scelza B.A. (2012). Food scarcity, not economic constraint limits consumption in a rural Aboriginal community. Aust. J. Rural. Health.

[130] Gittelsohn J., Anliker J.A., Sharma S., Vastine A.E., Caballero B., Ethelbah B. (2006). Psychosocial Determinants of Food Purchasing and Preparation in American Indian Households. J. Nutr. Educ. Behav..

[131] Dressler H., Smith C. (2013). Health and Eating Behavior Differs between Lean/Normal and Overweight/Obese Low-Income Women Living in Food-Insecure Environments. Am. J. Health Promot..

[132] Lee A.J., O’Dea K., Mathews J.D. (2010). Apparent dietary intake in remote Aboriginal communities. Aust. J. Public Health.

[133] Brimblecombe J., Liddle R., O’Dea K. (2012). Use of point-of-sale data to assess food and nutrient quality in remote stores. Public Health Nutr..

[134] McMahon E., Wycherley T., O’Dea K., Brimblecombe J. (2017). A comparison of dietary estimates from the National Aboriginal and Torres Strait Islander Health Survey to food and beverage purchase data. Aust. N. Zeal. J. Public Health.

[135] Wycherley T.P., Ferguson M., O’Dea K., McMahon E.J., Liberato S., Brimblecombe J. (2016). Store turnover as a predictor of food and beverage provider turnover and associated dietary intake estimates in very remote Indigenous communities. Aust. N. Zeal. J. Public Health.

[136] Ni Mhurchu C., Blakely T., Wall J., Rodgers A., Jiang Y., Wilton J. (2007). Strategies to promote healthier food purchases: A pilot supermarket intervention study. Public Health Nutr..

[137] Lee A.J., Smith A., Bryce S., O’Dea K., Rutishauser I.H., Mathews J.D. (1995). Measuring dietary intake in remote australian aboriginal communities. Ecol. Food Nutr..

[138] Brimblecombe J., Mackerras D., Clifford P., O’Dea K. (2006). Does the store-turnover method still provide a useful guide to food intakes in Aboriginal communities?. Aust. N. Zeal. J. Public Health.

[139] Wakegijig J., Osborne G., Statham S., Issaluk M.D. (2013). Collaborating toward improving food security in Nunavut. Int. J. Circumpolar Health.

[140] Rogers A., Ferguson M.M., Ritchie J., Boogaard C.V.D., Brimblecombe J.K. (2018). Strengthening food systems with remote Indigenous Australians: Stakeholders’ perspectives. Health Promot. Int..

[141] Brimblecombe J., Boogaard C.H.V.D., Ritchie J., Bailie R., Coveney J., Liberato S.C. (2014). From targets to ripples: Tracing the process of developing a community capacity building appraisal tool with remote Australian indigenous communities to tackle food security. BMC Public Health.

[142] Skinner K., Hanning R.M., Desjardins E., Tsuji L.J.S. (2013). Giving voice to food insecurity in a remote indigenous community in subarctic Ontario, Canada: Traditional ways, ways to cope, ways forward. BMC Public Health.

[143] Calancie L., Stowers K.C., Palmer A., Frost N., Calhoun H., Piner A., Webb K. (2018). Toward a Community Impact Assessment for Food Policy Councils: Identifying Potential Impact Domains. J. Agric. Food Syst. Community Dev..

[144] Signal L., Walton M.D., Ni Mhurchu C., Maddison R., Bowers S.G., Carter K.N., Gorton D., Heta C., Lanumata T.S., McKerchar C.W. (2013). Tackling ’wicked’ health promotion problems: A New Zealand case study. Health Promot. Int..

[145] Chan H.M., Fediuk K., Hamilton S., Rostas L., Caughey A., Kuhnlein H., Egeland G., Loring E. (2006). Food security in Nunavut, Canada: Barriers and recommendations. Int. J. Circumpolar Health.

[146] Skinner K., Hanning R.M., Sutherland C., Edwards-Wheesk R., Tsuji L.J.S. (2012). Using a SWOT Analysis to Inform Healthy Eating and Physical Activity Strategies for a Remote First Nations Community in Canada. Am. J. Health Promot..

[147] Brimblecombe J., Boogaard C.V.D., Wood B., Liberato S.C., Brown J., Barnes A., Rogers A., Coveney J., Ritchie J., Bailie R. (2015). Development of the good food planning tool: A food system approach to food security in indigenous Australian remote communities. Health Place.

[148] Rudolph K.R., McLachlan S.M. (2013). Seeking Indigenous food sovereignty: Origins of and responses to the food crisis in northern Manitoba, Canada. Local Environ..

[149] McDonald E.L., Bailie R.S., Morris P.S. (2017). Participatory systems approach to health improvement in Australian Aboriginal children. Health Promot. Int..

[150] Sowerwine J., Sarna-Wojcicki D., Mucioki M., Hillman L., Lake F.K., Friedman E. (2019). Enhancing Food Sovereignty: A Five-year Collaborative Tribal-University Research and Extension Project in California and Oregon. J. Agric. Food Syst. Community Dev..

[151] Ferguson M., O’Dea K., Altman J., Moodie M., Brimblecombe J. (2018). Health-Promoting Food Pricing Policies and Decision-Making in Very Remote Aboriginal and Torres Strait Islander Community Stores in Australia. Int. J. Environ. Res. Public Health.

[152] Curran S., Gittelsohn J., Anliker J., Ethelbah B., Blake K., Sharma S., Caballero B. (2005). Process evaluation of a store-based environmental obesity intervention on two American Indian Reservations. Health Educ. Res..

[153] Vastine A., Gittelsohn J., Ethelbah B., Anliker J., Caballero B. (2005). Formative Research and Stakeholder Participation in Intervention Development. Am. J. Health Behav..

[154] Sharma S., Gittelsohn J., Rosol R., Beck L. (2010). Addressing the public health burden caused by the nutrition transition through the Healthy Foods North nutrition and lifestyle intervention programme. J. Hum. Nutr. Diet..

[155] Gittelsohn J. (2010). Participatory Research for Chronic Disease Prevention in Inuit Communities. Am. J. Health Behav..

[156] Mead E.L., Gittelsohn J., Roache C., Corriveau A., Sharma S. (2013). A Community-Based, Environmental Chronic Disease Prevention Intervention to Improve Healthy Eating Psychosocial Factors and Behaviors in Indigenous Populations in the Canadian Arctic. Health Educ. Behav..

[157] Gittelsohn J., Kim E.M., He S., Pardilla M. (2013). A Food Store–Based Environmental Intervention Is Associated with Reduced BMI and Improved Psychosocial Factors and Food-Related Behaviors on the Navajo Nation. J. Nutr..

[158] Rosecrans A.M., Gittelsohn J., Ho L.S., Harris S.B., Naqshbandi M., Sharma S. (2008). Process evaluation of a multi-institutional community-based program for diabetes prevention among First Nations. Health Educ. Res..

[159] Ho L.S., Gittelsohn J., Rimal R., Treuth M.S., Sharma S., Rosecrans A., Harris S.B. (2008). An Integrated Multi-Institutional Diabetes Prevention Program Improves Knowledge and Healthy Food Acquisition in Northwestern Ontario First Nations. Health Educ. Behav..

[160] Redmond L.C., Jock B., Gadhoke P., Chiu D.T., Christiansen K., Pardilla M., Swartz J., Platero H., Caulfield L.E., Gittelsohn J. (2019). OPREVENT (Obesity Prevention and Evaluation of InterVention Effectiveness in NaTive North Americans): Design of a Multilevel, Multicomponent Obesity Intervention for Native American Adults and Households. Curr. Dev. Nutr..

[161] Gittelsohn J., Vijayadeva V., Davison N., Ramirez V., Cheung L.W., Murphy S., Novotny R. (2010). A Food Store Intervention Trial Improves Caregiver Psychosocial Factors and Children’s Dietary Intake in Hawaii. Obesity.

[162] Sussman A.L., Davis S.M. (2010). Integrating Formative Assessment and Participatory Research. Am. J. Health Educ..

[163] Cruz T.H., Davis S.M., Fitzgerald C.A., Canaca G.F., Keane P.C. (2014). Engagement, recruitment, and retention in a trans-community, randomized controlled trial for the prevention of obesity in rural American Indian and Hispanic children. J. Prim. Prev..

[164] Blakely T., Ni Mhurchu C., Jiang Y., Matoe L., Funaki-Tahifote M., Eyles H., Foster R.H., McKenzie S., Rodgers A. (2011). Do effects of price discounts and nutrition education on food purchases vary by ethnicity, income and education? Results from a randomised, controlled trial. J. Epidemiol. Community Health.

[165] Scrimgeour D., Rowse T., Knight S. (1994). Food-purchasing behaviour in an Aboriginal community. 2. Evaluation of an intervention aimed at children. Aust. J. Public Health.

[166] Davis S.M., Myers O.B., Cruz T.H., Morshed A.B., Canaca G.F., Keane P.C., O’Donald E.R. (2016). CHILE: Outcomes of a group randomized controlled trial of an intervention to prevent obesity in preschool Hispanic and American Indian children. Prev. Med..

[167] Morrison N., Dooley J. (1998). The Sioux Lookout Diabetes Program: Diabetes prevention and management in northwestern Ontario. Int. J. Circumpolar Health.

[168] Fehring E., Ferguson M., Brown C., Murtha K., Laws C., Cuthbert K., Thompson K., Williams T., Hammond M., Brimblecombe J. (2019). Supporting healthy drink choices in remote Aboriginal and Torres Strait Islander communities: A community-led supportive environment approach. Aust. N. Zeal. J. Public Health.

[169] Brown C., Laws C., Leonard D., Campbell S., Merone L., Hammond M., Thompson K., Canuto K., Brimblecombe J. (2019). Healthy Choice Rewards: A Feasibility Trial of Incentives to Influence Consumer Food Choices in a Remote Australian Aboriginal Community. Int. J. Environ. Res. Public Health.

[170] Ni Mhurchu C., Eyles H., Dixon R., Matoe L., Teevale T., Meagher-Lundberg P. (2012). Economic incentives to promote healthier food purchases: Exploring acceptability and key factors for success. Health Promot. Int..

[171] Snowdon W. (2014). Sugar-sweetened beverages in Pacific Island countries and territories: Problems and solutions?. Pac. Health Dialog.

[172] Ni Mhurchu C., Blakely T., Funaki-Tahifote M., McKerchar C., Wilton J., Chua S., Jiang Y. (2009). Inclusion of indigenous and ethnic minority populations in intervention trials: Challenges and strategies in a New Zealand supermarket study. J. Epidemiol. Community Health.

[173] Black A.P., Vally H., Morris P.S., Daniel M., Esterman A.J., Smith F.E., O’Dea K. (2013). Health outcomes of a subsidised fruit and vegetable program for Aboriginal children in northern New South Wales. Med. J. Aust..

[174] Black A.P., Vally H., Morris P., Daniel M., Esterman A., Karschimkus C.S., O’Dea K. (2013). Nutritional impacts of a fruit and vegetable subsidy programme for disadvantaged Australian Aboriginal children. Br. J. Nutr..

[175] Magnus A., Moodie M.L., Ferguson M., Cobiac L.J., Liberato S.C., Brimblecombe J. (2016). The economic feasibility of price discounts to improve diet in Australian Aboriginal remote communities. Aust. N. Zeal. J. Public Health.

[176] Magnus A., Cobiac L., Brimblecombe J., Chatfield M., Gunther A., Ferguson M., Moodie M. (2018). The cost-effectiveness of a 20% price discount on fruit, vegetables, diet drinks and water, trialled in remote Australia to improve Indigenous health. PLoS ONE.

[177] Ni Mhurchu C., Eyles H., Genç M., Scarborough P., Rayner M., Mizdrak A., Nnoaham K., Blakely T. (2015). Effects of Health-Related Food Taxes and Subsidies on Mortality from Diet-Related Disease in New Zealand: An Econometric-Epidemiologic Modelling Study. PLoS ONE.

[178] Cueva K., Lovato V., Nieto T., Neault N., Barlow A., Speakman K. (2018). Increasing Healthy Food Availability, Purchasing, and Consumption: Lessons Learned from Implementing a Mobile Grocery. Prog. Community Health Partnersh. Res. Educ. Action.

[179] Lotoski L.C., Engler-Stringer R., Muhajarine N. (2015). Cross-sectional analysis of a community-based cooperative grocery store intervention in Saskatoon, Canada. Can. J. Public Health.

[180] McMahon E.J., Webster J., Brimblecombe J. (2017). Effect of 25% Sodium Reduction on Sales of a Top-Selling Bread in Remote Indigenous Australian Community Stores: A Controlled Intervention Trial. Nutrition.

[181] McMahon E.J., Clarke R., Jaenke R., Brimblecombe J. (2016). Detection of 12.5% and 25% Salt Reduction in Bread in a Remote Indigenous Australian Community. Nutrition.

[182] Nhung N.T.H., Blakely T., Cobiac L.J., Pearson A.L., Wilson N. (2015). Health and Economic Impacts of Eight Different Dietary Salt Reduction Interventions. PLoS ONE.

[183] McMahon E.J., Webster J., O’Dea K., Brimblecombe J. (2015). Dietary sodium and iodine in remote Indigenous Australian communities: Will salt-reduction strategies increase risk of iodine deficiency? A cross-sectional analysis and simulation study. BMC Public Health.

[184] Ho L.S., Gittelsohn J., Harris S.B., Ford E. (2006). Development of an integrated diabetes prevention program with First Nations in Canada. Health Promot. Int..

[185] WorldBank How Does the World Bank Classify Countries?. https://datahelpdesk.worldbank.org/knowledgebase/articles/378834-how-does-the-world-bank-classify-countries.

[186] Lytle L.A., Sokol R.L. (2017). Measures of the food environment: A systematic review of the field, 2007–2015. Health Place.

[187] Ploeg M.V., Wilde P.E. (2018). How do food retail choices vary within and between food retail environments?. Food Policy.

[188] Walker R.E., Keane C.R., Burke J.G. (2010). Disparities and access to healthy food in the United States: A review of food deserts literature. Health Place.

[189] Dammann K.W., Smith C. (2009). Factors Affecting Low-income Women’s Food Choices and the Perceived Impact of Dietary Intake and Socioeconomic Status on Their Health and Weight. J. Nutr. Educ. Behav..

[190] Kaufman P., Dicken C., Williams R. (2014). Measuring Access to Healthful, Affordable Food in American Indian and Alaska Native Tribal Areas.

[191] Sacks G., Swinburn B., Kraak V.I., Downs S., Walker C., Barquera S., Friel S., Hawkes C., Kelly B., Kumanyika S. (2013). A proposed approach to monitor private-sector policies and practices related to food environments, obesity and non-communicable disease prevention. Obes. Rev..

[192] Castellari E., Moro D., Platoni S., Sckokai P. (2018). Retailers’ strategies and food price dynamics: Evidence from dairy scanner data. Food Policy.

[193] Lee A.J., Bonson A.P., Powers J.R. (1996). The effect of retail store managers on Aboriginal diet in remote communities. Aust. N. Zeal. J. Public Health.

[194] Ford J.D., Lardeau M.-P., Vanderbilt W. (2012). The characteristics and experience of community food program users in arctic Canada: A case study from Iqaluit, Nunavut. BMC Public Health.

[195] Cooksey-Stowers K., Schwartz M.B., Brownell K.D. (2017). Food Swamps Predict Obesity Rates Better Than Food Deserts in the United States. Int. J. Environ. Res. Public Health.

[196] Hager E.R., Cockerham A., O’Reilly N., Harrington D., Harding J., Hurley K.M., Black M.M. (2016). Food swamps and food deserts in Baltimore City, MD, USA: Associations with dietary behaviours among urban adolescent girls. Public Health Nutr..

[197] Budzynska K., West P., Savoy-Moore R.T., Lindsey D., Winter M., Newby P.K. (2013). A food desert in Detroit: Associations with food shopping and eating behaviours, dietary intakes and obesity. Public Health Nutr..

[198] Trewin D. (2006). National Aboriginal and Torres Strait Islander Health Survey, 2004–2005.

[199] Zienczuk N., Young T.K., Cao Z.R., Egeland G.M. (2012). Dietary correlates of an at-risk BMI among Inuit adults in the Canadian high arctic: Cross-sectional international polar year Inuit health survey, 2007–2008. Nutr. J..

[200] Maru Y.T., Smith M.S., Sparrow A., Pinho P.F., Dube O.P. (2014). A linked vulnerability and resilience framework for adaptation pathways in remote disadvantaged communities. Glob. Environ. Chang..

[201] Singh-Peterson L., Lieske S., Underhill S.J.R., Keys N. (2015). Food security, remoteness and consolidation of supermarket distribution centres: Factors contributing to food pricing inequalities across Queensland, Australia. Aust Geogr..

[202] Novotny R., Vijayadeva V., Ramirez V., Lee S.K., Davison N., Gittelsohn J. (2011). Development and Implementation of a Food System Intervention to Prevent Childhood Obesity in Rural Hawai‘i. Hawaii Med. J..

[203] Whyte K., Barnhill A., Doggett T., Budolfson M. (2017). Food Sovereignty, Justice and Indigenous Peoples: An Essay on Settler Colonialism and Collective Continuance. Oxford Handbook on Food Ethics.

[204] Daher O., Hannikainen L., Heikinheimo-Pérez K. (2016). National Minorities in Finland–Richness of Cultures and Languages.

[205] Daigle M. (2017). Tracing the terrain of Indigenous food sovereignties. J. Peasant. Stud..

[206] Coté C. (2016). “Indigenizing” Food Sovereignty. Revitalizing Indigenous Food Practices and Ecological Knowledges in Canada and the United States. Humanities.

[207] Harder M.T., Wenzel G.W. (2012). Inuit Subsistence, Social Economy and Food Security in Clyde River, Nunavut. Arctic.

[208] Wenzel G.W. (2013). Inuit and modern hunter-gatherer subsistence. Études/Inuit/Studies.

[209] Jonasson M.E., Spiegel S.J., Thomas S., Yassi A., Wittman H., Takaro T., Afshari R., Markwick M., Spiegel J.M. (2019). Oil pipelines and food sovereignty: Threat to health equity for Indigenous communities. J. Public Health Policy.

[210] Simpson L. (2003). Toxic contamination undermining Indigenous food systems and Indigenous sovereignty. Pimatiziwin J. Aborig. Indig. Community Health.

[211] Parlee B., Sandlos J., Natcher D. (2018). Undermining subsistence: Barren-ground caribou in a “tragedy of open access”. Sci. Adv..

[212] Whyte K. (2016). Indigenous Food Sovereignty, Renewal and U.S. Settler Colonialism. The Routledge Handbook of Food Ethics.

[213] Whyte K., Rawlinson M., Ward C. (2015). Indigenous Food Systems, Environmental Justice, and Settler-Industrial States. Global Food, Global Justice: Essays on Eating under Globalization.

[214] Seymour J.D. (2004). Impact of nutrition environmental interventions on point-of-purchase behavior in adults: A review. Prev. Med..

[215] Ni Mhurchu C., Blakely T., Jiang Y., Eyles H.C., Rodgers A. (2010). Effects of price discounts and tailored nutrition education on supermarket purchases: A randomized controlled trial. Am. J. Clin. Nutr..

[216] St-Germain A.-A.F., Galloway T., Tarasuk V. (2019). Food insecurity in Nunavut following the introduction of Nutrition North Canada. Can. Med. Assoc. J..

[217] Socha T., Chambers L., Zahaf M., Abraham R., Fiddler T. (2011). Food availability, food store management, and food pricing in a northern community First Nation community. Int. J. Humanit. Soc. Sci..

[218] Socha T., Zahaf M., Chambers L., Abraham R., Fiddler T. (2012). Food Security in a Northern First Nations Community: An Exploratory Study on Food Availability and Accessibility. J. Aborig. Health.

[219] Pollard C.M. (2012). Selecting Interventions for Food Security in Remote Indigenous Communities. Food Security in Australia.

[220] House of Representatives Standing Committee on Aboriginal and Torres Strait Islander Affairs (2009). Everybody’s Business: Remote Aboriginal and Torres Strait Community Stores.

[221] Niclasen B., Molcho M., Arnfjord S., Schnohr C. (2013). Conceptualizing and contextualizing food insecurity among Greenlandic children. Int. J. Circumpolar Health.

[222] Wetherill M.S., Williams M.B., Hartwell M.L., Salvatore A.L., Jacob T., Cannady T.K., Standridge J., Fox J., Spiegel J., Anderson N. (2018). Food choice considerations among American Indians living in rural Oklahoma: The THRIVE study. Appetite.

[223] Barr T.L., Reid J., Catska P., Varona G., Rout M. (2018). Development of indigenous enterprise in a contemporary business environment—The Ngāi Tahu Ahikā approach. J. Enterp. Communities People Places Glob. Econ..

[224] Reid J., Rout M. (2016). Getting to know your food: The insights of indigenous thinking in food provenance. Agric. Hum. Values.

[225] Bodirsky M., Johnson J. (2008). Decolonizing Diet: Healing by Reclaiming Traditional Indigenous Foodways. Cuizine J. Can. Food Cultures/Cuizine.

[226] Figueroa-Helland L., Thomas C., Aguilera A.P. (2018). Decolonizing Food Systems: Food Sovereignty, Indigenous Revitalization, and Agroecology as Counter-Hegemonic Movements. Perspect. Glob. Dev. Technol..

[227] QIA (2019). Food Sovereignty and Harvesting.

[228] Grey S., Newman L. (2018). Beyond culinary colonialism: Indigenous food sovereignty, liberal multiculturalism, and the control of gastronomic capital. Agric. Hum. Values.

[229] Kuhnlein H., Erasmus B., Creed-Kanashiro H.M., Englberger L., Okeke C., Turner N., Allen L., Bhattacharjee L. (2007). Indigenous peoples’ food systems for health: Finding interventions that work. Public Health Nutr..

[230] Egeland G.M. (2010). Inuit Health Survey 2007–2008: Inuvialuit Settlement Region.

[231] Egeland G.M. (2010). Inuit Health Survey 2007–2008: Nunatsiavut.

[232] Egeland G.M. (2010). Inuit Health Survey 2007–2008: Nunavut.

[233] Reyes-García V., Powell B., Díaz-Reviriego I., Fernández-Llamazares Á., Gallois S., Gueze M. (2019). Dietary transitions among three contemporary hunter-gatherers across the tropics. Food Secur..

[234] Batal M., Johnson-Down L., Moubarac J.-C., Ing A., Fediuk K., Sadik T., Tikhonov C., Chan L., Willows N. (2017). Quantifying associations of the dietary share of ultra-processed foods with overall diet quality in First Nations peoples in the Canadian provinces of British Columbia, Alberta, Manitoba and Ontario. Public Health Nutr..

[235] First Nations Development Institute (2016). Indian Country Food Price Index: Exploring Variation in Food Pricing Across Native Communities—A Working Paper.

[236] First Nations Development Institute (2018). Indian Country Food Price Index: Exploring Variation in Food Pricing Across Native Communities—A Working Paper II.

[237] Grégoire G., Derderian F., Le Lorier J. (1995). Selecting the language of the publications included in a meta-analysis: Is there a tower of babel bias?. J. Clin. Epidemiol..

[238] Kumanyika S.K. (2019). Unraveling common threads in obesity risk among racial/ethnic minority and migrant populations. Public Health.

[239] Searles N. (2016). To sell or not to sell: Country food markets and Inuit identity in Nunavut. Food Foodways.

[240] Ford J.D., Macdonald J.P., Huet C., Statham S., MacRury A. (2016). Food policy in the Canadian North: Is there a role for country food markets?. Soc. Sci. Med..

[241] David-Chavez D.M., Gavin M.C. (2018). A global assessment of Indigenous community engagement in climate research. Environ. Res. Lett..

[242] ITK (2017). An Inuit-Specific Approach for the Canadian Food Policy.

[243] ICC-Alaska (2015). Alaskan Inuit food SECURITY Conceptual Framework: How to assess the Arctic from an Inuit Perspective.

[244] Ferguson M., King A., Brimblecombe J. (2016). Time for a shift in focus to improve food affordability for remote customers. Med. J. Aust..

