# Supplementary material for: The Retail Food Sector and Indigenous Peoples in High-Income Countries: A Systematic Scoping Review
Source: Int J Environ Res Public Health. 2020 Nov 27;17(23):8818. doi: 10.3390/ijerph17238818 (PMC7730644; doi:10.3390/ijerph17238818)
Supplement: Supplementary file 1 [file ijerph-17-08818-s001.pdf]

International Journal of
Environmental Research and Public Health

MDPI

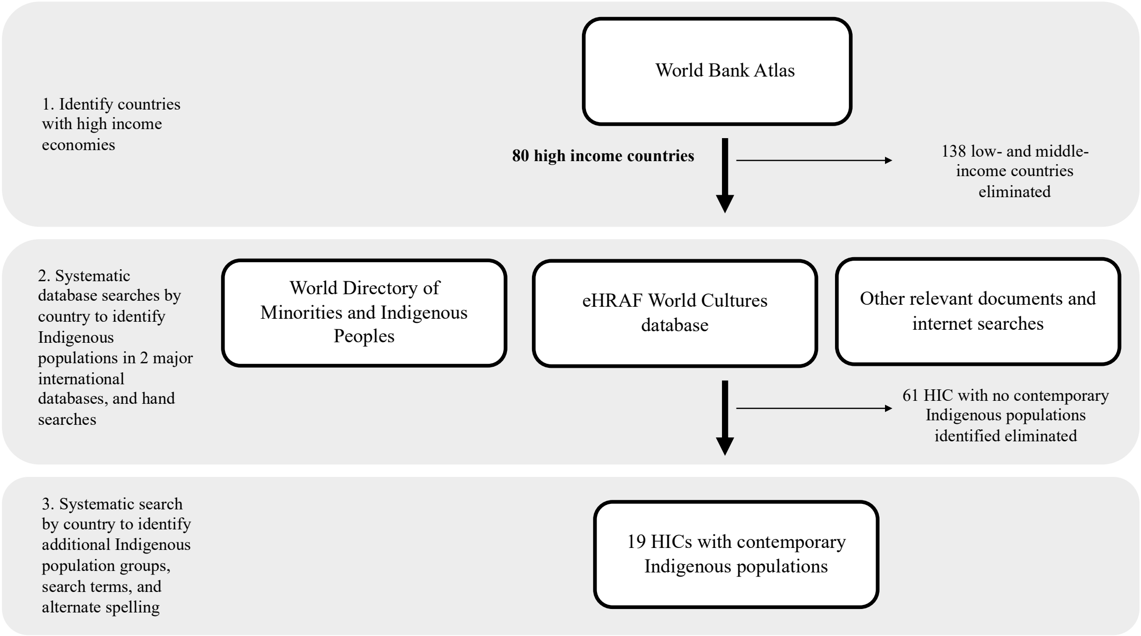

Figure S1. Process of identifying Indigenous Peoples in high income countries.

Following the the approach described by Cisneros Montemayor et al. [1]

Table S1. Identification of Indigenous Peoples in high income countries.

High Income Countries[1]	Included in review [2]	Indigenous Peoples[3]			
		World Directory of Minorities and Indigenous Peoples[4]	eHRAF World Cultures database[5]	The Lancet–Lowitja Institute Global Collaboration[6]	Other searches[7]
Andorra		-	-	-	
Antigua and Barbuda		-	-	-	
Aruba		-	-	-	Arawak
Australia	✓	Aboriginal peoples, South Sea Islanders, Torres Strait Islanders	Yes	Aboriginal and Torres Strait Islanders	
Austria		-	-	-	
Bahamas, The		-	-	-	
Bahrain		-	-	-	
Barbados		-	-	-	
Belgium		-	-	-	
Bermuda		-	-	-	
Brunei Darussalam	✓	Belait, Bisaya, Brunei, Dusun, Kedayan, Murut, Tutong, Iban, Penan	-	-	
Canada	✓	First Nations, Inuit, Métis	Yes	First Nations, Inuit, Métis	
Cayman Islands		-	-	-	
Channel Islands		-	-	-	
Chile	✓	Aymara, Mapuche, Rapanui, others (Yamana, Qawasqar)	Mapuche, Rapa Nui, Tehuelche, Ona, Yahgan; Inka	Mapuche, Alacalufe (Kawaskar), Atacameño, Aymara, Colla, Quechua, Rapanui, Yámana/Yagán, Coya, Kawesqár Yagan, Diaguita	
Croatia		-	-	-	
Curacao		-	-	-	
Cyprus		-	-	-	
Czech Republic		-	-	-	
Denmark		-	-	-	
Estonia		-	-	-	
Faroe Islands		-	-	-	
Finland	✓	Sámi	Saami	-	
France		-	-	-	
French Polynesia	✓	Polynesians	Marquesas	-	
Germany		-	-	-	
Gibraltar		-	-	-	
Greece		-	-	-	
Greenland	✓	Inuit	-	Inuit	
Guam	✓	Chamorros	-	-	
Hong Kong SAR, China		No results	-	-	
Hungary		-	-	-	
Iceland		-	-	-	
Ireland		-	-	-	
Isle of Man		-	-	-	
Israel	✓	Bedouin	-	-	
Italy		-	-	-	

High Income Countries[1]	Included in review [2]	Indigenous Peoples[3] World Directory of Minorities and Indigenous Peoples[4]	eHRAF World Cultures database[5]	The Lancet–Lowitja Institute Global Collaboration[6]	Other searches[7]
Japan	✓ Ainu	Ryūkyūans	Okinawans, Okayama, Ainu	-	
Korea, Rep.		-	-	-	
Kuwait		Bidoon	Bedouin	-	
Latvia		-	-	-	
Liechtenstein		-	-	-	
Lithuania		-	-	-	
Luxembourg		-	-	-	
Macao SAR, China		-	-	-	
Malta		-	-	-	
Monaco		-	-	-	
Netherlands		Moluccans	-	-	
New Caledonia	✓	Melanesians, Polynesians	Kanaks	-	
New Zealand	✓	Māori, Pacific Islanders	Maori	Māori	
Northern Mariana Islands	✓	No results	-	-	Chamorros
Norway	✓	Sámi	Saami	Sámi	
Oman		-	-	-	
Palau	✓	Palauan	Belauans	-	
Panama	✓	Ngäbe (Guaymi), Buglé, Kuna, Emberá (Chocó), Wounaan, Naso/Teribe, Bokota, Bri Bri	Kuna, Talamancans	Kuna Yala, Emberá-Wounaan; Ngäbe Buglé, Kuna, Ngäbe Buglé, Teribe/Naso, Bokota, Emberá, Wounaan, Bri Bri	
Poland		-	-	-	
Portugal		-	-	-	
Puerto Rico		(Afro Puerto-ricans) Taíno	-	-	
Qatar		-	-	-	
San Marino		-	-	-	
Saudi Arabia		-	Bedouin		
Seychelles		-	-	-	
Singapore		Malay	-	-	
Sint Maarten (Dutch part)		-	-	-	
Slovak Republic		-	-	-	
Slovenia		-	-	-	
Spain		-	-	-	
St. Kitts and Nevis		-	-	-	
St. Martin (French part)		-	-	-	
Sweden	✓	Sámi	Saami	-	
Switzerland		-	-	-	
Taiwan, China	✓	Atayal, Bunun, Kavalan, Tsou, Paiwan, Rukai, Puyuma, Amis, Yami (or Tao), Saisiyat,	Atayal, Taiwan Hokkien	-	

High Income Countries[1]	Included in review [2]	Indigenous Peoples[3]			
		World Directory of Minorities and Indigenous Peoples[4]	eHRAF World Cultures database[5]	The Lancet–Lowitja Institute Global Collaboration[6]	Other searches[7]
		Thao, Truku (Taroko), Sakizaya			
Trinidad and Tobago		Taino	Garifuna	-	
Turks and Caicos Islands		Taino	-	-	
United Arab Emirates		-	-	-	
United Kingdom		-	-	-	
United States	✓	Inuit and Alaska Natives, Native Americans, Native Hawai'ians (Kanaka Maoli)	Several	American Indians and Alaska Natives, Native Hawaiians, other Pacific Islanders	
Uruguay		Guaraní Mbyá, Charrúa	No results	-	
Virgin Islands (British)		-	No results	-	
Virgin Islands (U.S.)		-	No results	-	

[1] A country with a high-income economy is defined by the World Bank as a country with a gross national income per capita of US$12,535 or more in 2019. Income is measured using gross national income (GNI) per capita, in U.S. dollars, converted from local currency using the World Bank Atlas method [2].

[2] In the context of the present study, populations of interest included ethnic/cultural groups who are the original or earliest known inhabitants of an area, and/or populations who maintain historical continuity with pre-colonial and/or pre-settler societies. These characteristics were used to distinguish Indigenous Peoples from other minority groups.

[3] A universal definition of “Indigenous” people has not been adopted by any UN-system body. Instead, the system has developed a contemporary understanding of this term based on several factors such as self-identification, historical continuity with pre-colonial and/or pre-settler societies, distinct social, economic or political systems, languages, cultures and beliefs [3]. In some countries, there may be preference for other terms including tribes, first peoples/nations, etc.

[4] World Directory of Minorities and Indigenous Peoples [4]

[5] The eHRAF World Cultures database contains ethnographic collections organized by regions, subregions, and cultures [5]

[6] The Lancet–Lowitja Institute Global Collaboration on Indigenous and tribal peoples' health [6]

[7] Other internet searches to identify national census and demographic information by country.

Table S2. PRISMA-ScR Checklist.

SECTION	ITEM	PRISMA-ScR CHECKLIST ITEM	REPORTED ON PAGE #
**TITLE**			
Title	1	Identify the report as a scoping review.	1
**ABSTRACT**			
Structured summary	2	Provide a structured summary that includes (as applicable): background, objectives, eligibility criteria, sources of evidence, charting methods, results, and conclusions that relate to the review questions and objectives.	1
**INTRODUCTION**			
Rationale	3	Describe the rationale for the review in the context of what is already known. Explain why the review questions/objectives lend themselves to a scoping review approach.	3-4
Objectives	4	Provide an explicit statement of the questions and objectives being addressed with reference to their key elements (e.g., population or participants, concepts, and context) or other relevant key elements used to conceptualize the review questions and/or objectives.	3-4
**METHODS**			
Protocol and registration	5	Indicate whether a review protocol exists; state if and where it can be accessed (e.g., a Web address); and if available, provide registration information, including the registration number.	N/A
Eligibility criteria	6	Specify characteristics of the sources of evidence used as eligibility criteria (e.g., years considered, language, and publication status), and provide a rationale.	5; Table S5
Information sources	7	Describe all information sources in the search (e.g., databases with dates of coverage and contact with authors to identify additional sources), as well as the date the most recent search was executed.	5
Search	8	Present the full electronic search strategy for at least 1 database, including any limits used, such that it could be repeated.	Table S4

SECTION	ITEM	PRISMA-ScR CHECKLIST ITEM	REPORTED ON PAGE #
Selection of sources of evidence	9	State the process for selecting sources of evidence (i.e., screening and eligibility) included in the scoping review.	5
Data charting process	10	Describe the methods of charting data from the included sources of evidence (e.g., calibrated forms or forms that have been tested by the team before their use, and whether data charting was done independently or in duplicate) and any processes for obtaining and confirming data from investigators.	Table S6
Data items	11	List and define all variables for which data were sought and any assumptions and simplifications made.	Table S6
Critical appraisal of individual sources of evidence	12	If done, provide a rationale for conducting a critical appraisal of included sources of evidence; describe the methods used and how this information was used in any data synthesis (if appropriate).	N/A
Synthesis of results	13	Describe the methods of handling and summarizing the data that were charted.	5
**RESULTS**			
Selection of sources of evidence	14	Give numbers of sources of evidence screened, assessed for eligibility, and included in the review, with reasons for exclusions at each stage, ideally using a flow diagram.	5-6
Characteristics of sources of evidence	15	For each source of evidence, present characteristics for which data were charted and provide the citations.	Table S7
Critical appraisal within sources of evidence	16	If done, present data on critical appraisal of included sources of evidence (see item 12).	N/A
Results of individual sources of evidence	17	For each included source of evidence, present the relevant data that were charted that relate to the review questions and objectives.	6-26
Synthesis of results	18	Summarize and/or present the charting results as they relate to the review questions and objectives.	6-26
**DISCUSSION**			

SECTION	ITEM	PRISMA-ScR CHECKLIST ITEM	REPORTED ON PAGE #
Summary of evidence	19	Summarize the main results (including an overview of concepts, themes, and types of evidence available), link to the review questions and objectives, and consider the relevance to key groups.	26
Limitations	20	Discuss the limitations of the scoping review process.	29
Conclusions	21	Provide a general interpretation of the results with respect to the review questions and objectives, as well as potential implications and/or next steps.	30
**FUNDING**			
Funding	22	Describe sources of funding for the included sources of evidence, as well as sources of funding for the scoping review. Describe the role of the funders of the scoping review.	31

JBI = Joanna Briggs Institute; PRISMA-ScR = Preferred Reporting Items for Systematic reviews and Meta-Analyses extension for Scoping Reviews.

Table S3. Literature search themes and combinations of search terms.

Theme	Search terms [1]	Combinations
Country	• Australia<br>• Brunei Darussalam<br>• Canada<br>• Chile<br>• Finland /Finnish<br>• French Polynesia<br>• Greenland<br>• Guam<br>• Israel<br>• Japan<br>• New Caledonia<br>• New Zealand<br>• Northern Mariana Island<br>• Norway<br>• Palaua<br>• Panama<br>• Swed<br>• Taiwan<br>• United States	Australi* OR "Brunei Darussalam" OR Canad* OR Chile* OR Finland OR Finnish OR "French Polynesia*" OR Greenland* OR Guam* OR Israel OR Japan* OR "New Caledonia*" OR "New Zealand*" OR "Northern Mariana Island*" OR Norw* OR Palau* OR Panam* OR Swed* OR Taiwan* OR "United States" OR "American"
Indigenous Peoples[2]	• Aboriginal<br>• Autochthonous<br>• First People<br>• Indigenous<br>• Native<br>• Melanesian<br>• Pacific Islanders (Pasifika, Pacific people)<br>• Polynesian<br>• Tribal	Aboriginal* OR Autocht* OR "First people" OR Indigenous OR Native*OR Melanesian OR "Pacific Islander*" OR Pasifika OR "Pacific people" OR Melanesian* OR Polynesian* OR Trib*
Australia	• Australian Aboriginal<br>• Torres Strait Islander	"Australian Aboriginal*" OR "Aboriginal Australian*" OR "Torres Strait Islander*"
Brunei Darussalam	• Belait<br>• Bisaya<br>• Brunei<br>• Dusun<br>• Kedayan<br>• Murut<br>• Tutong<br>• Iban<br>• Penan	Belait OR Bisaya OR Brunei OR Dusun OR Kedayan OR Murut OR Tutong OR Iban OR Penan
Canada	• First Nation<br>• Inuit<br>• Métis	"First Nation*" OR Inuit OR Métis
Chile	• Atacameño<br>• Aymara<br>• Colla (Kolla, Qulla)<br>• Diaguita<br>• Mapuche<br>• Quechua<br>• Qawasqar (Kawesqar, Kawaskar, Kawesqár, Alacalufe)<br>• Rapanui (Rapa Nui)	Atacameño OR Aymara OR Colla OR Kolla OR Qulla OR Diaguita OR Mapuche OR Quechua OR Qawasqar OR Kawesqar OR Kawaskar OR Kawesqár OR Alacalufe OR Rapanui OR "Rapa Nui" OR Yámana OR Yagán OR Yagan OR Yahgan

Theme	Search terms [1]	Combinations
	• Yámana (Yagán, Yagan, Yahgan, Tequenica)	
Finland, Norway and Sweden	• Sami (Saami)	Sami OR Saami OR Sapmi
French Polynesia	• French Polynesian<br>• Polynesian*<br>• Marquesan*	"French Polynesia*" OR Marquesan*
Greeland	• Inuit<br>• Greenlanders	Inuit OR Greenland*
Guam	• Chamorros	Chamarro* OR Guam
Israel	• Bedouin (Bedu)	Bedouin OR Bedu
Japan	• Ainu (Aynu)	Ainu OR Aynu
New Calodoneia	• Kanak	Kanak* OR "New Calodonei*"
New Zealand	• Māori	Māori
Northern Mariana Islands	• Chamorros	Chamarro* OR "Northern Mariana Islands"
Palau	• Palauan	Palau*
Panama	• Bribri<br>• Bokota<br>• Emberá<br>• Guna (Kuna)<br>• Naso (Terribe)<br>• Ngäbe-Buglé (Guaymi)<br>• Wounaan	Bribri OR Bokota OR Emberá OR Guna OR Kuna OR Naso (NOT Naso-) OR Terribe OR "Ngäbe-Buglé" OR Guaymi OR Wounaan OR<br><br>"Comarca indígenas" OR comarca*<br><br>NOT TS=("NASO-*")
Taiwan	• Amis<br>• Atayal<br>• Bunun<br>• Hla'alua<br>• Kanakaravu<br>• Kavalan<br>• Paiwan<br>• Puyuma<br>• Rukai<br>• Saisiyat<br>• Sakizaya<br>• Seediq<br>• Thao<br>• Truku (Taroko)<br>• Tsou<br>• Yami (Tao)	Amis OR Atayal OR Bunun OR "Hla'alua" OR Kanakaravu OR Kavalan OR Paiwan OR Puyuma OR Rukai OR Saisiyat OR Sakizaya OR Seediq OR Thao OR Truku OR Taroko OR Tsou OR Yami OR Tao

Theme	Search terms [1]	Combinations
United States	• Alaska Natives<br>• Native Americans (American Indians)<br>• Native Hawai'ians (Kanaka Maoli)	"Alaska* Native*" OR "Native American*" OR "American Indian*" OR "Native Hawai'ian*" OR "Kanaka Maoli"
Food environment and supply	• Food system<br>• Food environment<br>• Food supply<br>• Food swamp<br>• Food desert<br>• Food basket<br>• Food availability<br>• Food affordability<br>• Food cost<br>• Food price<br>• Food subsidy<br>• Food quality<br>• Consumer food environment<br>• Diet cost<br>• Diet affordability<br>• Obesogenic	"Food system" OR "Food environment" OR "Food supply" OR "Food swamp" OR "Food desert" OR "Obesogenic" "Food availability" OR "Food affordability" OR "Food cost" OR "Food price*" OR "Food subsid*" OR "Food quality" OR "Consumer food environment*" OR (TS=(Food*) AND TS=(Market* OR Retail OR Store* OR Vendor* OR Demand OR Expenditure OR Sale OR Purchase OR Owner OR Manager)))
Grocery store	• Grocer<br>• Supermarket	Grocer* OR Supermarket
Food	• Food	Food*
Market food system	• Market<br>• Retail<br>• Store<br>• Vendor<br>• Demand<br>• Expenditure<br>• Sale<br>• Purchase<br>• Owner<br>• Manager	(Market OR Retail OR Store* OR Vendor* OR Demand OR Expenditure* OR Sale* OR Purchase* OR Owner OR Manager*)

[1] Search terms are listed alphabetically; for terms that are repeated for different countries or population groups, only the first instance is listed in the table. Search terms were used to identify published articles from the last three decades. Accordingly, these terms may not reflect the contemporary/accepted/preferred designation by the Indigenous population.

Table S4. Literature search strategy and results.

Search no.	Database	Search description	Type of search	Search retrictions	Number of results	Post search filters applied
1	Web of Science (Core Collection)	((Indigenous Peoples – general AND Country) OR (Indigenous Peoples – specific)) AND<br><br>Food environment and supply OR Grocery store OR (Food + Market system)	Topic Search (TS) = Title<br>Abstract<br>Author Keywords<br>Keywords Plus	Year = 1990 – 2020<br>Language = English<br>Document type = article	**1059** → 820 with post search filters applied	Subject exclusions<br>Web of Science categories (no. of articles)<br>• Marine freshwater biology (102)<br>• Oceanography (63)<br>• Geography physical (27)<br>• Entomology (23)<br>• Geosciences multidisciplinary (22)<br>• Zoology (22)<br>• Toxicology (19)<br>• Plant Sciences (17)<br>• Chemistry applied (14)
2	Web of Science (Medline)	((Indigenous Peoples – general AND Country) OR (Indigenous Peoples – specific)) AND<br><br>Food environment and supply OR Grocery store OR (Food + Market system)	Topic Search (TS) = Title<br>Abstract<br>Author Keywords<br>Keywords Plus	Year = 1990 – 2020<br>Language = English<br>Species = human	**701** → 541 with post search filters applied	Research areas excluded (no. of articles)<br>• Plant Sciences (61)<br>• Toxicology (59)<br>• Zoology (36)

Sample search strings used in database searches

INDIGENOUS PEOPLES IN HIC

((TS= (Australi* OR “Brunei Darussalam” OR Canad* OR Chile* OR Finland OR Finnish OR “French Polynesia*” OR Greenland* OR Guam* OR Israel OR Japan* OR “New Caledonia*” OR “New Zealand*” OR “Northern Mariana Island*” OR Norw* OR Palau* OR Panam* OR Swed* OR Taiwan* OR “United States” OR “American”) **AND**

TS=(Aboriginal* OR Autocht* OR “First people” OR Indigenous OR Native*OR Melanesian OR “Pacific Islander*” OR Pasifika OR “Pacific people” OR Melanesian* OR Polynesian* OR Trib*)**) OR**

(TS=("Australian Aboriginal*" OR "Aboriginal Australian*" OR "Torres Strait Islander*" OR Belait OR Bisaya OR Brunei OR Dusun OR Kedayan OR Murut OR Tutong OR Iban OR Penan OR "First Nation*" OR Inuit OR Métis OR Atacameño OR Aymara OR Colla OR Kolla OR Qulla OR Diaguita OR Mapuche OR Quechua OR Qawasqar OR Kawesqar OR Kawaskar OR Kawesqár OR Alacalufe OR Rapanui OR "Rapa Nui" OR Yámana OR Yagán OR Yagan OR Yahgan OR Sami OR Saami OR Sapmi OR "French Polynesia*" OR Marquesan* OR Inuit OR Greenland* OR Chamarro* OR Guam OR Bedouin OR Bedu Ainu OR Aynu OR Kanak* OR "New Calodonei*"OR Māori OR Chamarro* OR "Northern Mariana Islands" OR Bribri OR Bokota OR Emberá OR Guna OR Kuna OR Naso OR Terribe OR "Ngäbe-Buglé" OR Guaymi OR Wounaan OR "Comarca indígenas" OR comarca* OR Amis OR Atayal OR Bunun OR "Hla'alua" OR Kanakaravu OR Kavalan OR Paiwan OR Puyuma OR Rukai OR Saisiyat OR Sakizaya OR Seediq OR Thao OR Truku OR Taroko OR Tsou OR Yami OR Tao OR "Alaska* Native*" OR "Native American*" OR "American Indian*" OR "Native Hawai'ian*" OR "Kanaka Maoli") **NOT TS=(** "NASO-*"**)))**

ALL FOOD RELATED TERMS

(TS=(“Food system” OR “Food environment” OR “Food supply” OR “Food swamp” OR “Food desert” OR “Food basket” OR “Food availability” OR “Food affordability” OR “Food cost” OR “Food price*” OR “Food subsid*” OR “Food quality” OR “Consumer food environment*” OR “Diet cost” OR “Diet affordability” OR “Obesogenic”**) OR**

TS=(Grocer* OR Supermarket) **OR (TS=**(Food*) **AND**

TS=(Market* OR Retail OR Store* OR Vendor* OR Demand OR Expenditure* OR Sale* OR Purchase* OR Owner* OR Manager***)))**

Table S5. Eligibility criteria for screening.

Criteria	Include	Exclude
**General document information**		
Year of publication	- 1990 – 2020 (inclusive)	- Before 1990
Language of publication	- English language only	- All other languages
Document type	- Peer-reviewed journal publications	- Grey literature
**Study type**		
Article type	- Original research<br>- Case studies (descriptive articles of store-based interventions)	- Abstracts<br>- Book chapters<br>- Commentaries and editorials<br>- Reviews<br>- Study protocol
Design	- Qualitative<br>- Quantitative<br>- Multiple and mixed methods	- Descriptive (unless describing store-based intervention study), discursive/argumentative, articles
**Setting**		
Countries	- High income countries[1]	- Middle-income countries<br>- Low-income countries
Geography	- All	
Population	- Indigenous communities or populations<br>- Multi-ethnic study populations including Indigenous populations<br>- General populations/settings where Indigenous Peoples are a majority of the population<br>- Local workforce in Indigenous communities (e.g. store managers, dietitians/nutritionists)	- General populations (no specific mention or inclusion of Indigenous Peoples or communities) or regions/settings
**Focus**		
Food and beverage type	- Market food<br>- General / all foods<br>- Specific foods (e.g. fruit and vegetables, sugar sweetened beverages)	- Traditional/subsistence foods that are not sold in stores and do not involve monetary exchange<br>- Locally-produced/harvested (agriculture, fisheries) and other traditional foods sold in local markets/stores<br>- Food from community gardens and other local initiatives that are not sold in stores and do not involve monetary exchange<br>- Non-food items (e.g. cigarettes)<br>- Alcohol
Store type / food purchase setting	- Local stores, including:<br>o Grocery stores<br>o Supermarkets<br>o Non-traditional food stores (e.g. convenience stores, gas stations)<br>o Farmer's markets<br>- Out-shopping (i.e. orders placed outside of the community or travel to other locations to shop)	- Other community food environment settings (e.g. schools, daycares, restaurants)<br>-
Study focus	- Retail food sector<br>- Retail food supply chain<br>- Food stores<br>- Food supply<br>- Strategies (interventions, policies, initiatives, strategic planning processes) to improve elements of the retail food sector (e.g. obesity and diabetes prevention, food security/system improvement)	- Assessments of diet, food security, health that do not feature an explicit link to the market food sector – even when conducted in the supermarket, or as formative research in an environmental food store intervention<br>- Public health initiatives that do not feature explicit consideration of retail/store food interventions<br>- Educational components that are not necessarily in the store<br>- Smart-phone apps

[1] High Income Countries (HICs) as defined by the World Bank Atlas method (GNI per capita of $12,376) for 2020.

Table S6. Data charting scheme.

Category	Criteria	Information abstracted and categories	Simplification and assumptions
**Reference**	Reference information	- First author<br>- Year of publication	
**Place**	Country	- Country or territory	
	Geography	- Arctic and northern<br>- Island<br>- Remote<br>- Rural<br>- Urban<br><br>- General/national<br>- Not specified	Classified based on geographic descriptor reported in study. Where geographic descriptions were lacking, the article was classified as "Not specified"<br>- *Arctic and northern* includes the northern provinces of Canada<br>- *Remote* includes semi-remote<br>- *Island* includes Pacific island studies that do not specify geography
**Population/Setting**	Poplation setting – general context	- Indigenous<br>- Multi-ethnic population<br>- Other stakeholders<br><br>- General/national<br>- Not specified	- *Indigenous* includes both studies involving Indigenous participants, and, studies conducted in Indigenous regions (e.g. reservations, villages)<br>- *Multi-ethcnic* includes ethnically diverse study populations that include Indigenous Peoples<br>- *Other stakeholders* includes studies involving retail workforce (e.g. store managers), public health (e.g. nutritionists and nurses) and others<br>- *General/national* includes studies conducted in regions with a high propondency of Indigenous residents but not exclusive to Indigenous communities and populations
	Indigenous population involved/concerned	- Aboriginal and Torres Strait Islander<br>- Alaska Native<br>- American Indian<br>- Chamorros<br>- First Nation<br>- Métis<br>- Inuit<br>- Māori<br>- Native Hawaiians<br>- Pacific Islander<br><br>- General/national<br>- Not specified	If study involved non-Indigenous participants/regions and other satkeholders, it was classified here based on the concerned Indigenous population group of interest.
**Study design, approach/methods**	Indigenous participation	- Yes<br>- No<br>- Desktop study<br>- Not specified (NS)	Indigenous participation in the research was broadly defined to involve various forms of engagement and participation (e.g. collaboration with Indigenous/community organizations, hiring of local research staff, co-development of project methods). Approval of Indigenous communities/organizations to conduct the research was considered an ethnical prerequisite to the conduct of research in Indigenous communities and was therefore not in and of itself considered participatory in nature.
	Study design	- Qualitative<br>- Quantitative<br>- Mixed and multi-method deisgns	Mixed and mult-method designs included both quantitative and qualitative designs and combinations thereof.
**Focus**	Retail food sector focus	- Supply chain	
		- Workforce	- Retail workforce<br>- Health workforce (e.g. nutritionists, dietitians)
		- Stores	- Number and type of stores<br>- Store access<br>- Store business philosophy, management, and community governance
		- Consumers	- Percpetions of the food supply<br>- Shopping / purchase behaviour

	Improving the market food sector	-	Planning and strategies
		-	Interventions
		-	Policies

Table S7. Key characteristics of the articles included in the review

Reference	Title	Year of publication	Country	Geography [1]	Setting or population [2]	Indigenous People	Study Design	Focus [3]
[7]	A Community-Based, Environmental Chronic Disease Prevention Intervention to Improve Healthy Eating Psychosocial Factors and Behaviors in Indigenous Populations in the Canadian Arctic	2013	Canada	Arctic and Northern	Indigenous	Inuit	QUANT	Improving
[8]	A comparison of dietary estimates from the National Aboriginal and Torres Strait Islander Health Survey to food and beverage purchase data	2017	Australia	Remote	Indigenous	Aboriginal and Torres Strait Islander	QUANT	Consumer
[9]	A Food Store Intervention Trial Improves Caregiver Psychosocial Factors and Children's Dietary Intake in Hawaii	2010	United States	Island	Multi-ethnic	Native Hawaiians; Pacific Islander	QUANT	Improving
[10]	A Food Store-Based Environmental Intervention Is Associated with Reduced BMI and Improved Psychosocial Factors and Food-Related Behaviors on the Navajo Nation	2013	United States	Rural	Indigenous	American Indian	QUANT	Improving
[11]	A Healthy Retail Intervention in Native American Convenience Stores: The THRIVE Community-Based Participatory Research Study	2019	United States	Rural	Indigenous	American Indian	QUANT	Improving
[12]	A Nutrition Environment Measure to Assess Tribal Convenience Stores: The THRIVE Study	2020	United States	Rural	Indigenous	American Indian	QUANT	Food supply
[13]	Addressing food insecurity in a Native American reservation using community-based participatory research	2012	United States	Rural	Indigenous	American Indian	QUAL	Improving
[14]	Addressing the public health burden caused by the nutrition transition through the Healthy Foods North nutrition and lifestyle intervention programme	2010	Canada	Arctic and Northern	Indigenous	Inuit	QUAL	Improving

Reference	Title	Year of publication	Country	Geography [1]	Setting or population [2]	Indigenous People	Study Design	Focus [3]
[15]	An integrated multi-institutional diabetes prevention program improves knowledge and healthy food acquisition in northwestern Ontario First Nations	2008	Canada	Arctic and Northern	Indigenous	First Nation	QUANT	Improving
[16]	Apparent dietary intake in remote aboriginal communities	1994	Australia	Remote	Indigenous	Aboriginal and Torres Strait Islander	QUANT	Consumer
[17]	Associations between Community Environmental-Level Factors and Diet Quality in Geographically Isolated Australian Communities	2019	Australia	Remote	Indigenous	Aboriginal and Torres Strait Islander	QUANT	Consumer
[18]	Attributes of vitamin A- and calcium-rich food items consumed in K'asho Got'ine, Northwest Territories, Canada	2000	Canada	Arctic and Northern	Indigenous	First Nation	MIXED/MULTI METHODS	Consumer
[19]	Availability and Cost of Healthy Foods in a Large American Indian Community in the North-Central United States	2018	United States	Rural	Indigenous	American Indian	QUANT	Food supply
[20]	Availability and price of healthier food choices and association with obesity prevalence in New Zealand Maori	2018	New Zealand	Rural; Urban	Multi-ethnic	Māori	QUANT	Food supply
[21]	Availability and Promotion of Healthful Foods in Stores and Restaurants - Guam, 2015	2017	Guam	Island	Multi-ethnic	General/National	QUANT	Food supply
[22]	Barriers and facilitators to following the Dietary Guidelines for Americans reported by rural, Northern Plains American-Indian children	2015	United States	Rural	Indigenous	American Indian	QUANT	Consumer
[23]	Bush Tucker, Shop Tucker: Production, Consumption, and Diet at an Aboriginal Outstation	2014	Australia	Remote	Indigenous	Aboriginal and Torres Strait Islander	QUANT	Consumer

Reference	Title	Year of publication	Country	Geography [1]	Setting or population [2]	Indigenous People	Study Design	Focus [3]
[24]	Calories are cheap, nutrients are expensive - The challenge of healthy living in Arctic communities	2018	Canada	Arctic and Northern	Indigenous	Inuit	QUANT	Food supply
[25]	Can a community of practice equip public health nutritionists to work with remote retail to improve the food supply	2015	Australia	Remote	Other stakeholders	Aboriginal and Torres Strait Islander	QUAL	Workforce
[26]	Canada's northern food subsidy Nutrition North Canada: a comprehensive program evaluation	2017	Canada	Arctic and Northern	Indigenous	General/National	MIXED/MULTI METHODS	Improving
[27]	Characteristics of the community-level diet of Aboriginal people in remote northern Australia	2013	Australia	Remote	Indigenous	Aboriginal and Torres Strait Islander	QUANT	Consumer
[28]	Characterizing the local food environment and grocery-store decision making among a large American Indian community in the north-central USA: qualitative results from the Healthy Foods Healthy Families Feasibility Study	2019	United States	Rural	Indigenous	American Indian	QUAL	Consumer; Food supply; Workforce
[29]	CHILE: An Evidence-Based Preschool Intervention for Obesity Prevention in Head Start	2013	United States	Rural	Multi-ethnic	American Indian	QUAL	Improving
[30]	CHILE: Outcomes of a group randomized controlled trial of an intervention to prevent obesity in preschool Hispanic and American Indian children	2016	United States	Rural	Multi-ethnic	American Indian	QUANT	Improving
[31]	Collaborating toward improving food security in Nunavut	2013	Canada	Arctic and Northern	Indigenous	Inuit	QUAL	Improving
[32]	Community Development to Feed the Family in Northern Manitoba Communities: Evaluating Food Activities Based on Their Food Sovereignty, Food Security, and Sustainable Livelihood Outcomes	2012	Canada	Arctic and Northern	Indigenous	First Nation	MIXED/MULTI METHODS	Consumer

Reference	Title	Year of publication	Country	Geography [1]	Setting or population [2]	Indigenous People	Study Design	Focus [3]
[33]	Community perspectives on food insecurity and obesity: Focus groups with caregivers of metis and Off-reserve first nations children	2015	Canada	Urban	Indigenous	First Nation; Métis	QUAL	Consumer
[34]	Community-based communication strategies to promote infant iron nutrition in northern Canada	2006	Canada	Arctic and Northern	Indigenous	First Nation	MIXED/MULTI METHODS	Improving
[35]	Contextual determinants of health behaviours in an aboriginal community in Canada: pilot project	2012	Canada	NS	Indigenous	First Nation	QUANT	Consumer; Food supply
[36]	Cost and Affordability of Diets Modelled on Current Eating Patterns and on Dietary Guidelines, for New Zealand Total Population, Maori and Pacific Households	2018	New Zealand	General/National	Multi-ethnic	Māori; Pacific Islander	MIXED/MULTI METHODS	Food supply
[37]	Cross-sectional analysis of a community-based cooperative grocery store intervention in Saskatoon, Canada	2015	Canada	Urban	Multi-ethnic	General/National	QUANT	Improving
[38]	Detection of 12.5% and 25% Salt Reduction in Bread in a Remote Indigenous Australian Community	2016	Australia	Remote	Indigenous	Aboriginal and Torres Strait Islander	QUANT	Improving
[39]	Developing nutrition education resources for a multi-ethnic population in New Zealand	2009	New Zealand	Urban	Multi-ethnic	Māori; Pacific Islander	QUAL	Consumer
[40]	Development of an integrated diabetes prevention program with First Nations in Canada	2006	Canada	Arctic and Northern	Indigenous	First Nation	MIXED/MULTI METHODS	Improving
[41]	Development of the good food planning tool: A food system approach to food security in indigenous Australian remote communities	2015	Australia	Remote	Indigenous	Aboriginal and Torres Strait Islander	QUAL	Improving

Reference	Title	Year of publication	Country	Geography [1]	Setting or population [2]	Indigenous People	Study Design	Focus [3]
[42]	Diabetes and Obesity Associated with Poor Food Environments in American Indian Communities: the Tribal Health and Resilience in Vulnerable Environments (THRIVE) Study	2019	United States	Rural	Multi-ethnic	American Indian	QUANT	Consumer
[43]	Dietary sodium and iodine in remote Indigenous Australian communities: will salt-reduction strategies increase risk of iodine deficiency? A cross-sectional analysis and simulation study	2015	Australia	Remote	Indigenous	Aboriginal and Torres Strait Islander	QUANT	Improving
[44]	Do effects of price discounts and nutrition education on food purchases vary by ethnicity, income and education? Results from a randomised, controlled trial	2011	New Zealand	NS	Multi-ethnic	Māori; Pacific Islander	QUANT	Improving
[45]	Eating in place: Mapping alternative food procurement in Canadian Indigenous communities	2019	Canada	General/National	Indigenous	NS	QUAL	Store
[46]	Economic incentives to promote healthier food purchases: exploring acceptability and key factors for success	2012	New Zealand	Urban	Multi-ethnic	Māori; Pacific Islander	QUAL	Improving
[47]	Effect of 25% Sodium Reduction on Sales of a Top-Selling Bread in Remote Indigenous Australian Community Stores: A Controlled Intervention Trial	2017	Australia	Remote	Indigenous	Aboriginal and Torres Strait Islander	QUANT	Improving
[48]	Effect of a price discount and consumer education strategy on food and beverage purchases in remote Indigenous Australia: a stepped-wedge randomised controlled trial	2017	Australia	Remote	Indigenous	Aboriginal and Torres Strait Islander	QUANT	Improving
[49]	Effects of Health-Related Food Taxes and Subsidies on Mortality from Diet-Related Disease in New Zealand: An Econometric-Epidemiologic Modelling Study	2015	New Zealand	Urban	Multi-ethnic	Māori; Pacific Islander	QUANT	Improving

Reference	Title	Year of publication	Country	Geography [1]	Setting or population [2]	Indigenous People	Study Design	Focus [3]
[50]	Engagement, Recruitment, and Retention in a Trans-Community, Randomized Controlled Trial for the Prevention of Obesity in Rural American Indian and Hispanic Children	2014	United States	Rural	Multi-ethnic	American Indian	QUAL	Improving
[51]	Engaging Tribal Leaders in an American Indian Healthy Eating Project Through Modified Talking Circles	2011	United States	Rural; Urban	Indigenous	American Indian	QUAL	Consumer
[52]	Enhancing Indigenous food sovereignty: A five-year collaborative tribal-university research and extension project in California and Oregon	2019	United States	NS	Indigenous	American Indian	QUAL	Improving
[53]	Evidence for validity of five secondary data sources for enumerating retail food outlets in seven American Indian Communities in North Carolina	2012	United States	Rural; Urban	Indigenous	American Indian	QUANT	Store
[54]	Factors Influencing Food Choice in an Australian Aboriginal Community	2014	Australia	Remote	Indigenous	Aboriginal and Torres Strait Islander	QUAL	Consumer
[55]	Factors related to fruit, vegetable and traditional food consumption which may affect health among Alaska Native People in Western Alaska	2012	United States	Arctic and northern	Indigenous	Alaska Native	QUANT	Food supply
[56]	Feasibility of a novel participatory multi-sector continuous improvement approach to enhance food security in remote Indigenous Australian communities	2017	Australia	Remote	Indigenous	Aboriginal and Torres Strait Islander	MIXED/MULTI METHODS	Improving
[57]	Feasibility study of asset mapping with children: identifying how the community environment shapes activity and food choices in Alexander First Nation	2013	Canada	Rural	Indigenous	First Nation	QUAL	Food supply; Store

Reference	Title	Year of publication	Country	Geography [1]	Setting or population [2]	Indigenous People	Study Design	Focus [3]
[58]	Feeding the family during times of stress: experience and determinants of food insecurity in an Inuit community	2011	Canada	Arctic and northern	Indigenous	Inuit	QUAL	Food supply; Workforce
[59]	Fluctuations in money availability within an income cycle impacts diet quality of remote Indigenous Australians	2017	Australia	Remote	Indigenous	Aboriginal and Torres Strait Islander	QUANT	Consumer
[60]	Food Access and Cost in American Indian Communities in Washington State	2011	United States	NS	Indigenous	American Indian	QUANT	Food supply; Store
[61]	Food acquisition habits, nutrient intakes, and anthropometric data of Havasupai adults	1997	United States	Remote	Indigenous	American Indian	QUANT	Consumer
[62]	Food availability, cost disparity and improvement in relation to accessibility and remoteness in Queensland	2002	Australia	Rural; Remote; Urban	General/National	Aboriginal and Torres Strait Islander	QUANT	Food supply
[63]	Food Environments around American Indian Reservations: A Mixed Methods Study	2016	United States	NS	Indigenous	American Indian	MIXED/MULTI METHODS	Store
[64]	Food expenditure patterns in the Canadian Arctic show cause for concern for obesity and chronic disease	2014	Canada	Arctic and northern	Indigenous	Inuit	QUANT	Food supply
[65]	Food Insecurity among Inuit Women Exacerbated by Socio-economic Stresses and Climate Change	2010	Canada	Arctic and northern	Indigenous	Inuit	QUAL	Food supply
[66]	Food insecurity and household eating patterns among vulnerable American-Indian families: associations with caregiver and food consumption characteristics	2013	United States	NS	Indigenous	NS	QUANT	Consumer

Reference	Title	Year of publication	Country	Geography [1]	Setting or population [2]	Indigenous People	Study Design	Focus [3]
[67]	Food perceptions and dietary behavior of American-Indian children,their caregivers, and educators: Formative assessment findings from pathways	2000	United States	NS	Indigenous	American Indian	QUAL	Consumer
[68]	Food Prices and Consumer Demand: Differences across Income Levels and Ethnic Groups	2013	New Zealand	General/National	Multi-ethnic	Māori	QUANT	Consumer
[69]	Food scarcity, not economic constraint limits consumption in a rural Aboriginal community	2012	Australia	Rural	Indigenous	Aboriginal and Torres Strait Islander	QUANT	Consumer
[70]	Food Security Experiences of Aboriginal and Torres Strait Islander Families with Young Children in An Urban Setting: Influencing Factors and Coping Strategies	2018	Australia	Urban	Indigenous	Aboriginal and Torres Strait Islander	QUAL	Consumer
[71]	Food security in Nunavut, Canada: Barriers and recommendations	2006	Canada	Arctic and northern	Indigenous	Inuit	QUAL	Improving
[72]	Food-purchasing behaviour in an Aboriginal community. 1. Results of a survey	1994	Australia	NS	Indigenous	Aboriginal and Torres Strait Islander	QUANT	Consumer
[73]	Food-purchasing behaviour in an Aboriginal community. 2. Evaluation of an intervention aimed at children	1994	Australia	NS	Indigenous	Aboriginal and Torres Strait Islander	QUANT	Improving
[74]	Food-related environmental, behavioral, and personal factors associated with body mass index among urban, low-income African-American, American Indian, and Caucasian women	2011	United States	Urban	Multi-ethnic	American Indian	QUANT	Consumer
[75]	Formative research and stakeholder participation in intervention development	2005	United States	NS	Indigenous	American Indian	MIXED/MULTI METHODS	Improving

Reference	Title	Year of publication	Country	Geography [1]	Setting or population [2]	Indigenous People	Study Design	Focus [3]
[76]	From targets to ripples: tracing the process of developing a community capacity building appraisal tool with remote Australian indigenous communities to tackle food security	2014	Australia	Remote	Other stakeholders	Aboriginal and Torres Strait Islander	QUAL	Improving
[77]	Geographic factors as determinants of food security: a Western Australian food pricing and quality study	2014	Australia	Rural; Remote; Urban	General/National	Aboriginal and Torres Strait Islander	QUANT	Food supply
[78]	Giving voice to food insecurity in a remote indigenous community in subarctic Ontario, Canada: traditional ways, ways to cope, ways forward	2013	Canada	Arctic and northern	Indigenous	First Nation	QUAL	Improving
[79]	Health and Eating Behavior Differs Between Lean/Normal and Overweight/Obese Low-Income Women Living in Food-Insecure Environments	2013	United States	NS	Multi-ethnic	American Indian	QUAL	Consumer
[80]	Health and Economic Impacts of Eight Different Dietary Salt Reduction Interventions	2015	New Zealand	General/National	Multi-ethnic	Māori	QUANT	Improving
[81]	Health outcomes of a subsidised fruit and vegetable program for Aboriginal children in northern New South Wales	2013	Australia	Rural	Indigenous	Aboriginal and Torres Strait Islander	QUANT	Improving
[82]	Health-Promoting Food Pricing Policies and Decision-Making in Very Remote Aboriginal and Torres Strait Islander Community Stores in Australia	2018	Australia	Remote	Indigenous	Aboriginal and Torres Strait Islander	QUAL	Improving
[83]	Healthful Nutrition of Foods in Navajo Nation Stores: Availability and Pricing	2016	United States	Rural; Remote	Indigenous	American Indian	QUANT	Food supply; Store
[84]	Healthy Choice Rewards: A Feasibility Trial of Incentives to Influence Consumer Food Choices in a Remote Australian Aboriginal Community	2019	Australia	Remote	Indigenous	Aboriginal and Torres Strait Islander	MIXED/MULTI METHODS	Improving

Reference	Title	Year of publication	Country	Geography [1]	Setting or population [2]	Indigenous People	Study Design	Focus [3]
[85]	Healthy Stores Initiative Associated with Produce Purchasing on Navajo Nation	2019	United States	Rural	Indigenous	American Indian	QUANT	Improving
[86]	High Food Insecurity and Its Correlates Among Families Living on a Rural American Indian Reservation	2012	United States	Rural	Indigenous	American Indian	QUANT	Consumer
[87]	Impact of the changing food environment on dietary practices of an Inuit population in Arctic Canada	2010	Canada	Arctic and northern	Indigenous	Inuit	QUAL	Consumer; Workforce
[88]	Improvements in circulating cholesterol, antioxidants, and homocysteine after dietary intervention in an Australian Aboriginal community	2001	Australia	Remote	Indigenous	Aboriginal and Torres Strait Islander	QUANT	Improving
[89]	Inclusion of indigenous and ethnic minority populations in intervention trials: challenges and strategies in a New Zealand supermarket study	2009	New Zealand	General/National	Multi-ethnic	Māori	QUANT	Improving
[90]	Increasing Healthy Food Availability, Purchasing, and Consumption: Lessons Learned from Implementing a Mobile Grocery	2018	United States	Rural	Indigenous	American Indian	QUANT	Improving
[91]	Integrating Formative Assessment and Participatory Research: Building Healthier Communities in the CHILE Project	2010	United States	Rural	Other stakeholders	American Indian	QUAL	Improving
[92]	Measuring dietary-intake in remote Australian Aboriginal communities	1995	Australia	Remote	Indigenous	Aboriginal and Torres Strait Islander	QUANT	Consumer
[93]	Mediators and moderators of nutrition intervention effects in remote Indigenous Australia	2018	Australia	Remote	Indigenous	Aboriginal and Torres Strait Islander	QUANT	Improving

Reference	Title	Year of publication	Country	Geography [1]	Setting or population [2]	Indigenous People	Study Design	Focus [3]
[94]	Modelling the cost differential between healthy and current diets: the New Zealand case study	2018	New Zealand	General/National	Multi-ethnic	Māori	QUANT	Food supply
[95]	Native American obesity: An economic model of the "thrifty gene" theory	2006	United States	NS	Indigenous	American Indian	QUANT	Consumer; Food supply
[96]	Northern food networks: Building collaborative efforts for food security in remote Canadian Aboriginal communities	2013	Canada	Arctic and northern	Other stakeholders	General/National	QUAL	Consumer; Food supply
[97]	Nutrient demand and the allocation of time – evidence from Guam	1993	Guam	Island	General/National	Chamorros	QUANT	Consumer
[98]	Nutrition in remote Aboriginal communities: lessons from Mai Wiru and the Anangu Pitjantjatjara Yankunytjatjara Lands	2016	Australia	Remote	Indigenous	Aboriginal and Torres Strait Islander	MIXED/MULTI METHODS	Improving
[99]	Nutritional impacts of a fruit and vegetable subsidy programme for disadvantaged Australian Aboriginal children	2013	Australia	Rural	Indigenous	Aboriginal and Torres Strait Islander	QUANT	Improving
[100]	OPREVENT (Obesity Prevention and Evaluation of InterVention Effectiveness in NaTive North Americans): Design of a Multilevel, Multicomponent Obesity Intervention for Native American Adults and Households	2019	United States	Rural	Indigenous	American Indian	QUANT	Improving
[101]	Optimisation Modelling to Assess Cost of Dietary Improvement in Remote Aboriginal Australia	2013	Australia	Remote	Indigenous	Aboriginal and Torres Strait Islander	QUANT	Food supply
[102]	Optimisation modelling to improve the diets of First Nations individuals	2019	Canada	General/National	Indigenous	First Nation	QUANT	Food supply

Reference	Title	Year of publication	Country	Geography [1]	Setting or population [2]	Indigenous People	Study Design	Focus [3]
[103]	Para I Famagu'on-Ta: Fruit and Vegetable Intake, Food Store Environment, and Childhood Overweight/Obesity in the Children's Healthy Living Program on Guam	2017	Guam	Island	General/National	Chamorros	QUANT	Food Supply; Store
[104]	Participatory Research for Chronic Disease Prevention in Inuit Communities	2010	Canada	Arctic and northern	Indigenous	Inuit	MIXED/MULTI METHODS	Improving
[105]	Participatory systems approach to health improvement in Australian Aboriginal children	2017	Australia	Remote	Other stakeholders	Aboriginal and Torres Strait Islander	QUAL	Improving
[106]	Prevalence of food insecurity in a Greenlandic community and the importance of social, economic and environmental stressors	2012	Greenland	Arctic and Northern	Indigenous	Inuit	MIXED/MULTI METHODS	Consumer
[107]	Process evaluation of a multi-institutional community-based program for diabetes prevention among First Nations	2008	Canada	Arctic and northern	Indigenous	First Nation	MIXED/MULTI METHODS	Improving
[108]	Process evaluation of a store-based environmental obesity intervention on two American Indian Reservations	2005	United States	NS	Indigenous	American Indian	MIXED/MULTI METHODS	Improving
[109]	Processed foods available in the Pacific Islands	2013	Guam; New Caledonia	Island	General/National	Pacific Islander	QUANT	Food supply
[110]	Psychosocial determinants of food purchasing and preparation in American Indian households	2006	United States	NS	Indigenous	American Indian	QUANT	Consumer
[111]	Qualitative Investigation of Factors Contributing to Effective Nutrition Education for Navajo Families	2008	United States	Rural	Indigenous	American Indian	QUAL	Consumer; Food supply

Reference	Title	Year of publication	Country	Geography [1]	Setting or population [2]	Indigenous People	Study Design	Focus [3]
[112]	Race, Homelessness, and Other Environmental Factors Associated with the Food-Purchasing Behavior of Low-Income Women	2010	United States	Urban	Multi-ethnic	American Indian	QUANT	Consumer
[113]	Reframing food security by and for Native American communities: a case study among tribes in the Klamath River basin of Oregon and California	2019	United States	Rural; NS	Indigenous	American Indian	QUAL	Store; Food supply; Improving
[114]	Retail food environments, shopping experiences, First Nations and the provincial Norths	2017	Canada	Arctic and northern	Indigenous	First Nation	QUANT	Store; Consumer
[115]	Review of the nutrition policy of the Arnhem Land Progress Association	1996	Australia	Remote	Indigenous	Aboriginal and Torres Strait Islander	MIXED/MULTI METHODS	Improving
[116]	Seeking Indigenous food sovereignty: origins of and responses to the food crisis in northern Manitoba, Canada	2013	Canada	Arctic and northern	Indigenous	First Nation; Métis	QUAL	Food supply; Improving
[117]	Sodium in Store and Restaurant Food Environments - Guam, 2015	2016	Guam	Island	General/National	General/National	QUANT	Workforce
[118]	Store turnover as a predictor of food and beverage provider turnover and associated dietary intake estimates in very remote Indigenous communities	2016	Australia	Remote	Indigenous	Aboriginal and Torres Strait Islander	QUANT	Consumer
[119]	Strategies to promote healthier food purchases: a pilot supermarket intervention study	2007	New Zealand	Urban	Multi-ethnic	Māori	QUANT	Improving
[120]	Strengthening food systems with remote Indigenous Australians: stakeholders' perspectives	2018	Australia	Remote	Other stakeholders	Aboriginal and Torres Strait Islander	QUAL	Consumer

Reference	Title	Year of publication	Country	Geography [1]	Setting or population [2]	Indigenous People	Study Design	Focus [3]
[121]	Sugar-sweetened beverages in Pacific Island countries and territories: problems and solutions	2014	French Polynesia; Guam	Island	General/National	General/National	QUAL	Improving
[122]	Supporting healthy drink choices in remote Aboriginal and Torres Strait Islander communities: a community-led supportive environment approach	2019	Australia	Remote	Indigenous	Aboriginal and Torres Strait Islander	QUANT	Improving
[123]	Tackling 'wicked' health promotion problems: a New Zealand case study	2013	New Zealand	General/National	Other stakeholders	Māori; Pacific Islander	MIXED/MULTI METHODS	Improving
[124]	Testing the Price of Healthy and Current Diets in Remote Aboriginal Communities to Improve Food Security: Development of the Aboriginal and Torres Strait Islander Healthy Diets ASAP (Australian Standardised Affordability and Pricing) Methods	2018	Australia	Remote	Indigenous	Aboriginal and Torres Strait Islander	QUANT	Food supply
[125]	The association between neighborhood socioeconomic status and exposure to supermarkets and fast food outlets	2008	Canada	Urban	Multi-ethnic	General/National	QUANT	Store
[126]	The Community Food Environment and Food Insecurity in Sioux Lookout, Ontario: Understanding the Relationships between Food, Health, and Place	2019	Canada	Arctic and northern	Multi-ethnic	First Nation	MIXED/MULTI METHODS	Consumer
[127]	The comparative cost of food and beverages at remote Indigenous communities, Northern Territory, Australia	2016	Australia	Remote	Indigenous	Aboriginal and Torres Strait Islander	QUANT	Food supply
[128]	The complexities of selling fruits and vegetables in remote Navajo Nation retail outlets: perspectives from owners and managers of small stores	2020	United States	Remote	Indigenous	American Indian	QUAL	Workforce

Reference	Title	Year of publication	Country	Geography [1]	Setting or population [2]	Indigenous People	Study Design	Focus [3]
[129]	The contemporary food supply of three northern Manitoba Cree communities	1997	Canada	Arctic and northern	Indigenous	First Nation	MIXED/MULTI METHODS	Food supply
[130]	The cost-effectiveness of a 20% price discount on fruit, vegetables, diet drinks and water, trialled in remote Australia to improve Indigenous health	2018	Australia	Remote	Indigenous	Aboriginal and Torres Strait Islander	QUANT	Improving
[131]	The economic feasibility of price discounts to improve diet in Australian Aboriginal remote communities	2016	Australia	Remote	Indigenous	Aboriginal and Torres Strait Islander	QUANT	Improving
[132]	The food security of Inuit women in Arviat, Nunavut: the role of socio-economic factors and climate change	2015	Canada	Arctic and northern	Indigenous	Inuit	QUAL	Food supply
[133]	The Grocery Store Food Environment in Northern Greenland and Its Implications for the Health of Reproductive Age Women	2018	Greenland	Arctic and northern	Indigenous	Inuit	MIXED/MULTI METHODS	Food supply; Workforce; Consumer
[134]	The role of energy cost in food choices for an Aboriginal population in northern Australia	2009	Australia	Remote	Indigenous	Aboriginal and Torres Strait Islander	QUANT	Consumer
[135]	The Sioux Lookout Diabetes Program: diabetes prevention and management in northwestern Ontario	1998	Canada	Arctic and Northern	Indigenous	First Nation	QUAL	Improving
[136]	Through the lens of our cameras: children's lived experience with food security in a Canadian Indigenous community	2015	Canada	Rural	Indigenous	First Nation	QUAL	Consumer
[137]	Toward a community impact assessment for food policy councils: Identifying potential impact domains	2018	Canada; United States	General/National	Other stakeholders; Indigenous	General/National	QUAL	Improving

Reference	Title	Year of publication	Country	Geography [1]	Setting or population [2]	Indigenous People	Study Design	Focus [3]
[138]	Traditional and market food access in Arctic Canada is affected by economic factors	2006	Canada	Arctic and northern	Indigenous	First Nation; Métis; Inuit	QUANT	Food supply
[139]	Understanding food security issues in remote Western Australian Indigenous communities	2014	Australia	Remote	Other stakeholders	Aboriginal and Torres Strait Islander	QUAL	Workforce; Improving
[140]	Understanding Local Food Behaviour and Food Security in Rural First Nation Communities: Implications for Food Policy	2012	Canada	Rural	Indigenous	First Nation	QUANT	Consumer
[141]	Use of electronic sales data to tailor nutrition education resources for an ethnically diverse population	2010	New Zealand	NS	Multi-ethnic	Māori; Pacific Islander	QUANT	Consumer
[142]	Use of point-of-sale data to assess food and nutrient quality in remote stores	2012	Australia	Remote	Indigenous	Aboriginal and Torres Strait Islander	QUANT	Consumer
[143]	Using a SWOT analysis to inform healthy eating and physical activity strategies for a remote First Nations community in Canada	2012	Canada	Arctic and Northern	Indigenous	First Nation	QUAL	Improving

[1] Geography as self-defined in the study – accordingly definitions or rurality and remoteness may differ between studies
[2] Indigenous settings includes studies conducted in Indigenous regions (e.g. communities, reservations, nations) and/or involving Indigenous study populations. Multi-ethnic settings includes studies conducted in ethnically diverse regions and/or study populations, including Indigenous study participants
[3] Definitions of areas of thematic focus are summarized in the data abstraction scheme - Table S6

References Supplemental Material

1. Cisneros-Montemayor, A.M.; Pauly, D.; Weatherdon, L.V.; Ota, Y. A Global Estimate of Seafood Consumption by Coastal Indigenous Peoples. PLoS ONE 2016, 11, doi:10.1371/journal.pone.0166681.
2. World Bank How does the World Bank classify countries? Available online: https://datahelpdesk.worldbank.org/knowledgebase/articles/378834-how-does-the-world-bank-classify-countries (accessed on Sep 20, 2019).
3. UNPFII Indigenous Peoples, Indigenous Voices. Fact Sheet. Who are indigenous peoples? Available online: https://www.un.org/esa/socdev/unpfii/documents/5session_factsheet1.pdf (accessed on May 25, 2020).
4. World Directory of Minorities and Indigenous Peoples - Brunei Darussalam : Dusun, Murut, Kedayan, Iban, Tutong, Penan. 2008, 1–4.
5. eHRAF World Cultures Available online: https://ehrafworldcultures.yale.edu/ehrafe/ (accessed on Oct 1, 2019).
6. Anderson, I.; Robson, B.; Connolly, M.; Al-Yaman, F.; Bjertness, E.; King, A.; Tynan, M.; Madden, R.; Bang, A.; Coimbra, C.E.A.; et al. Indigenous and tribal peoples' health (The Lancet–Lowitja Institute Global Collaboration): a population study. Lancet 2016, 388, 131–157, doi:10.1016/s0140-6736(16)00345-7.
7. Mead, E.; Gittelsohn, J.; Roache, C.; Corriveau, A.; Sharma, S. A Community-Based, Environmental Chronic Disease Prevention Intervention to Improve Healthy Eating Psychosocial Factors and Behaviors in Indigenous Populations in the Canadian Arctic. Health Education & Behavior 2013, 40, 592–602, doi:10.1177/1090198112467793.
8. McMahon, E.; Wycherley, T.P.; O'Dea, K.; Brimblecombe, J.K. A comparison of dietary estimates from the National Aboriginal and Torres Strait Islander Health Survey to food and beverage purchase data. Australian and New Zealand Journal of Public Health 2017, 41, 598–603, doi:10.1111/1753-6405.12718.
9. Gittelsohn, J.; Vijayadeva, V.; Davison, N.; Ramirez, V.; Cheung, L.W.K.; Murphy, S.; Novotny, R. A Food Store Intervention Trial Improves Caregiver Psychosocial Factors and Children's Dietary Intake in Hawaii. Obesity 2010, 18, S84–S90, doi:10.1038/oby.2009.436.
10. Gittelsohn, J.; Kim, E.M.; He, S.; Pardilla, M. A Food Store-Based Environmental Intervention Is Associated with Reduced BMI and Improved Psychosocial Factors and Food-Related Behaviors on the Navajo Nation. The Journal of Nutrition 2013, 143, 1494–1500, doi:10.3945/jn.112.165266.
11. Jernigan, V.B.B.; Salvatore, A.L.; Williams, M.; Wetherill, M.; Taniguchi, T.; Jacob, T.; Cannady, T.; Grammar, M.; Standridge, J.; Fox, J.; et al. A Healthy Retail Intervention in Native American Convenience Stores: The THRIVE Community-Based Participatory Research Study. Am J Public Health 2019, 109, 132–139, doi:10.2105/ajph.2018.304749.
12. Wetherill, M.S.; Williams, M.B.; Taniguchi, T.; Salvatore, A.L.; Jacob, T.; Cannady, T.; Grammar, M.; Standridge, J.; Fox, J.; Spiegel, J.; et al. A Nutrition Environment Measure to Assess Tribal Convenience Stores: The THRIVE Study: Health Promotion Practice 2020, 21, 410–420, doi:10.1177/1524839918800968.
13. Jernigan, V.B.B.; Salvatore, A.L.; Styne, D.M.; Winkleby, M. Addressing food insecurity in a Native American reservation using community-based participatory research. Health Education Research 2012, 27, 645–655, doi:10.1093/her/cyr089.
14. Sharma, S.; Gittelsohn, J.; Rosol, R.; Beck, L. Addressing the public health burden caused by the nutrition transition through the Healthy Foods North nutrition and lifestyle intervention programme. Journal of Human Nutrition and Dietetics 2010, 23, 120–127, doi:10.1111/j.1365-277x.2010.01107.x.

15. Ho, L.S.; Gittelsohn, J.; Rimal, R.; Treuth, M.S.; Sharma, S.; Rosecrans, A.; Harris, S.B. An Integrated Multi-Institutional Diabetes Prevention Program Improves Knowledge and Healthy Food Acquisition in Northwestern Ontario First Nations: Health Education & Behavior 2008, 35, 561–573, doi:10.1177/1090198108315367.
16. Lee, A.J.; O'Dea, K.; Mathews, J.D. Apparent dietary intake in remote Aboriginal communities. Australian and New Zealand Journal of Public Health 1994, 18, 190–197, doi:10.1111/j.1753-6405.1994.tb00224.x.
17. Wycherley, T.P.; Pols, J.C. van der; Daniel, M.; Howard, N.J.; O'Dea, K.; Brimblecombe, J.K. Associations between Community Environmental-Level Factors and Diet Quality in Geographically Isolated Australian Communities. International Journal of Environmental Research and Public Health 2019, 16, 1943, doi:10.3390/ijerph16111943.
18. Simoneau, N.; Receveur, O. Attributes of Vitamin A- and Calcium-Rich Food Items Consumed in K'asho Got'ine, Northwest Territories, Canada. Journal of Nutrition Education 2000, 32, 84–93, doi:10.1016/s0022-3182(00)70524-3.
19. Fretts, A.M.; Huber, C.; Best, L.G.; O'Leary, M.; LeBeau, L.; Howard, B.V.; Siscovick, D.S.; Beresford, S.A. Availability and Cost of Healthy Foods in a Large American Indian Community in the North-Central United States. Preventing Chronic Disease 2018, 15, 170302–5, doi:10.5888/pcd15.170302.
20. Jani, R.; Rush, E.; Crook, N.; Simmons, D. Availability and price of healthier food choices and association with obesity prevalence in New Zealand Māori. Asia Pac J Clin Nutr 2018, 27, 1357–1365, doi:10.6133/apjcn.201811_27(6).0023.
21. Lundeen, E.A.; VanFrank, B.K.; Jackson, S.L.; Harmon, B.; Uncangco, A.; Luces, P.; Dooyema, C.; Park, S. Availability and Promotion of Healthful Foods in Stores and Restaurants — Guam, 2015. Preventing Chronic Disease 2017, 14, 160528–6, doi:10.5888/pcd14.160528.
22. Jahns, L.; McDonald, L.; Wadsworth, A.; Morin, C.; Liu, Y.; Nicklas, T. Barriers and facilitators to following the Dietary Guidelines for Americans reported by rural, Northern Plains American-Indian children. Public Health Nutrition 2015, 18, 482–489, doi:10.1017/s136898001400041x.
23. Scelza, B.; Bird, D.W.; Bird, R.B. Bush Tucker, Shop Tucker: Production, Consumption, and Diet at an Aboriginal Outstation. Ecology of Food and Nutrition 2014, 53, 98–117, doi:10.1080/03670244.2013.772513.
24. Kenny, T.A.; Fillion, M.; MacLean, J.; Wesche, S.D.; Chan, H.M. Calories are cheap, nutrients are expensive – The challenge of healthy living in Arctic communities. Food Policy 2018, 80, 39–54, doi:10.1016/j.foodpol.2018.08.006.
25. Holden, S.; Ferguson, M.M.; Brimblecombe, J.K.; Palermo, C. Can a community of practice equip public health nutritionists to work with remote retail to improve the food supply? Rural and Remote Health 2015, 15, 1–11.
26. Galloway, T. Canada's northern food subsidy Nutrition North Canada: a comprehensive program evaluation. International Journal of Circumpolar Health 2017, 76, 1279451, doi:10.1080/22423982.2017.1279451.
27. Brimblecombe, J.K.; Ferguson, M.M.; Liberato, S.C.; O'Dea, K. Characteristics of the community-level diet of Aboriginal people in remote northern Australia. Med J Aust 2013, 198, 380–384, doi:10.5694/mja12.11407.
28. Brown, M.C.; Shrestha, U.; Huber, C.; Best, L.G.; O'Leary, M.; Howard, B.; Beresford, S.; Fretts, A.M. Characterizing the local food environment and grocery-store decision making among a large American Indian community in the north-central USA: qualitative results from the Healthy Foods Healthy Families Feasibility Study. Public Health Nutrition 2019, 22, 2653–2661, doi:10.1017/s1368980019001095.

29. Davis, S.M.; Sanders, S.G.; FitzGerald, C.A.; Keane, P.C.; Canaca, G.F.; Rector, R.V. CHILE: An Evidence-Based Preschool Intervention for Obesity Prevention in Head Start. Journal of School … 2013, 83, 223–229, doi:10.1111/josh.12018.
30. Davis, S.M.; Myers, O.B.; Cruz, T.H.; Morshed, A.B.; Canaca, G.F.; Keane, P.C.; O'Donald, E.R. CHILE: Outcomes of a group randomized controlled trial of an intervention to prevent obesity in preschool Hispanic and American Indian children. Preventive Medicine 2016, 89, 162–168, doi:10.1016/j.ypmed.2016.05.018.
31. Wakegijig, J.; Osborne, G.; Statham, S.; Issaluk, M.D. Collaborating toward improving food security in Nunavut. International Journal of Circumpolar Health 2013, 72, doi:10.3402/ijch.v72i0.21201.
32. Thompson, S.; Kamal, A.G.; Alam, M.A.; Wiebe, J. Community Development to Feed the Family in Northern Manitoba Communities: Evaluating Food Activities based on Their Food Sovereignty, Food Security, and Sustainable Livelihood Outcomes. Can J Nonprofit Soc Econ Res 2012, 3, 43 – 66, doi:10.22230/cjnser.2012v3n2a121.
33. Bhawra, J.; Cooke, M.J.; Hanning, R.; Wilk, P.; Gonneville, S.L.H. Community perspectives on food insecurity and obesity: Focus groups with caregivers of Métis and Off-reserve First Nations children. International Journal for Equity in Health 2015, 14, 1–10, doi:10.1186/s12939-015-0232-5.
34. Verrall, T.; Napash, L.; Leclerc, L.; Mercure, S.; Gray-Donald, K. Community-based communication strategies to promote infant iron nutrition in northern Canada. Int J Circumpol Heal 2006, 65, 65–78, doi:10.3402/ijch.v65i1.17892.
35. Joseph, P.; Davis, A.D.; Miller, R.; Hill, K.; McCarthy, H.; Banerjee, A.; Chow, C.; Mente, A.; Anand, S.S. Contextual determinants of health behaviours in an aboriginal community in Canada: pilot project. BMC public health 2012, 12, 1–8, doi:10.1186/1471-2458-12-952.
36. Mackay, S.; Buch, T.; Vandevijvere, S.; Goodwin, R.; Korohina, E.; Funaki-Tahifote, M.; Lee, A.J.; Swinburn, B. Cost and Affordability of Diets Modelled on Current Eating Patterns and on Dietary Guidelines, for New Zealand Total Population, Māori and Pacific Households. International Journal of Environmental Research and Public Health 2018, 15, 1255, doi:10.3390/ijerph15061255.
37. Lotoski, L.C.; Engler-Stringer, R.; Muhajarine, N. Cross-sectional analysis of a community-based cooperative grocery store intervention in Saskatoon, Canada. Canadian Journal of Public Health 2015, 106, e147–e153, doi:10.17269/cjph.106.4710.
38. McMahon, E.; Clarke, R.; Jaenke, R.; Brimblecombe, J.K. Detection of 12.5% and 25% Salt Reduction in Bread in a Remote Indigenous Australian Community. Nutrients 2016, 8, 169, doi:10.3390/nu8030169.
39. Eyles, H.; Mhurchu, C.N.; Wharemate, L.; Funaki-Tahifote, M.; Lanumata, T.; Rodgers, A. Developing nutrition education resources for a multi-ethnic population in New Zealand. Health Education Research 2009, 24, 558–574, doi:10.1093/her/cyn057.
40. Ho, L.S. Development of an integrated diabetes prevention program with First Nations in Canada. Health Promotion International 2006, 21, 88–97, doi:10.1093/heapro/dak003.
41. Brimblecombe, J.K.; Boogaard, C.V.D.; Wood, B.; Liberato, S.C.; Brown, J.; Barnes, A.; Rogers, A.; Coveney, J.; Ritchie, J.; Bailie, R. Development of the good food planning tool: A food system approach to food security in indigenous Australian remote communities. Health & Place 2015, 34, 54–62, doi:10.1016/j.healthplace.2015.03.006.
42. Love, C.V.; Taniguchi, T.E.; Williams, M.B.; Noonan, C.J.; Wetherill, M.S.; Salvatore, A.L.; Jacob, T.; Cannady, T.K.; Standridge, J.; Spiegel, J.; et al. Diabetes and Obesity Associated with Poor Food Environments in American Indian communities: the THRIVE study. Current Developments in Nutrition 2019, doi:10.1093/cdn/nzy099.
43. McMahon, E.; Webster, J.; O'Dea, K.; Brimblecombe, J.K. Dietary sodium and iodine in remote Indigenous Australian communities: will salt-reduction strategies increase risk of

iodine deficiency? A cross-sectional analysis and simulation study. BMC public health 2015, 15, 1–9, doi:10.1186/s12889-015-2686-1.
44. Blakely, T.; Mhurchu, C.N.; Jiang, Y.; Matoe, L.; Funaki-Tahifote, M.; Eyles, H.C.; Foster, R.H.; McKenzie, S.; Rodgers, A. Do effects of price discounts and nutrition education on food purchases vary by ethnicity, income and education? Results from a randomised, controlled trial. Journal of Epidemiology and Community Health 2011, 65, 902–908, doi:10.1136/jech.2010.118588.
45. Sumner, J.; Tarhan, M.D.; McMurtry, J.J. Eating in Place: Mapping Alternative Food Procurement in Canadian Indigenous Communities. Journal of Agriculture, Food Systems, and Community Development 2019, 9, 239–250, doi:10.5304/jafscd.2019.09b.016.
46. Mhurchu, C.N.; Eyles, H.; Dixon, R.; Matoe, L.; Teevale, T.; Meagher-Lundberg, P. Economic incentives to promote healthier food purchases: exploring acceptability and key factors for success. Health Promotion International 2012, 27, 331–341, doi:10.1093/heapro/dar042.
47. McMahon, E.; Webster, J.; Brimblecombe, J.
Effect of 25% Sodium Reduction on Sales of a Top-
Selling Bread in Remote Indigenous Australian Community Stores: A Controlled Interventi
on Trial. Nutrients 2017, 9, 214, doi:10.3390/nu9030214.
48. Brimblecombe, J.K.; Ferguson, M.M.; Chatfield, M.D.; Liberato, S.C.; Gunther, A.; Ball, K.; Moodie, M.; Miles, E.; Magnus, A.; Mhurchu, C.N.; et al. Effect of a price discount and consumer education strategy on food and beverage purchases in remote Indigenous Australia: a stepped-wedge randomised controlled trial. The Lancet Public Health 2017, 2, e82–e95, doi:10.1016/s2468-2667(16)30043-3.
49. Mhurchu, C.N.; Eyles, H.; Genc, M.; Scarborough, P.; Rayner, M.; Mizdrak, A.; Nnoaham, K.; Blakely, T. Effects of Health-Related Food Taxes and Subsidies on Mortality from Diet-Related Disease in New Zealand: An Econometric-Epidemiologic Modelling Study. PLoS ONE 2015, 10, e0128477, doi:10.1371/journal.pone.0128477.
50. Cruz, T.H.; Davis, S.M.; FitzGerald, C.A.; primary, G.C.T. journal of Engagement, recruitment, and retention in a trans-community, randomized controlled trial for the prevention of obesity in rural American Indian and Hispanic children. Springer 2014, doi:10.1007/s10935-014-0340-9.pdf.
51. Fleischhacker, S.; Vu, M.; Ries, A.; McPhail, A. Engaging Tribal Leaders in an American Indian Healthy Eating Project Through Modified Talking Circles. Family & Community Health 2011, 34, 202–210, doi:10.1097/fch.0b013e31821960bb.
52. Sowerwine, J.; Sarna-Wojcicki, D.; Mucioki, M.; Hillman, L.; Lake, F.; Friedman, E. Enhancing Food Sovereignty: J Agric Food Syst Community Dev 2019, 9, 1–24, doi:10.5304/jafscd.2019.09b.013.
53. Fleischhacker, S.E.; Rodriguez, D.A.; Evenson, K.R.; Henley, A.; Gizlice, Z.; Soto, D.; Ramachandran, G. Evidence for validity of five secondary data sources for enumerating retail food outlets in seven American Indian Communities in North Carolina. International Journal of Behavioral Nutrition and Physical Activity 2012, 9, 1–14, doi:10.1186/1479-5868-9-137.
54. Brimblecombe, J.K.; Maypilama, E.; Colles, S.; Scarlett, M.; Dhurrkay, J.G.; Ritchie, J.; O'Dea, K. Factors Influencing Food Choice in an Australian Aboriginal Community. Qualitative Health Research 2014, 24, 387–400, doi:10.1177/1049732314521901.
55. Johnson, J.S.; Nobmann, E.D.; Asay, E. Factors related to fruit, vegetable and traditional food consumption which may affect health among Alaska Native People in Western Alaska. Int J Circumpol Heal 2012, 71, 17345, doi:10.3402/ijch.v71i0.17345.
56. Brimblecombe, J.K.; Bailie, R.; Boogaard, C. van den; Wood, B.; Liberato, S.C.; Ferguson, M.M.; Coveney, J.; Jaenke, R.; Ritchie, J. Feasibility of a novel participatory multi-sector continuous improvement approach to enhance food security in remote

Indigenous Australian communities. SSM - Population Health 2017, 3, 566–576, doi:10.1016/j.ssmph.2017.06.002.
57. Fehderau, D.D.; Willows, N.D.; Ball, G.D.; Holt, N.L. Feasibility study of asset mapping with children: Identifying how the community environment shapes activity and food choices in Alexander First Nation. 2013, doi:10.7939/r39p2wk8j.
58. Ford, J.D.; Beaumier, M.C. Feeding the family during times of stress: experience and determinants of food insecurity in an Inuit community. The Geographical Journal 2011, 177, 44–61, doi:10.1111/j.1475-4959.2010.00374.x.
59. Wycherley, T.P.; Pekarsky, B.A.; Ferguson, M.M.; O'Dea, K.; Brimblecombe, J.K. Fluctuations in money availability within an income cycle impacts diet quality of remote Indigenous Australians. Public Health Nutrition 2017, 80, 1–10, doi:10.1017/s1368980016003360.
60. O'Connell, M.; Buchwald, D.S.; Duncan, G.E. Food Access and Cost in American Indian Communities in Washington State. Journal of the American Dietetic Association 2011, 111, 1375–1379, doi:10.1016/j.jada.2011.06.002.
61. Vaughan, L.A.; Benyshek, D.C.; Martin, J.F. Food Acquisition Habits, Nutrient Intakes, and Anthropometric Data of Havasupai Adults. J Am Diet Assoc 1997, 97, 1275–1282, doi:10.1016/s0002-8223(97)00305-2.
62. Lee, A.J.; Darcy, A.M.; Leonard, D.; Groos, A.D. Food availability, cost disparity and improvement in relation to accessibility and remoteness in Queensland. Australian and New Zealand Journal of Public Health 2002, 26, 266–272, doi:10.1111/j.1467-842x.2002.tb00164.x.
63. Chodur, G.M.; Shen, Y.; Kodish, S.; Oddo, V.M.; Antiporta, D.A.; Jock, B.; Jones-Smith, J.C. Food Environments around American Indian Reservations: A Mixed Methods Study. Plos One 2016, 11, e0161132, doi:10.1371/journal.pone.0161132.
64. Pakseresht, M.; Lang, R.; Rittmueller, S.; Roache, C.; Sheehy, T.; Batal, M.; Corriveau, A.; Sharma, S. Food expenditure patterns in the Canadian Arctic show cause for concern for obesity and chronic disease. International Journal of Behavioral Nutrition and Physical Activity 2014, 11, 51.
65. Beaumier, M.C.; Ford, J.D. Food Insecurity among Inuit Women Exacerbated by Socio-Economic Stresses and Climate Change. Canadian Journal of Public Health 2010, 101, 196–201, doi:10.1007/bf03404373.
66. Mullany, B.; Neault, N.; Tsingine, D.; Powers, J.; Lovato, V.; Clitso, L.; Massey, S.; Talgo, A.; Speakman, K.; Barlow, A. Food insecurity and household eating patterns among vulnerable American-Indian families: associations with caregiver and food consumption characteristics. Public Health Nutrition 2013, 16, 752–760, doi:10.1017/s136898001200300x.
67. Gittelsohn, J.; Toporoff, E.G.; Story, M.; Evans, M.; Anliker, J.; Davis, S.; Sharma, A.; White, J. Food Perceptions and Dietary Behavior of American-Indian Children, Their Caregivers, and Educators: Formative Assessment Findings from Pathways. Journal of Nutrition Education 2000, 32, 2–13, doi:10.1016/s0022-3182(00)70504-8.
68. Mhurchu, C.N.; Eyles, H.; Schilling, C.; Yang, Q.; Kaye-Blake, W.; Genc, M.; Blakely, T. Food Prices and Consumer Demand: Differences across Income Levels and Ethnic Groups. PLoS ONE 2013, 8, e75934, doi:10.1371/journal.pone.0075934.
69. Scelza, B. Food scarcity, not economic constraint limits consumption in a rural Aboriginal community. Australian Journal of Rural Health 2012, 20, 108–112, doi:10.1111/j.1440-1584.2012.01270.x.
70. McCarthy, L.; Chang, A.; Brimblecombe, J.K. Food Security Experiences of Aboriginal and Torres Strait Islander Families with Young Children in An Urban Setting: Influencing Factors and Coping Strategies. International Journal of Environmental Research and Public Health 2018, 15, 2649, doi:10.3390/ijerph15122649.

71. Chan, H.M.; Fediuk, K.; Hamilton, S.; Rostas, L.; Caughey, A.; Kuhnlein, H.V.; Egeland, G.M.; Loring, E. Food security in Nunavut, Canada: Barriers and recommendations. International Journal of Circumpolar Health 2006, 65, 416–431, doi:10.3402/ijch.v65i5.18132.
72. Rowse, T.; Scrimgeour, D.; Knight, S.; Thomas, D. Food-purchasing behaviour in an Aboriginal community. 1. Results of a survey. Australian and New Zealand Journal of Public Health 1994, 18, 63–67, doi:10.1111/j.1753-6405.1994.tb00197.x.
73. Scrimgeour, D.; Rowse, T.; Knight, S. Food-purchasing behaviour in an Aboriginal community. 2. Evaluation of an intervention aimed at children. Australian and New Zealand Journal of Public Health 1994, 18, 67–70, doi:10.1111/j.1753-6405.1994.tb00198.x.
74. Dammann, K.W.; Smith, C. Food-Related Environmental, Behavioral, and Personal Factors Associated with Body Mass Index among Urban, Low-Income African-American, American Indian, and Caucasian Women. American Journal of Health Promotion 2011, 25, e1–e10, doi:10.4278/ajhp.091222-quan-397.
75. Vastine, A.; Gittelsohn, J.; Ethelbah, B.; Anliker, J.; Caballero, B. Formative Research and Stakeholder Participation in Intervention Development. American journal of epidemiology 2005, 29, 57–69, doi:10.5993/ajhb.29.1.5.
76. Brimblecombe, J.K.; Boogaard, C.V.D.; Ritchie, J.; Bailie, R.; Coveney, J.; Liberato, S. From targets to ripples: tracing the process of developing a community capacity building appraisal tool with remote Australian indigenous communities to tackle food security. BMC public health 2014, 14, doi:10.1186/1471-2458-14-914.
77. Pollard, C. Geographic factors as determinants of food security: a Western Australian food pricing and quality study. 2014, 1–11, doi:10.6133/apjcn.2014.23.4.12.
78. Skinner, K.; Hanning, R.M.; Desjardins, E.; Tsuji, L.J. Giving voice to food insecurity in a remote indigenous community in subarctic Ontario, Canada: traditional ways, ways to cope, ways forward. BMC public health 2013, 13, 1–13, doi:10.1186/1471-2458-13-427.
79. Dressler, H.; Smith, C. Health and Eating Behavior Differs between Lean/Normal and Overweight/Obese Low-Income Women Living in Food-Insecure Environments: American Journal of Health Promotion 2013, 27, 358–365, doi:10.4278/ajhp.120119-qual-55.
80. Nghiem, N.; Blakely, T.; Cobiac, L.J.; Pearson, A.L.; Wilson, N. Health and Economic Impacts of Eight Different Dietary Salt Reduction Interventions. PLoS ONE 2015, 10, e0123915, doi:10.1371/journal.pone.0123915.
81. Black, A.P.; Vally, H.; Morris, P.S.; Daniel, M.; Esterman, A.J.; Smith, F.E.; O'Dea, K. Health outcomes of a subsidised fruit and vegetable program for Aboriginal children in northern New South Wales. The Medical journal of Australia 2013, 199, 46–50, doi:10.5694/mja13.10445.
82. Ferguson, M.M.; O'Dea, K.; Altman, J.; Moodie, M.; Brimblecombe, J.K. Health-Promoting Food Pricing Policies and Decision-Making in Very Remote Aboriginal and Torres Strait Islander Community Stores in Australia. International Journal of Environmental Research and Public Health 2018, 15, 2908–14, doi:10.3390/ijerph15122908.
83. Kumar, G.; Jim-Martin, S.; Piltch, E.M.; Onufrak, S.; McNeil, C.; Adams, L.; Williams, N.; Blanck, H.M.; Curley, L. Healthful Nutrition of Foods in Navajo Nation Stores: Availability and Pricing. American Journal of Health Promotion 2016, 30, 501–510, doi:10.4278/ajhp.140821-quan-422.
84. Brown, C.; Laws, C.; Leonard, D.; Campbell, S.; Merone, L.; Hammond, M.; Thompson, K.; Canuto, K.; Brimblecombe, J. Healthy Choice Rewards: A Feasibility Trial of Incentives to Influence Consumer Food Choices in a Remote Australian Aboriginal Community. Int J Environ Res Pu 2019, 16, 112, doi:10.3390/ijerph16010112.

85. MacKenzie, O.W.; George, C.V.; Pérez-Escamilla, R.; Lasky-Fink, J.; Piltch, E.M.; Sandman, S.M.; Clark, C.; Avalos, Q.J.; Carroll, D.S.; Wilmot, T.M.; et al. Healthy Stores Initiative Associated with Produce Purchasing on Navajo Nation. Current Developments in Nutrition 2019, 3, e80973, doi:10.1093/cdn/nzz125.
86. Bauer, K.W.; Widome, R.; Himes, J.H.; Smyth, M.; Rock, B.H.; Hannan, P.J.; Story, M. High Food Insecurity and Its Correlates Among Families Living on a Rural American Indian Reservation. Am J Public Health 2012, 102, 1346–1352, doi:10.2105/ajph.2011.300522.
87. Mead, E.; Gittelsohn, J.; Kratzmann, M.; Roache, C.; Sharma, S. Impact of the changing food environment on dietary practices of an Inuit population in Arctic Canada. Journal of Human Nutrition and Dietetics 2010, 23, 18–26, doi:10.1111/j.1365-277x.2010.01102.x.
88. Rowley, K.G.; Su, Q.; Cincotta, M.; Skinner, M.; Skinner, K.; Pindan, B.; White, G.A.; O'Dea, K. Improvements in circulating cholesterol, antioxidants, and homocysteine after dietary intervention in an Australian Aboriginal community. American Journal of Clinical Nutrition 2001, 74, 442–448, doi:10.1093/ajcn/74.4.442.
89. Mhurchu, C.N.; Blakely, T.; Funaki-Tahifote, M.; McKerchar, C.; Wilton, J.; Chua, S.; Jiang, Y. Inclusion of indigenous and ethnic minority populations in intervention trials: challenges and strategies in a New Zealand supermarket study. J Epidemiol Commun H 2009, 63, 850, doi:10.1136/jech.2008.081109.
90. Cueva, K.; Lovato, V.; Nieto, T.; Neault, N.; Barlow, A.; Speakman, K. Increasing Healthy Food Availability, Purchasing, and Consumption: Lessons Learned from Implementing a Mobile Grocery. Progress in Community Health Partnerships: Research, Education, and Action 2018, 12, 65–72, doi:10.1353/cpr.2018.0007.
91. Sussman, A.L.; Davis, S.M. Integrating Formative Assessment and Participatory Research. American Journal of Health Education 2010, 41, 244–249, doi:10.1080/19325037.2010.10599150.
92. Lee, A.J.; Smith, A.; Bryce, S.; O'Dea, K.; Rutishauser, I.H.E.; Mathews, J.D. Measuring dietary intake in remote australian aboriginal communities. Ecology of Food and Nutrition 1995, 34, 19–31, doi:10.1080/03670244.1995.9991444.
93. Brimblecombe, J.; Ferguson, M.; Barzi, F.; Brown, C.; Ball, K. Mediators and moderators of nutrition intervention effects in remote Indigenous Australia. Brit J Nutr 2018, 119, 1424–1433, doi:10.1017/s0007114518000880.
94. Vandevijvere, S.; Young, N.; Mackay, S.; Swinburn, B.; Gahegan, M. Modelling the cost differential between healthy and current diets: the New Zealand case study. International Journal of Behavioral Nutrition and Physical Activity 2018, 15, 1–10, doi:10.1186/s12966-018-0648-6.
95. Richards, T.J.; Patterson, P.M. Native American Obesity: An Economic Model of the "Thrifty Gene" Theory. American Journal of Agricultural Economics 2006, 88, 542–560, doi:10.1111/j.1467-8276.2006.00878.x.
96. Schiff, R.; Brunger, F. Northern Food Networks: Building Collaborative Efforts for Food Security in Remote Canadian Aboriginal Communities. Journal of Agriculture, Food Systems, and Community Development 2013, 121–138, doi:10.5304/jafscd.2013.033.012.
97. Gawn, G.; Innes, R.; Rausser, G.; Zilberman, D. Nutrient demand and the allocation of time: evidence from Guam. Applied Economics 1993, 25, 811–830, doi:10.1080/00036849300000136.
98. Lee, A.J.; Rainow, S.; Tregenza, J.; Tregenza, L.; Balmer, L.; Bryce, S.; Paddy, M.; Sheard, J.; Schomburgk, D. Nutrition in remote Aboriginal communities: lessons from Mai Wiru and the Anangu Pitjantjatjara Yankunytjatjara Lands. Australian and New Zealand Journal of Public Health 2016, 40, S81–S88, doi:10.1111/1753-6405.12419.

99. Black, A.P.; Vally, H.; Morris, P.; Daniel, M.; Esterman, A.; Karschimkus, C.S.; O'Dea, K. Nutritional impacts of a fruit and vegetable subsidy programme for disadvantaged Australian Aboriginal children. British Journal of Nutrition 2013, 110, 2309–2317, doi:10.1017/s0007114513001700.
100. Redmond, L.C.; Jock, B.; Gadhoke, P.; Chiu, D.T.; Christiansen, K.; Pardilla, M.; Swartz, J.; Platero, H.; Caulfield, L.E.; Gittelsohn, J. OPREVENT (Obesity Prevention and Evaluation of InterVention Effectiveness in NaTive North Americans): Design of a Multilevel, Multicomponent Obesity Intervention for Native American Adults and Households. Current Developments in Nutrition 2019, 3, 81–93, doi:10.1093/cdn/nzz009.
101. Brimblecombe, J.K.; Ferguson, M.M.; Liberato, S.C.; O'Dea, K.; Riley, M. Optimisation Modelling to Assess Cost of Dietary Improvement in Remote Aboriginal Australia. PLoS ONE 2013, 8, e83587, doi:10.1371/journal.pone.0083587.
102. Johnson-Down, L.; Willows, N.; Kenny, T.A.; Ing, A.; Fediuk, K.; Sadik, T.; Chan, H.M.; Batal, M. Optimisation modelling to improve the diets of First Nations individuals. Journal of Nutritional Science 2019, 8, S32, doi:10.1017/jns.2019.30.
103. Matanane, L.; Fialkowski, M.K.; Silva, J.; Li, F.; Nigg, C.; Guerrero, R.T.L.; Novotny, and R. Para I Famagu'on-Ta: Fruit and Vegetable Intake, Food Store Environment, and Childhood Overweight/Obesity in the Children's Healthy Living Program on Guam. Hawai'i Journal of Medicine & Public Health 2017, 76, 225.
104. Gittelsohn, J.; Roache, C.; Kratzmann, M.; Reid, R.; Ogina, J.; Sharma, S. Participatory Research for Chronic Disease Prevention in Inuit Communities. Am J Health Behav 2010, 34, 453–464, doi:10.5993/ajhb.34.4.7.
105. McDonald, E.L.; Bailie, R.S.; Morris, P.S. Participatory systems approach to health improvement in Australian Aboriginal children. Health Promotion International 2017, 32, 62–72, doi:10.1093/heapro/dau003.
106. Goldhar, C.; Ford, J.D.; Berrang-Ford, L. Prevalence of food insecurity in a Greenlandic community and the importance of social, economic and environmental stressors. International Journal of Circumpolar Health 2012, 69, 285–303, doi:10.3402/ijch.v69i3.17616.
107. Rosecrans, A.M.; Gittelsohn, J.; Ho, L.S.; Harris, S.B.; Naqshbandi, M.; Sharma, S. Process evaluation of a multi-institutional community-based program for diabetes prevention among First Nations. Health Education Research 2008, 23, 272–286, doi:10.1093/her/cym031.
108. Curran, S.; Gittelsohn, J.; Anliker, J.; Ethelbah, B.; Blake, K.; Sharma, S.; Caballero, B. Process evaluation of a store-based environmental obesity intervention on two American Indian Reservations. Health Education Research 2005, 20, 719–729, doi:10.1093/her/cyh032.
109. Snowdon, W.; Raj, A.; Reeve, E.; Guerrero, R.L.; Fesaitu, J.; Cateine, K.; Guignet, C. Processed foods available in the Pacific Islands. Globalization and Health 2013, 9, 1–7, doi:10.1186/1744-8603-9-53.
110. Gittelsohn, J.; Anliker, J.A.; Sharma, S.; Vastine, A.E.; Caballero, B.; Ethelbah, B. Psychosocial Determinants of Food Purchasing and Preparation in American Indian Households. Journal of Nutrition Education and Behavior 2006, 38, 163–168, doi:10.1016/j.jneb.2005.12.004.
111. Cunningham-Sabo, L.; Bauer, M.; Pareo, S.; Phillips-Benally, S.; Roanhorse, J.; Garcia, L. Qualitative Investigation of Factors Contributing to Effective Nutrition Education for Navajo Families. Maternal and Child Health Journal 2008, 12, 68–75, doi:10.1007/s10995-008-0333-5.
112. Dammann, K.W.; Smith, C. Race, Homelessness, and Other Environmental Factors Associated with the Food-Purchasing Behavior of Low-Income Women. Journal of the American Dietetic Association 2010, 110, 1351–1356, doi:10.1016/j.jada.2010.06.007.

113. Sowerwine, J.; Mucioki, M.; Sarna-Wojcicki, D.; Hillman, L. Reframing food security by and for Native American communities: a case study among tribes in the Klamath River basin of Oregon and California. Food Security 2019, 11, 579–607, doi:10.1007/s12571-019-00925-y.
114. Burnett, K.; Skinner, K.; Hay, T.; LeBlanc, J.; Chambers, L. Retail food environments, shopping experiences, First Nations and the provincial Norths. Health Promotion and Chronic Disease Prevention in Canada 2017, 37, 333–341, doi:10.24095/hpcdp.37.10.03.
115. Lee, A.J.; Hobson, V.; Katarski, L. Review of the nutrition policy of the Arnhem Land Progress Association. Australian and New Zealand Journal of Public Health 1996, 20, 538–544, doi:10.1111/j.1467-842x.1996.tb01636.x.
116. Rudolph, K.R.; McLachlan, S.M. Seeking Indigenous food sovereignty: origins of and responses to the food crisis in northern Manitoba, Canada. Local Environment 2013, 18, 1079–1098, doi:10.1080/13549839.2012.754741.
117. Jackson, S.L.; VanFrank, B.K.; Lundeen, E.; Uncangco, A.; Alam, L.; King, S.M.C.; Cogswell, M.E. Sodium in Store and Restaurant Food Environments — Guam, 2015. MMWR. Morbidity and Mortality Weekly Report 2016, 65, 510–513, doi:10.15585/mmwr.mm6520a2.
118. Wycherley, T.P.; Ferguson, M.M.; O'Dea, K.; McMahon, E.; Liberato, S.; Brimblecombe, J.K. Store turnover as a predictor of food and beverage provider turnover and associated dietary intake estimates in very remote Indigenous communities. Australian and New Zealand Journal of Public Health 2016, 40, 569–571, doi:10.1111/1753-6405.12571.
119. Mhurchu, C.N.; Blakely, T.; Wall, J.; Rodgers, A.; Jiang, Y.; Wilton, J. Strategies to promote healthier food purchases: a pilot supermarket intervention study. Public Health Nutrition 2007, 10, 608–615, doi:10.1017/s136898000735249x.
120. Rogers, A.; Ferguson, M.M.; Ritchie, J.; Boogaard, C.V.D.; Brimblecombe, J.K. Strengthening food systems with remote Indigenous Australians: stakeholders' perspectives. Health Promotion International 2018, 33, 38–48, doi:10.1093/heapro/daw047.
121. Snowdon, W. Sugar-sweetened beverages in Pacific Island countries and territories: problems and solutions. fizz.org.nz 2014, 20, 43–46.
122. Fehring, E.; Ferguson, M.M.; Brown, C.; Murtha, K.; Laws, C.; Cuthbert, K.; Thompson, K.; Williams, T.; Hammond, M.; Brimblecombe, J.K. Supporting healthy drink choices in remote Aboriginal and Torres Strait Islander communities: a community-led supportive environment approach. Australian and New Zealand Journal of Public Health 2019, 43, 551–557, doi:10.1111/1753-6405.12950.
123. Signal, L.; Walton, M.D.; Mhurchu, C.N.; Maddison, R.; Bowers, S.G.; Carter, K.N.; Gorton, D.; Heta, C.; Lanumata, T.S.; McKerchar, C.W.; et al. Tackling "wicked" health promotion problems: a New Zealand case study. Health Promotion International 2013, 28, 84–94, doi:10.1093/heapro/das006.
124. Lee, A.J.; Lewis, M. Testing the Price of Healthy and Current Diets in Remote Aboriginal Communities to Improve Food Security: Development of the Aboriginal and Torres Strait Islander Healthy Diets ASAP (Australian Standardised Affordability and Pricing) Methods. International Journal of Environmental Research and Public Health 2018, 15, 2912, doi:10.3390/ijerph15122912.
125. Smoyer-Tomic, K.E.; Spence, J.C.; Raine, K.D.; Amrhein, C.; Cameron, N.; Yasenovskiy, V.; Cutumisu, N.; Hemphill, E.; Healy, J. The association between neighborhood socioeconomic status and exposure to supermarkets and fast food outlets. Health & Place 2008, 14, 740–754, doi:10.1016/j.healthplace.2007.12.001.
126. Parker, B.; Burnett, K.; Hay, T.; Skinner, K. The Community Food Environment and Food Insecurity in Sioux Lookout, Ontario: Understanding the Relationships between Food,

Health, and Place. Journal of Hunger & Environmental Nutrition 2019, 44, 1–18, doi:10.1080/19320248.2018.1537867.
127. Ferguson, M.M.; O'Dea, K.; Chatfield, M.; Moodie, M.; Altman, J.; Brimblecombe, J.K. The comparative cost of food and beverages at remote Indigenous communities, Northern Territory, Australia. Australian and New Zealand Journal of Public Health 2016, 40, S21–S26, doi:10.1111/1753-6405.12370.
128. Piltch, E.M.; Shin, S.S.; Houser, R.F.; Griffin, T. The complexities of selling fruits and vegetables in remote Navajo Nation retail outlets: perspectives from owners and managers of small stores. Public Health Nutrition 2020, 23, 1638–1646, doi:10.1017/s1368980019003720.
129. Campbell, M.L.; Diamant, R.M.F.; Macpherson, B.D.; Halladay, J.L. The Contemporary Food Supply of Three Northern Manitoba Cree Communities. C J Public Health 1997, 88, 105–108, doi:10.1007/bf03403871.
130. Magnus, A.; Cobiac, L.; Brimblecombe, J.K.; Chatfield, M.; Gunther, A.; Ferguson, M.M.; Moodie, M. The cost-effectiveness of a 20% price discount on fruit, vegetables, diet drinks and water, trialled in remote Australia to improve Indigenous health. PLoS ONE 2018, 13, e0204005, doi:10.1371/journal.pone.0204005.
131. Magnus, A.; Moodie, M.L.; Ferguson, M.M.; Cobiac, L.J.; Liberato, S.C.; Brimblecombe, J.K. The economic feasibility of price discounts to improve diet in Australian Aboriginal remote communities. Australian and New Zealand Journal of Public Health 2016, 40, S36–S41, doi:10.1111/1753-6405.12391.
132. Beaumier, M.C.; Ford, J.D.; Tagalik, S. The food security of Inuit women in Arviat, Nunavut: the role of socio-economic factors and climate change. Polar Record 2015, 51, 550–559, doi:10.1017/s0032247414000618.
133. Watson, Z.A.; Shanks, C.B.; Miles, M.P.; Rink, E. The Grocery Store Food Environment in Northern Greenland and Its Implications for the Health of Reproductive Age Women. Journal of Community Health 2018, 43, 175–185, doi:10.1007/s10900-017-0400-1.
134. Brimblecombe, J.K.; O'Dea, K. The role of energy cost in food choices for an Aboriginal population in northern Australia. Med J Australia 2009, 190, 549–551, doi:10.5694/j.1326-5377.2009.tb02560.x.
135. Morrison, N.; Dooley, J. The Sioux Lookout Diabetes Program: diabetes prevention and management in northwestern Ontario. International Journal of Circumpolar Health 1998, 57 Suppl 1, 364–369.
136. Genuis, S.K.; Willows, N.D.; Jardine, C. Through the lens of our cameras: children's lived experience with food security in a Canadian Indigenous community. Child: Care, Health and Development 2015, 41, 600–610, doi:10.1111/cch.12182.
137. Calancie, L.; Cooksey-Stowers, K.; Palmer, A.; Frost, N.; Calhoun, H.; Piner, A.; Webb, K. Toward a Community Impact Assessment for Food Policy Councils: Identifying Potential Impact Domains. Journal of Agriculture, Food Systems, and Community Development 2018, 8, 1–14, doi:10.5304/jafscd.2018.083.001.
138. Lambden, J.; Receveur, O.; Marshall, J.; Kuhnlein, H.V. Traditional and market food access in Arctic Canada is affected by economic factors. International Journal of Circumpolar Health 2006, 65, 331–340, doi:10.3402/ijch.v65i4.18117.
139. Pollard, C.M.; Nyaradi, A.; Lester, M.; Sauer, K. Understanding food security issues in remote Western Australian Indigenous communities. Health Promotion Journal of Australia 2014, 25, 83–89, doi:10.1071/he14044.
140. Stroink, M.L. Understanding Local Food Behaviour and Food Security in Rural First Nation Communities: Implications for Food Policy. The Journal of Rural and Community Development 2012, 7, 65–82.

141. Eyles, H.; Rodgers, A.; Mhurchu, C.N. Use of electronic sales data to tailor nutrition education resources for an ethnically diverse population. Journal of human nutrition and dietetics : the official journal of the British Dietetic Association 2010, 23, 38–47, doi:10.1111/j.1365-277x.2009.01006.x.
142. Brimblecombe, J.K.; Liddle, R.; O'Dea, K. Use of point-of-sale data to assess food and nutrient quality in remote stores. Public Health Nutrition 2012, 16, 1159–1167, doi:10.1017/s1368980012004284.
143. Skinner, K.; Hanning, R.M.; Sutherland, C.; Edwards-Wheesk, R.; Tsuji, L.J.S. Using a SWOT Analysis to Inform Healthy Eating and Physical Activity Strategies for a Remote First Nations Community in Canada. Am J Health Promot 2012, 26, e159–e170, doi:10.4278/ajhp.061019136.
